# Proline-rich protein PRR19 functions with cyclin-like CNTD1 to promote meiotic crossing over in mouse

**DOI:** 10.1038/s41467-020-16885-3

**Published:** 2020-06-18

**Authors:** Anastasiia Bondarieva, Kavya Raveendran, Vladyslav Telychko, H. B. D. Prasada Rao, Ramya Ravindranathan, Chrysoula Zorzompokou, Friederike Finsterbusch, Ihsan Dereli, Frantzeskos Papanikos, Daniel Tränkner, Alexander Schleiffer, Ji-Feng Fei, Anna Klimova, Masaru Ito, Dhananjaya S. Kulkarni, Ingo Roeder, Neil Hunter, Attila Tóth

**Affiliations:** 1 Institute of Physiological Chemistry, Faculty of Medicine Carl Gustav Carus, Technische Universität Dresden, Fetscherstraße 74, 01307 Dresden, Germany; 2 Howard Hughes Medical Institute, University of California Davis, Davis, CA USA; 3 Department of Microbiology & Molecular Genetics, University of California Davis, Davis, CA USA; 4 Research Institute of Molecular Pathology (IMP), Campus-Vienna-Biocenter 1, Vienna BioCenter (VBC), 1030 Vienna, Austria; 5 Institute of Molecular Biotechnology (IMBA), Dr. Bohr-Gasse 3, Vienna BioCenter (VBC), 1030 Vienna, Austria; 6 Institute for Brain Research and Rehabilitation, South China Normal University, 510631 Guangzhou, China; 7 National Center for Tumor Diseases (NCT), Dresden, Germany; 8 Institute for Medical Informatics and Biometry, Faculty of Medicine Carl Gustav Carus, Technische Universität Dresden, Dresden, Germany; 9 Department of Molecular & Cellular Biology, University of California Davis, Davis, CA USA

**Keywords:** Checkpoints, Meiosis, Chromosomes, DNA damage and repair, DNA recombination

## Abstract

Orderly chromosome segregation is enabled by crossovers between homologous chromosomes in the first meiotic division. Crossovers arise from recombination-mediated repair of programmed DNA double-strand breaks (DSBs). Multiple DSBs initiate recombination, and most are repaired without crossover formation, although one or more generate crossovers on each chromosome. Although the underlying mechanisms are ill-defined, the differentiation and maturation of crossover-specific recombination intermediates requires the cyclin-like CNTD1. Here, we identify PRR19 as a partner of CNTD1. We find that, like CNTD1, PRR19 is required for timely DSB repair and the formation of crossover-specific recombination complexes. PRR19 and CNTD1 co-localise at crossover sites, physically interact, and are interdependent for accumulation, indicating a PRR19-CNTD1 partnership in crossing over. Further, we show that CNTD1 interacts with a cyclin-dependent kinase, CDK2, which also accumulates in crossover-specific recombination complexes. Thus, the PRR19-CNTD1 complex may enable crossover differentiation by regulating CDK2.

## Abstract (web summary)

Crossing over is a critical process during meiosis, although the regulation of this process still remains somewhat elusive. Here, the authors show that PRR19 partners with CNTD1 to enable formation of crossover-specific recombination complexes in mouse germ cells.

## Introduction

Meiotic recombination generates reciprocal exchanges, called crossovers, between homologous chromosomes (homologs) during the prophase of the first meiotic division. Crossovers form the basis of physical inter-homolog linkages, called chiasmata, which combine homologs into bivalent chromosomes. Chiasmata are mechanistically required for correct chromosome segregation in the first meiotic division. Hence, chiasmata and underlying crossovers form between each pair of homologs in most taxa, including mammals.

Distinct steps in crossover formation associate with well-defined stages of meiosis (reviewed in refs. ^1,2^). In the preleptotene stage, premeiotic DNA replication generates sister chromatids within each chromosome. Recombination takes place along linear chromatin structures, called chromosome axes, which assemble on cohesin cores of sister chromatid pairs. Recombination is initiated in the leptotene by programmed DNA double-stranded breaks (DSBs) along short stretches of forming chromosome axes^2^. Following DNA end resection, the resultant single-stranded DNA tails invade homologous DNAs with the help of strand-exchange proteins RAD51 and DMC1^1^. In the zygotene, DNA-strand invasions promote parallel juxtaposition of homolog axes contemporaneously with axes maturation into contiguous structures stretching between chromosome ends. Zipper-like synaptonemal complexes (SCs)^1^ form between juxtaposed homolog axes and facilitate post-strand-invasion steps of recombination. Once all homolog pairs formed SCs, meiocytes enter the pachytene stage, where DSBs are repaired and crossovers form. Crossovers keep homologs tethered in bivalents upon disassembly of SCs (diplotene stage) and progression to metaphase I.

Meiocytes form many more DSBs (~200–400 in mice) than crossovers (~20–30, or one-to-two per homolog pair), hence, most DNA-strand-invasion intermediates are resolved without crossover formation (called non-crossovers)^1^. It is unanswered how a subset of DNA-strand-invasion intermediates is selected and differentiated to form crossovers. Two classes of mechanisms transform DNA-strand-invasion intermediates into crossovers. Most crossovers (90–95%) are generated by the class I pathway, which relies on the putative crossover-resolvase MutLγ complex (MLH1/MLH3)^3–9^. This pathway is subject to poorly understood regulatory mechanisms that differentiate at least one recombination intermediate into crossover on each chromosome (crossover assurance), and prevent the formation of crossovers in close proximity to one another (crossover interference; reviewed in ref. ^1^). The remaining crossovers depend on the processing of recombination intermediates by structure-specific nucleases, e.g., MUS81-EME1, and are not subjected to the same controls as class I crossovers^4,5,10^.

Differential stabilisation of strand-invasion intermediates underlies the choice between differentiation into crossovers or non-crossovers. Initially, the MutSγ complex (MSH4/MSH5) stabilises DNA-strand invasions to permit efficient homolog synapsis in the early prophase^11–13^. Thereafter, in the early–mid pachytene, MutSγ enacts a specific role in crossover differentiation. A positive feedback between MutSγ and the SUMO-ligase RNF212^14^ is thought to stabilise a subset of strand-exchange intermediates conferring competence for crossover formation. The RNF212-MutSγ-positive feedback is hypothesised to effect a competition between strand-invasion intermediates for RNF212/MutSγ, leading to gradual restriction of RNF212/MutSγ to one or two recombination sites per chromosome^14^. Whereas recombination intermediates that retain RNF212/MutSγ till mid pachytene commit to crossover formation, intermediates that lose RNF212/MutSγ are repaired as non-crossovers during the early/mid pachytene.

Beyond RNF212 and MutSγ, the HEI10 ubiquitin ligase^15^ and the cyclin-like CNTD1^16^ are crucial for crossover differentiation and the underlying reduction in the number of stabilised recombination complexes in mid pachytene. In the absence of HEI10 or CNTD1, RNF212/MutSγ persists on most recombination intermediates, and RNF212/MutSγ-associated intermediates fail to produce crossovers. Thus, HEI10 and CNTD1 seem to enable crossover differentiation by limiting RNF212/MutSγ-mediated stabilisation of recombination intermediates. Whereas it is unknown how CNTD1 functions, HEI10^15,17^ is inferred to destabilise recombination intermediates by ubiquitinating recombination proteins and targeting them for degradation by chromosome axis-bound 26S proteasomes^15,18,19^.

Following their differentiation in early–mid pachytene, crossover-committed recombination complexes mature by losing RNF212/MutSγ and accumulating HEI10^15,18^, the cell-cycle kinase CDK2^20^ and MutLγ^21^ in mid/late pachytene. MutLγ is thought to resolve the recombination complex-associated strand-invasion intermediates as crossovers^3,4,6–9,22^. In contrast, it is unclear how CDK2, HEI10 and CNTD1 contribute to crossover maturation beyond their upstream roles in homologous synapsis (CDK2)^23,24^ and crossover differentiation (HEI10 and CNTD1)^15,16^.

Here, we identify a meiosis-specific protein, PRR19, which forms a complex with CNTD1. We find that both proteins accumulate in crossover-specific recombination complexes, and their loss leads to similar impairment in crossover differentiation and DSB repair. Our data suggest that the PRR19–CNTD1 protein complex regulates CDK2, which may underlie PRR19–CNTD1 function in meiotic recombination.

## Results

### PRR19 marks crossover precursors in pachytene meiocytes

To identify potential meiotic recombination proteins, we profiled gene expression in mouse gonads, reviewed ENCODE transcriptome datasets^25^, and looked for genes that are expressed preferentially in gonads which contain meiocytes in the first meiotic prophase^26,27^. *Proline-rich 19* (*Prr19*) was one of the identified gonad-expressed genes (Supplementary Fig. 1a–c), whose homologs were found in most metazoans except Ecdysozoa (Supplementary Fig. 2). *Prr19* encodes a 366 amino acid protein with four functionally uncharacterised conserved motives.

**Fig. 1:**
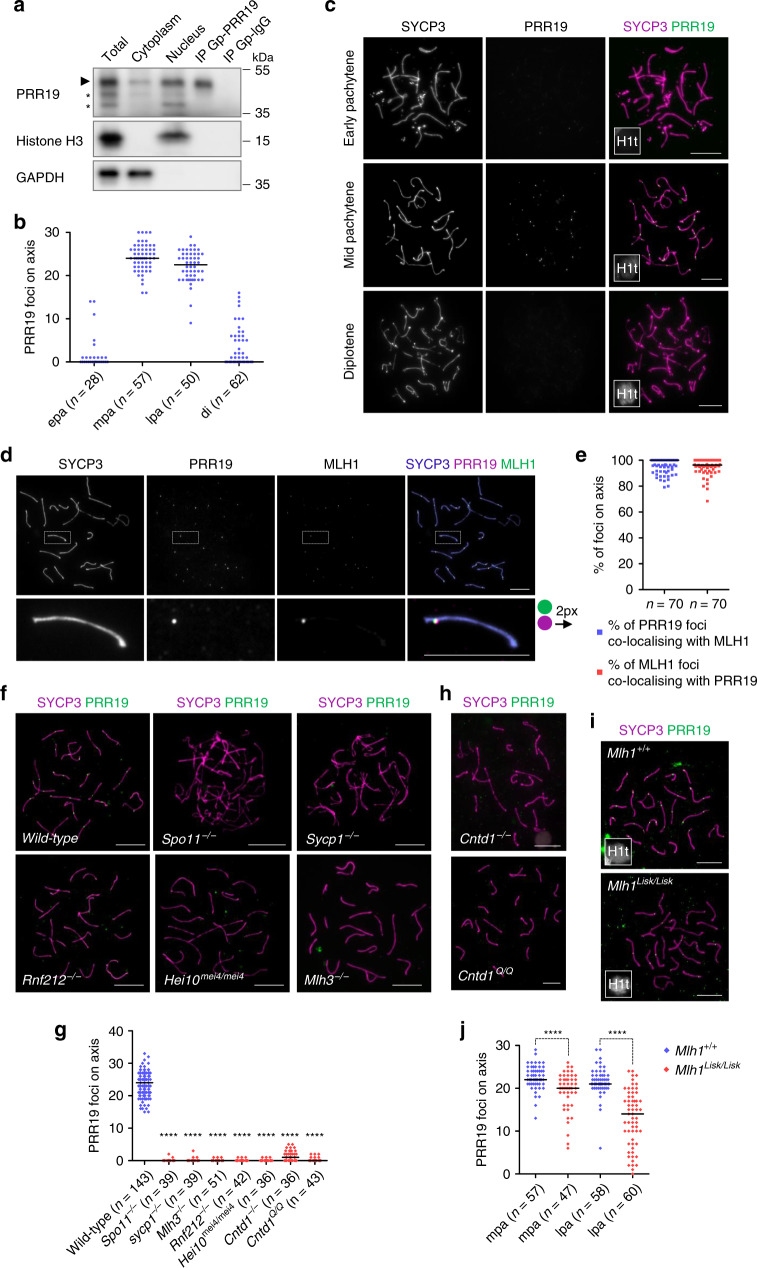
PRR19 localises to crossover-specific recombination complexes. **a** Immunoblot of protein extracts from testes of adult mice; total lysate, cytoplasmic and nuclear fractions and immunoprecipitates with guinea pig anti-PRR19 (IP Gp-PRR19) or non-specific (IP Gp–IgG) antibodies are shown. Upper panel: immunoblot by Gp-PRR19 antibodies. Arrowhead marks presumed PRR19 band. Asterisks mark unspecific protein bands, which varied with the age of analysed mice and antibody batch (see Figs. 2c, d, 7h). Molecular weight marker positions are indicated. Nuclear histone H3 (middle panel) and cytoplasmic GAPDH (bottom panel) controlled for fractionation. **b** Quantification of axis-associated PRR19 foci detected by Gp-PRR19 antibody in wild-type spermatocytes in early (epa), mid (mpa), late pachytene (lpa) and diplotene (di). *n* = numbers of analysed cells from three animals; medians (bars) are 0 (epa), 24 (mpa), 22.5 (lpa) and 0 (di). **c**, **d**, **f**, **h**, **i** Immunofluorescent staining of nuclear surface-spread (**c**, **d**) wild-type spermatocytes or (**f**, **h**, **i**) spermatocytes of indicated genotypes from adult mice. Bars, 10 µm; in **d** lower panel, 5 µm. Histone H1t (**c**, **i**, insets, H1t) marks prophase stages after early pachytene. **d** Boxed autosome of a mid/late pachytene spermatocyte is enlarged in the bottom panel, where the PRR19 signal is shifted to the right by two pixels for better visualisation of PRR19-MLH1 co-localisation. **e** Quantification of co-localisation between PRR19 and MLH1 foci on chromosome axes in mid/late pachytene wild-type spermatocytes. *n* = numbers of analysed cells from three animals, medians (bars) are 100% (blue data set) and 96.3% (red data set). **g** Quantification of axis-associated PRR19 foci in pachytene or pachytene-like spermatocytes from wild types and indicated mutants. **j** Quantification of axis-associated PRR19 foci in mid (mpa) and late pachytene (lpa) spermatocytes of wild-type and *Mlh1*^*Lisk/Lisk*^ mice. **g**, **j** *n* = numbers of analysed cells from at least two animals; medians (bars) are (**g**) 24 in wild-type, 0 in *Spo11*^*−/−*^*, Sycp1*^*−/−*^*, Mlh3*^*−/−*^*, Rnf212*^−*/−*^*, Hei10*^*mei4/mei4*^ and *Cntd1*^*Q/Q*^, 1 in *Cntd1*^−*/−*^, (**j**, mpa) 22 and 20, (**j**, lpa) 21 and 14 in wild-type and *Mlh1*^*Lisk/Lisk*^, respectively. Mann–Whitney *U* test, **** indicates (**g**) *P* < 2.2E-16, (**j**, mpa) *P* = 5.59E-05 and (**j**, lpa) *P* = 2.43E-13. Source data are provided as a Source Data file.

PRR19 was enriched in the nucleus (Fig. 1a), hence, we tested if PRR19 localised to chromosomes. Reproducible PRR19-specific immunolabeling was detected on chromosomes only in mid/late pachytene spermatocytes as identified by histone H1t expression, which marks spermatocytes from mid pachytene onwards^28^ (see ‘Methods' in ref. ^27^ for the staging meiotic prophase). PRR19 was detected in one or two foci on each synapsed chromosome in spermatocytes (Fig. 1b, c; Supplementary Fig. 1d). Similar PRR19 staining was observed in foetal oocytes at 18 days post coitum (dpc), where most oocytes were in the mid/late pachytene (Supplementary Fig. 1e). PRR19 localisation resembled the localisation of crossover-specific recombination complexes, and PRR19 co-localised with the crossover marker MLH1 in spermatocytes (Fig. 1d, e). Further, whereas CDK2 localises to crossover-specific interstitial autosomal sites, telomeres and unsynapsed axes of sex chromosomes^20^, PRR19 co-localised with CDK2 only at interstitial foci (Supplementary Fig. 1f, g). Thus, PRR19 marks crossover-specific recombination sites in meiocytes.

Consistent with PRR19 association with crossover-specific recombination complexes, PRR19 foci were absent from spermatocytes of DSB-defective (*Spo11*^−*/−*^)^29^ or synapsis-defective (*Sycp1*^*−/*−^)^30^ mice (Fig. 1f, g). To further test if PRR19 foci required crossover-specific precursors, we analysed four crossover differentiation-defective mouse strains, the published *Rnf212*^*−/−*^^14^ and *Hei10*^*mei4/mei4*^ ^17^ lines and two newly generated CNTD1-deficient lines (this study).

Our *Cntd1* lines carried either a frameshift mutation (*Cntd1*^*−/−*^) or a mutation that caused mis-splicing (*Cntd1*^*Q/Q*^; Supplementary Fig. 3a, c). Whereas testes of the former line lacked CNTD1 (Supplementary Fig. 3b), the latter line expressed a CNTD1 protein lacking four amino acids in the predicted first alpha helix of the cyclin box of CNTD1 (Supplementary Fig. 3b, d, e). Similar to earlier published CNTD1-deficient mice (*Cntd1*^*GT/GT*^)^16^, both *Cntd1*^*−/*−^ and *Cntd1*^*Q/Q*^ mice failed in crossover differentiation. Thus, MLH1 foci did not form (Supplementary Fig. 4a, b), and RNF212 foci persisted in high numbers instead of condensing down to one or two crossover-specific foci per chromosome in the mid/late pachytene (Supplementary Fig. 4c, d). This was accompanied by a delay in DSB repair, as indicated by persisting RPA foci and autosomal γH2AX flares in late pachytene nuclei (Supplementary Fig. 4e–j). PRR19 foci were diminished in the crossover differentiation-defective *Rnf212*^*−/*−^*, Hei10*^*mei4/mei4*^*, Cntd1*^−*/*−^ and *Cntd1*^*Q/Q*^ mice (Fig. 1f-h), prompting us to test if crossover maturation was also required for PRR19 localisation.

Whereas maturation of crossover precursors into crossovers requires MutLγ, differentiation of crossover precursors from non-crossovers does not, as judged by the paring down of RNF212/MSH4 foci to a few per chromosome in mid pachytene *Mlh3*^*−/−*^ spermatocytes^14,31^. Whereas MLH1 foci depend on MLH3, MLH3 focus formation is only enhanced by MLH1^6,21^. Thus, MLH3-deficient spermatocytes lack both MLH1 and MLH3 functions at crossover precursors. In contrast, MLH1-deficient spermatocytes may retain MLH1-independent functions of MLH3 at crossover precursors. Whereas the median of PRR19 focus numbers was zero in *Mlh3*^*−/−*^ spermatocytes (Fig. 1f, g), PRR19 foci were present in MLH1-deficient spermatocytes, albeit at lower numbers than in wild-type (Fig. 1i, j). Thus, PRR19 recruitment to crossover precursors requires MLH3, but not MLH1 or full MutLγ functionality.

Together, these observations identify PRR19 as a new marker of crossover-specific recombination intermediates.

### PRR19 is required for fertility in mice

**Fig. 2:**
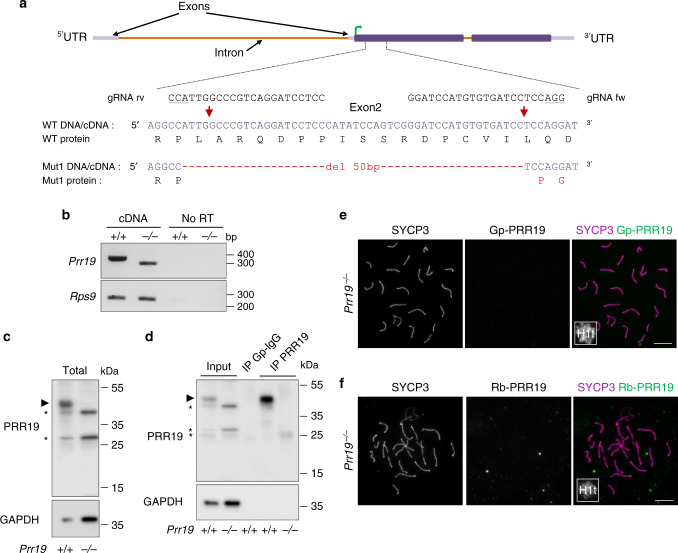
The expression of PRR19 is disrupted in PRR19-deficient mice. **a** Schematics of the *Prr19* locus show exons, introns, translated regions (dark lilac boxes), untranslated regions (5′- and 3′-UTR, light lilac boxes) and the start of open reading frame (green arrow) in exon 2. Expanded view of exon 2 shows guide RNA sequences (gRNA rv and gRNA fw with PAMs underlined) used for CRISPR/Cas9 editing, the genomic DNA/cDNA sequence of the edited region and the corresponding protein sequences in wild-type (WT) and *Prr19* mutant line (Mut1). DNA break sites are marked by red arrows; red text colour marks mutated DNA and protein sequences. The same 50-bp deletion was detected in both genomic DNA and cDNA from testis. **b** Agarose gel pictures show RT-PCRs using primers that match (upper panel) either *Prr19* exon 1 and exon 2 sites flanking the targeted region, (lower panel) or, as a control, cDNAs of the 40S ribosomal protein S9 (*Rps9*). The total testis RNAs of adult wild-type (+/+) and PRR19-deficient (−/−) mice served as templates. Samples without reverse transcriptase (no RT) show RT-PCR specificity. DNA length marker positions are indicated. **c**, **d** Immunoblot analysis of (**c**) total protein extracts (Total), or (**d**) anti-PRR19 immunoprecipitations from testes of adult wild-type (+/+) and *Prr19*^*−/−*^ (−/−) mice. **d** Pictures show inputs for immunoprecipitations, control immunoprecipitates with guinea pig IgG isotype antibodies (IP Gp–IgG) and immunoprecipitates with guinea pig antibodies against the *C*-terminal T183-Y366 peptide of PRR19 (IP PRR19). **c**, **d** Upper and lower panels show detection of proteins by guinea pig anti-PRR19 antibodies and, as loading control, anti-GAPDH antibodies, respectively. Arrowhead marks presumed band of PRR19 (40 kDa), asterisks mark unspecific protein bands in upper panels. Molecular weight marker positions are indicated. **e**, **f** Immunofluorescence showing SYCP3, PRR19 and histone H1t (miniaturised image) in nuclear surface-spread spermatocytes of adult *Prr19*^*−/−*^ mice. Images show stainings with anti-PRR19 antibodies raised either in guinea pig (**e**) or rabbits (**f**). Refer to Fig. 1a and Supplementary Fig. 1d for the corresponding stainings in wild-type. Bars, 10 µm. Source data are provided as a Source Data file.

To examine PRR19 functions, we generated two PRR19-deficient mouse lines, Mut1 and Mut2, by CRISPR/Cas9-mediated editing (Fig. 2a, b; Supplementary Fig. 5a). Owing to the similarities of phenotypes in these lines, we performed detailed analysis only in Mut1 (see Supplementary Fig. 5b–e for Mut2 phenotypes), where the *Prr19* open reading frame was disrupted after the 39th codon. PRR19 was undetectable in testis extracts or surface-spread spermatocytes of homozygous Mut1 mice (hereafter *Prr19*^−*/−*^, Fig. 2c–f), indicating that Mut1 is a loss-of-function allele. *Prr19*^*−/−*^ mice lacked obvious somatic defects, but both sexes were infertile; breeding of five *Prr19*^*−/−*^ males and five *Prr19*^*−/−*^ females with fertile partners produced no pups after 175 and 114 breeding weeks, respectively.

### Depletion of PRR19-deficient spermatocytes in the late prophase

**Fig. 3:**
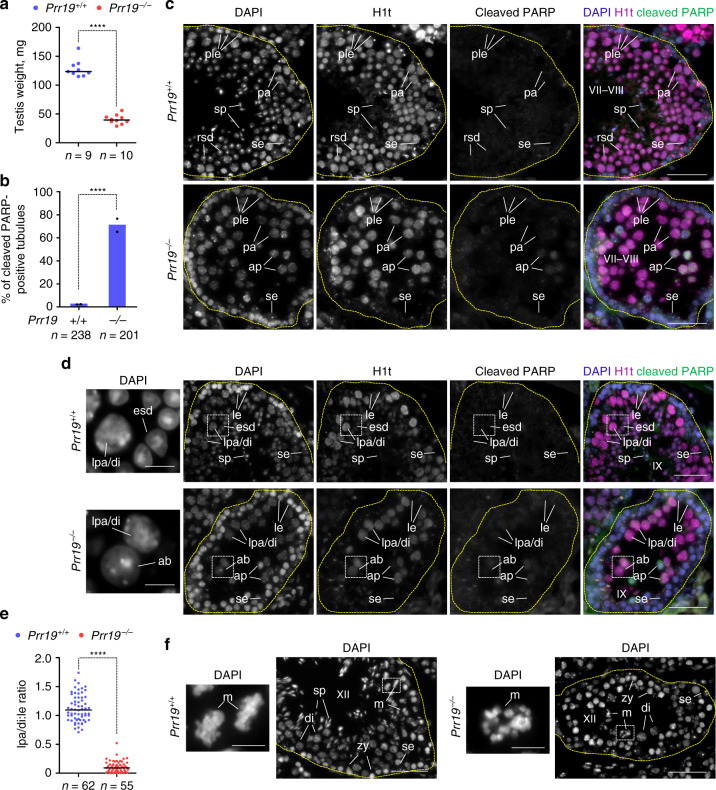
Depletion of late prophase spermatocytes in PRR19-deficient males. **a** The average single testis weights in adult wild-type and PRR19-deficient mice. The number of analysed animals (*n*) and medians (bars, wild-type, 123.5 mg, *Prr19*^−/−^, 39.55 mg) are indicated. Mann–Whitney *U* test calculated *P* = 2.17E-05. **b** Quantification of seminiferous tubules that contain cleaved PARP-positive apoptotic spermatocytes in the pachytene/diplotene layer. Graph shows datapoints and weighted averages of percentages of seminiferous tubules. *n* = number of tubules counted in two independent experiments. An analysis of deviance using the likelihood-ratio test based on the chi-squared distribution was used to calculate if the proportion of apoptotic seminiferous tubules is significantly altered by the loss of PRR19, *P* = 2.2E-16 (****). **c**, **d**, **f** Images show DNA staining by DAPI and (**c**, **d**) immunostaining of histone H1t (staging marker of seminiferous tubules) and cleaved PARP (marker of apoptosis) in sections of testes from adult wild-type and *Prr19*^−*/−*^ mice. Seminiferous tubules in epithelial cycle stages VII–VIII (**c**), IX (**d**) and XII (**f**) are shown; see ‘Methods' for staging. Sertoli cells (se), preleptotene (ple), leptotene (le), zygotene (zy), pachytene (pa), late pachytene (lpa), diplotene (di) and metaphase (m) spermatocytes, round (rsd) and elongating (esd) spermatids, sperm (sp), H1t-positive cells with abnormal nucleus morphology (ab) and apoptotic spermatocytes (ap) are marked. Outlines of tubules (yellow dashed line) and tubule stages are indicated. Enlarged insets show abnormal H1t-positive cells in *Prr19*^−*/−*^ and corresponding spermatids in wild-type seminiferous tubules (side panel, **d**), or misaligned metaphase chromosomes in *Prr19*^*−/−*^ and orderly metaphase plates in wild-type seminiferous tubules (**f**). (**c**, **d**, **f**) Bars, 50 µm; enlarged insets (**d**, **f**) 10 µm. **e** The ratios of late pachytene-diplotene to leptotene spermatocyte numbers (lpa/di:le) were quantified in stage IX seminiferous tubules in testis sections from adult wild-type and PRR19-deficient mice. *n* = numbers of analysed tubules from six (wild-type) or five (*Prr19*^−/−^) animals; medians (bars, wild-type, 1.09, *Prr19*^−/−^, 0.09) were compared using Mann–Whitney *U* test, *P* < 2.2E-16 (****). Source data are provided as a Source Data file.

Testis weight was significantly lower in *Prr19*^*−/−*^ mice than wild-type (Fig. 3a), and *Prr19*^*−/*−^ testes were depleted of post-meiotic cells (Fig. 3c, d, f). Detection of an apoptosis marker, cleaved PARP, in histological testis sections revealed much higher levels of apoptosis in *Prr19*^*−/−*^ testes as compared with wild-type (Fig. 3b). Specifically, apoptosis was elevated in histone H1t-positive pachytene and diplotene spermatocytes in stages V–XII of the seminiferous epithelial cycle (Fig. 3c; Supplementary Table 1, see ‘Methods' for seminiferous tubule staging). Apoptosis peaked in late pachytene spermatocytes in epithelial cycle stages VII–VIII (Supplementary Table 1), resulting in 12-fold fewer pachytene or diplotene cells in epithelial cycle stage IX in *Prr19*^−*/−*^ testes relative to wild-type (Fig. 3d, e). *Prr19*^*−/−*^ spermatocytes that survived beyond diplotene entered an abnormal meiotic metaphase I, where homologs failed to congress, contrasting the orderly chromosome alignment seen in wild-type (Fig. 3f). Despite chromosomal misalignment in spermatocytes, *Prr19*^*−/−*^ seminiferous tubules occasionally contained abnormally large round spermatid-like cells, which may represent post-meiotic cells after non-productive meiotic nuclear divisions (Fig. 3d, enlarged images). These presumptive post-meiotic cells never gave rise to morphologically normal elongated spermatids or sperm.

The apoptosis of *Prr19*^*−/*−^ spermatocytes was consistent with PRR19 possibly functioning in recombination. However, a lack of apoptotic spermatocytes in the seminiferous epithelial cycle stages I–IV argued against PRR19 involvement in synapsis or early recombination steps that promote synapsis, because synapsis defects trigger spermatocyte apoptosis in the epithelial cycle stage IV^32–34^. To verify this conclusion, we examined chromosome axes and SCs in *Prr19*^−*/−*^ spermatocytes. Corresponding stages of axis and SC development were observed in similar fractions of *Prr19*^*−/−*^ and wild-type spermatocytes (Supplementary Fig. 6a, b), indicating proficient axis formation, homolog pairing and synapsis in *Prr19*^*−/*−^ spermatocytes. Whereas both autosomes and sex chromosomes synapsed efficiently, sex chromosomes paired only transiently in *Prr19*^*−/−*^ spermatocytes. The heterologous X and Y sex chromosomes pair only in their short homologous pseudo-autosomal regions (PARs). PARs are held together by synapsis from early to late pachytene and by chiasmata thereafter in recombination-proficient mice. Whereas PARs were connected by SCs in early/mid pachytene in both wild-type and *Prr19*^*−/−*^ spermatocytes, most *Prr19*^−*/*−^ spermatocytes lost PAR connections once PAR-associated SCs disassembled in the late pachytene (Supplementary Fig. 6c, d). This suggests a lack of PAR-associated chiasmata in *Prr19*^−*/*−^ spermatocytes.

### PRR19 is required for efficient crossover formation

**Fig. 4:**
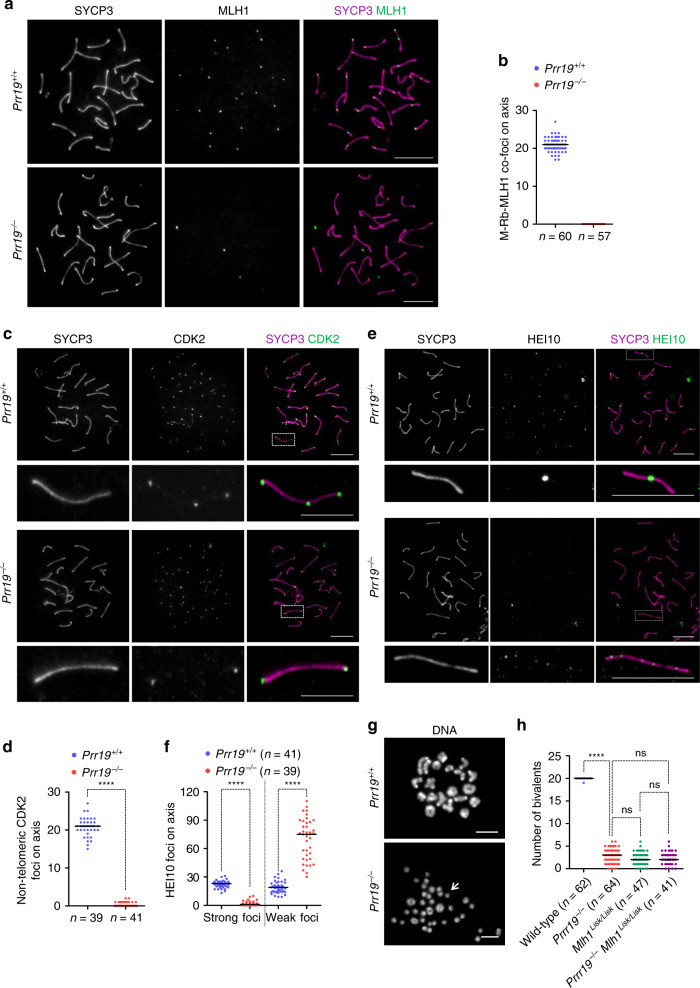
PRR19 is required for efficient crossover formation. **a**, **c**, **e** Chromosome axis (SYCP3) and crossover markers (**a**) MLH1, (**c**) CDK2 or (**e**) HEI10 were detected by immunofluorescence in spreads of mid pachytene spermatocytes. **c**, **e** Bottom panels of each genotype show enlarged insets. Bars, 10 µm; in enlarged insets, 5 µm. **b** Quantification of MLH1 foci in mid/late pachytene spermatocytes. MLH1 was detected by two distinct antibodies raised in mouse and rabbit. Only the axis-associated foci detected by both antibodies were quantified. **d** Quantification of axis-associated interstitial CDK2 foci in mid pachytene spermatocytes. **f** Quantification of axis-associated HEI10 foci in mid/late pachytene spermatocytes. Strong foci of HEI10, which are thought to mark crossover-specific recombination complexes, were quantified separately from weak HEI10 foci along the axis. **b**, **d**, **f** *n* = numbers of analysed cells from two animals; medians (bars) were compared with Mann–Whitney *U* test, when possible, and are as follows in wild-type and *Prr19*^*−/−*^: **b** 21 and 0 MLH1 foci, **d** 21 and 0 interstitial CDK2 foci (*P* = 2.59E-15), **f** 23 and 1 strong HEI10 foci (*P* = 7.26E-15), 19 and 75 weak HEI10 foci (P = 1.25E-14), respectively. **** indicates *P* < 0.0001. **g** DNA was labelled with Hoechst 33342 in spread diakinesis/metaphase I spermatocytes. Arrow indicates univalent in *Prr19*^*−/−*^ cell. Bars, 20 µm. **h** Quantification of bivalents in spread diakinesis/metaphase I spermatocytes from mice of indicated genotypes. *n* = numbers of analysed cells from three (wild-type and *Prr19*^*−/−*^) or two (*Mlh1*^*Lisk/Lisk*^ and *Prr19*^−*/−*^ *Mlh1*^*Lisk/Lisk*^) mice; medians (bars) are indicated. Median bivalent numbers are as follows: 20 in wild-type, 3 in *Prr19*^−*/−*^, 2 in *Mlh1*^*Lisk/Lisk*^ and *Prr19*^−*/*−^ *Mlh1*^*Lisk/Lisk*^. Results of Mann–Whitney *U* test are shown. **** indicates *P* < 2.2E-16 between wild-type and *Prr19*^*−/*−^, ns indicates no significance between *Prr19*^*−/−*^ and *Mlh1*^*Lisk/Lisk*^ (*P* = 0.0636), between *Prr19*^*−/−*^ and *Prr19*^*−/−*^ *Mlh1*^*Lisk/Lisk*^ (*P* = 0.0763) and between *Mlh1*^*Lisk/Lisk*^ and *Prr19*^−*/−*^ *Mlh1*^*Lisk/Lisk*^ (*P* = 0.9966). Source data are provided as a Source Data file.

Localisation patterns of PRR19 and the defective pairing of PARs in *Prr19*^−/−^ spermatocytes suggested a pro-crossover role for PRR19. Hence, we tested if PRR19 was required for class I crossover-specific recombination complexes, which are marked by MutLγ, CDK2 and HEI10 in mid/late pachytene spermatocytes^3,6,15,20,21^. Whereas most, if not all, chromosomes obtained one or two MutLγ foci in wild-type, no MLH1 foci and very few if any bright MLH3 foci were detected in *Prr19*^−*/*−^ spermatocytes (Fig. 4a, b; Supplementary Fig. 7a, b). Also, *Prr19*^−*/−*^ spermatocytes formed CDK2 foci only at telomeres, but not in interstitial crossover sites (Fig. 4c, d). The HEI10 ubiquitin ligase formed ~23 bright foci, likely representing crossover-specific complexes, and slightly lower numbers of additional weak foci in wild-type mid/late pachytene spermatocytes (Fig. 4e, f). By contrast, *Prr19*^−*/*−^ spermatocytes lost most strong HEI10 foci, but contained approximately four-fold more weak HEI10 foci then wild-type (Fig. 4e, f). Thus, PRR19 is required for the formation of mature class I crossover-specific recombination complexes on chromosomes in the mid/late pachytene.

Hence, we tested if homolog pairs are held together by chiasmata in bivalent chromosomes in *Prr19*^*−/−*^ spermatocytes at the diakinesis/metaphase I stages. Whereas 20 bivalents were observed in wild-type, the median bivalent number was only three in *Prr19*^*−/−*^ spermatocytes (Fig. 4g, h), indicating that most, but not all, crossovers require PRR19. Bivalent numbers were similar in *Prr19*^−*/*−^*, Mlh1*^*Lisk/Lisk*^ (see ref. ^3^ for *Mlh1*^*Lisk*^ allele) and double-mutant *Prr19*^*−/−*^ *Mlh1*^*Lisk/Lisk*^ mice (Fig. 4h), indicating that PRR19 and MutLγ act in the same crossover pathway.

### DSB repair is delayed in the absence of PRR19

We wondered if PRR19 functions during the differentiation of crossover-specific recombination sites, or it only acts in the maturation of crossover-committed precursors. Crossover differentiation failure manifests in two different ways. All MSH4-positive recombination foci disappear by the mid pachytene, and recombination intermediates resolve as non-crossovers due to a lack of stabilisation, as seen in *Rnf212*^−*/−*^ mice^14^. Alternatively, excessive stabilisation impairs the resolution of recombination intermediates as crossovers or non-crossovers, which manifests in a persistence of unrepaired DSBs and abnormally high numbers of RNF212/MSH4-marked recombination foci beyond mid pachytene, as seen in HEI10- and CNTD1*-*deficient meiocytes^15,16,18^. In contrast, if only crossover maturation is defective, paring down of RNF212/MSH4 to a few recombination complexes per chromosome occurs^14,31^, but mature crossover-specific recombination foci (marked by CDK2, HEI10 and MutLγ) and/or crossovers do not form as observed in *Mlh3*^*−/−*^ mice^6,15,31^. Hence, to test PRR19 involvement in crossover differentiation, we examined DSB repair kinetics in *Prr19*^*−/−*^ spermatocytes by detecting markers of unrepaired DSBs.

**Fig. 5:**
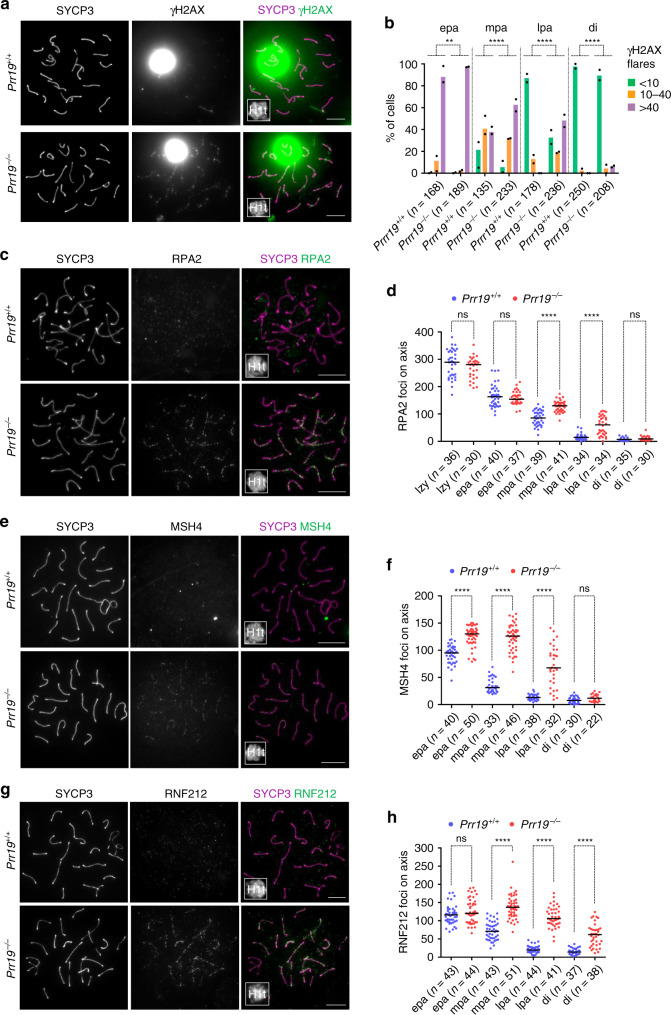
DSB repair is delayed in PRR19-deficient cells. **a**, **c**, **e**, **g** Indicated proteins were detected by immunofluorescence in nuclear surface-spread spermatocytes; late pachytene cells are shown. Miniaturised H1t signal of the corresponding cell is shown in the bottom left corner of overlay images. Saturated γH2AX signal (**a**) corresponds to the silenced chromatin of sex chromosomes, which is a chromatin compartment where histone γH2AX hyperaccumulates in pachytene and diplotene stages. Bars, 10 µm. **b** Quantification of γH2AX flare numbers in early (epa), mid (mpa), late pachytene (lpa) and diplotene (di) spermatocytes. Three categories were distinguished, (green) spermatocytes with less than 10 flares, (orange) between 10 and 40 flares and (lilac) more than 40 flares of γH2AX on autosomes. Graph shows datapoints and weighted averages of percentages of spermatocytes in the three categories, *n* = numbers of analysed cells from two animals. An analysis of deviance using the likelihood-ratio test based on the chi-squared distribution was used to calculate if loss of PRR19 significantly alters the proportions of cells with distinct numbers of γH2AX flares in epa, *P* = 0.00129, mpa, *P* = 2.48E-07, lpa, *P* = 2.20E-16, di, *P* = 1.23E-05. **d**, **f**, **h** Quantification of (**d**) RPA, (**f**) MSH4 and (**h**) RNF212 axis-associated focus numbers in late zygotene (lzy, RPA only), early (epa), mid (mpa), late pachytene (lpa) and diplotene (di) spermatocytes. *n* = numbers of analysed cells from two animals. Median focus numbers (bars) were compared with Mann–Whitney *U* test, and are as follows in wild-type and *Prr19*^*−/−*^: (**d**, RPA) lzy, 289.5 and 280.5 (*P* = 0.1492), epa, 163 and 154 (*P* = 0.6426), mpa, 85.5 and 130 (*P* = 7.12E-10), lpa, 14.5 and 60.5 (*P* = 2.32E-07), di, 7 and 9 (*P* = 0.1148), (**f**, MSH4) epa, 95 and 130 (*P* = 3.13E-11), mpa, 31 and 126 (*P* = 4.99E-14), lpa, 13 and 67.5 (*P* = 2.21E-10), di, 7.5 and 11.5 (*P* = 0.05838), (**h**, RNF212) epa, 116 and 120.5 (*P* = 0.1783), mpa, 71 and 137 (*P* = 2.18E-14), lpa, 19.5 and 106 (*P* = 2.26E-15), di, 14 and 62 (*P* = 6.12E-11), respectively. **b**, **d**, **f**, **h** ns, **, *** and **** indicate no significance (*P* > 0.05), 0.01 > *P* > 0.001, 0.001 > *P* > 0.0001 and *P* < 0.0001, respectively. Source data are provided as a Source Data file.

One of the markers, phosphorylated histone H2AX (γH2AX), accumulates on chromatin surrounding unrepaired DSBs and, specifically in meiosis, also on unsynapsed chromosomes (reviewed in refs. ^33,35^). Heterologous sex chromosomes are largely unsynapsed, hence they form γH2AX-rich sex bodies in wild-type pachytene- and diplotene-stage spermatocytes^33,35^. Low amounts of γH2AX are also present in chromatin flares on synapsed autosomes in early pachytene indicative of unrepaired DSBs^36^, but most DSBs are repaired causing loss of autosomal γH2AX flares in mid pachytene and beyond. While sex body formation was unchanged, autosomal γH2AX flares persisted longer in *Prr19*^*−/*−^ spermatocytes as compared to wild-type (Fig. 5a, b). Kinetics of additional markers of recombination intermediates also suggested DSB repair delay in *Prr19*^−*/*−^ spermatocytes. The turnover of most examined recombination proteins (RAD51, RPA and MSH4) was delayed, with foci persisting through mid and late pachytene in *Prr19*^*−/−*^ spermatocytes (Fig. 5c–f; Supplementary Fig. 8c–e). Only DMC1 foci did not persist (Supplementary Fig. 8a, b), which was reminiscent of *Hei10*^*mei4/mei4*^ phenotypes^18^ and was consistent with a hypothesis that DMC1 has diminished role in recombination beyond mid pachytene^18,37^. The persistence of MSH4 foci in *Prr19*^−*/−*^ spermatocytes was particularly significant because it suggested failed crossover differentiation due to an abnormal stabilisation of strand-invasion intermediates^14–16,18^.

The RNF212 SUMO-ligase is thought to stabilise recombination intermediates during pachytene^14,18^. Accordingly, abnormal persistence of recombination markers is accompanied by, and likely depends on, abnormal persistence of RNF212 on chromosomes in HEI10- and CNTD1*-*deficient meiocytes^15,16,18^. RNF212 foci abnormally persisted in *Prr19*^*−/−*^ spermatocytes during pachytene (Fig. 5g, h; Supplementary Fig. 8f). Thus, like HEI10 and CNTD1, PRR19 is critical for the correct regulation of RNF212, and the associated paring down of MutSγ-stabilised recombination intermediates during class I crossovers differentiation.

### Meiotic defects in *Prr19*^*−/*−^ oocytes

**Fig. 6:**
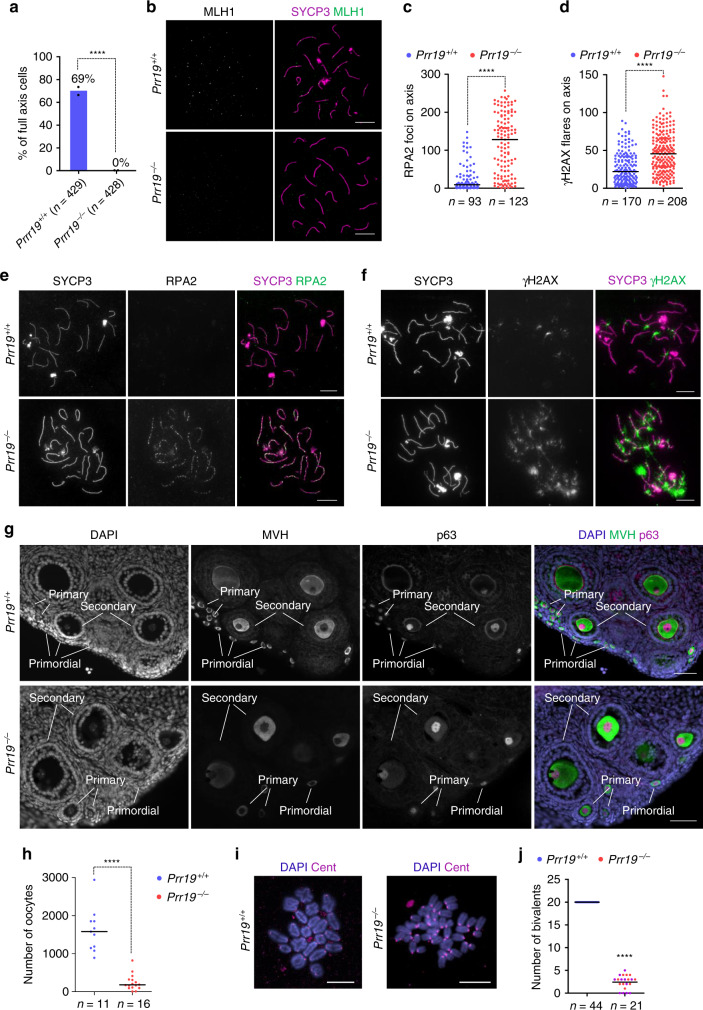
Crossover formation and DSB repair are defective in Prr19^−/−^ oocytes. **a** Diagram shows percentages of oocytes with MLH1 foci in pools of oocytes that had fully formed axes in 18 dpc foetuses, datapoints and weighted averages of percentages are shown. *n* = numbers of analysed cells from two animals. Fisher’s exact test, *P* < 2.2E-16 (****). **b**, **e**, **f** Images show immunofluorescence of indicated proteins in nuclear spreads of (**b**) mid, (**e**) late pachytene or (**f**) early diplotene oocytes from (**b**) 18 dpc foetuses or (**e**, **f**) newborn mice. Bars, 10 µm. **c**, **d** Quantification of RPA focus numbers (**c**) and γH2AX flare numbers (**d**) in oocytes with fully formed axes from newborn mice.* n* = numbers of analysed cells from two mice; Mann–Whitney *U* test compared medians (bars) which are as follows in wild-type and *Prr19*^*−/−*^: RPA, 9 and 128 (*P* < 2.2E-16), γH2AX, 22 and 45.5 (*P* = 2.83E-14). **g** Oocytes were immunolabelled on ovary sections with MVH (cytoplasmic) and p63 (nuclear). DNA was labelled by DAPI. Primordial, primary and secondary follicles are indicated. Bars, 50 µm. **h** Quantification of oocyte numbers in ovary sections from 6- to 7-weeks-old females of indicated genotypes. Sums of oocyte numbers from every 6th sections of both ovaries of each mouse are shown. *n* = numbers of analysed animals; Mann–Whitney *U* test compared median numbers of oocytes (bars, wild-type, 1579 and *Prr19*^−/−^, 180.5), *P* = 1.53E-07 (****). **i**, **j** Oocytes were matured in vitro to metaphase I stage. **i** DNA was labelled with DAPI and centromeres (Cent) were detected by immunofluorescence on spreads of oocytes. Bars, 20 µm. **j** Quantification of bivalent numbers in spreads from matured oocytes. Numbers of analysed cells (*n*) and means (bars) are indicated. Note that mean bivalent number (2.4) is an underestimate in *Prr19*^*−/*−^ oocytes, as we were able to identify full chromosome sets only in 10 out of 21 analysed *Prr19*^*−/−*^ oocytes (red circles) due to a disorganised metaphase plate; more than half of the full chromosome set was detected in each of the remaining 11 oocytes (lilac circles). Statistics by one-tailed one-sample *t* test, *P* < 2.2E-16 (****). Source data are provided as a Source Data file.

To investigate if PRR19 functioned also in females, we examined *Prr19*^*−/−*^ oocytes. Whereas *Prr19*^−*/*−^ oocytes were competent for SC formation, they did not form MutLγ-marked crossover precursors at a developmental stage where most wild-type oocytes did (Fig. 6a, b), implicating PRR19 in crossover formation in females. DSB markers, such as RPA and γH2AX, abnormally persisted in late pachytene and diplotene oocytes (Fig. 6c–f), suggesting that, as in spermatocytes, DSB repair is delayed in *Prr19*^*−/−*^ oocytes. Persistent DSBs trigger oocyte apoptosis perinatally in mice^38^, and accordingly, oocyte pools were diminished in young adults of *Prr19*^−*/*−^ mice (Fig. 6g, h). Residual *Prr19*^−*/−*^ oocytes retained competence to mature to an abnormal metaphase I with most homologs present as unconnected univalents. This contrasted wild-type oocytes, where homologs were combined in bivalents by chiasmata (Fig. 6i, j). Thus, PRR19 has similar roles in recombination in males and females.

### PRR19 and CNTD1 collaborate to promote crossover formation

Loss of PRR19, HEI10 or CNTD1 causes similar defects in meiotic recombination, indicating potential PRR19 involvement in HEI10- and CNTD1-mediated functions in crossover formation.

HEI10-deficiency impairs accumulation of ubiquitin and 26S proteasomes on synapsed chromosome axes in pachytene^18^. Hence, it was speculated that HEI10-mediated ubiquitination and a resultant proteasome-mediated degradation of as yet unidentified recombination proteins promotes the paring down of RNF212/MutSγ-associated strand-invasion intermediates and subsequent maturation of the persisting intermediates into crossovers. Contrasting the phenotypes of HEI10-deficient spermatocytes, ubiquitin levels were not affected significantly and proteasome levels were only moderately reduced below wild-type levels along chromosome axes in mid/late pachytene *Prr19*^−*/*−^ spermatocytes (Supplementary Fig. 9a–d). This might indicate reduced proteasome activity in *Prr19*^−*/*−^ spermatocytes. However, the biological significance of this observation is unclear because, all else unchanged, a significant reduction of proteasome activity is expected to cause increased ubiquitin levels along meiotic chromosomes^18^.

Differences between phenotypes of HEI10- and PRR19-deficient spermatocytes extended to the timing of their apoptosis. Whereas most *Prr19*^−*/*−^ spermatocytes were eliminated during the late prophase, most HEI10-deficient meiocytes survived beyond pachytene (Supplementary Fig. 10a, b) and arrested in metaphase I^17^. Thus, PRR19 and HEI10 seem to function in distinct processes, despite their similar overall contributions to crossover differentiation and maturation.

**Fig. 7:**
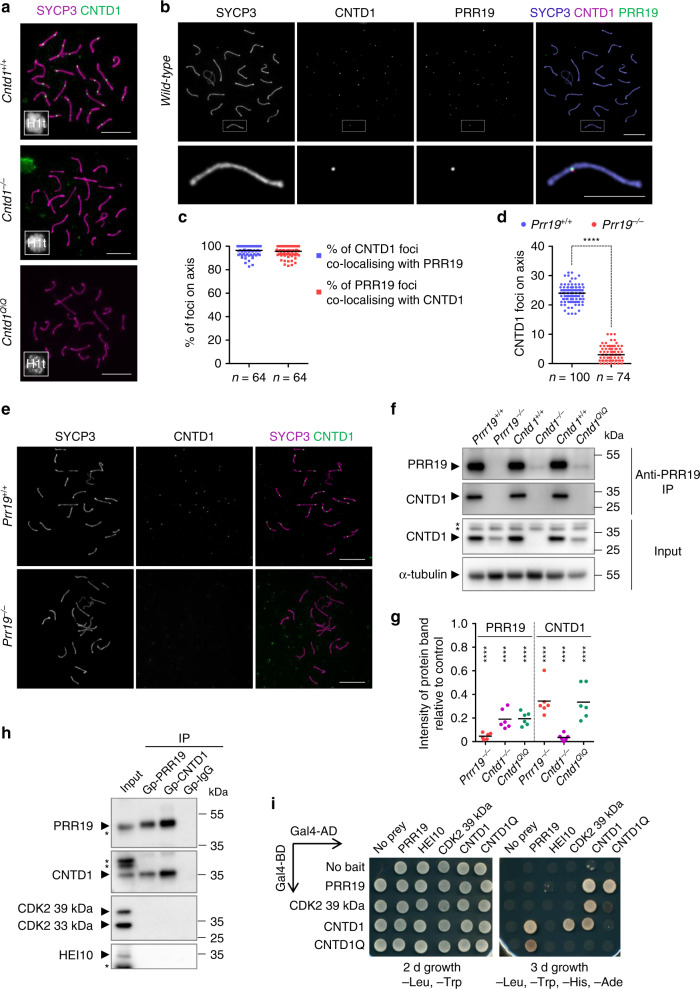
PRR19 forms a complex with cyclin-like CNTD1. **a**, **b**, **e** Immunofluorescence staining of surface-spread mid/late pachytene spermatocytes. **b** Bottom panels show enlarged insets. **a**, **b**, **e** Bars, 10 µm, or (bottom panel, **b**) 5 µm. **c** Quantification of co-localisation between CNTD1 and PRR19 foci on the chromosome axis in mid/late pachytene wild-type spermatocytes, medians (bars) are 96.4% (blue data set) and 95.7% (red data set). **d** Quantification of axis-associated CNTD1 focus numbers in wild-type and *Prr19*^−*/−*^ mid/late pachytene spermatocytes. Medians (bars) are 24 and 3 in *Prr19*^*+/+*^ and *Prr19*^*−/−*^, respectively. Mann–Whitney *U* test, *P* < 2.2E-16 (****). **c**, **d** *n* = numbers of analysed cells from two animals. **f**, **h** Immunoblots of immunoprecipitation experiments from testis extracts of (**f**) 13-days-old or (**h**) adult mice. Arrowheads mark bands of indicated proteins. Asterisks mark unspecific protein bands. Molecular weights are indicated. **f**, upper panel: PRR19 was detected in immunoprecipitates (IP) of guinea pig anti-PRR19 antibodies to assess PRR19 levels without interference from unspecific immunoblot signals that are present in total extracts. **f**, two middle panels: CNTD1 in anti-PRR19 IPs and corresponding input samples. **f**, bottom panel, anti-α-tubulin used as loading control. **h** Images show input for immunoprecipitation, immunoprecipitates (IP) of guinea pig anti-PRR19 (Gp-PRR19), anti-CNTD1 (Gp-CNTD1) and non-specific (Gp–IgG) antibodies. **g** Immunoblot signals of PRR19 (from anti-PRR19 IPs) or CNTD1 (from testis extracts) in the indicated genotypes were normalised against corresponding immunoblot signals of wild-type littermate controls. Two-tailed one-sample *t* test calculated significance of difference between the wild-type value of one and means of six experiments (bars) for PRR19, 0.05 (*P* = 3.63E-09), 0.19 (*P* = 2.17E-06) and 0.19 (*P* = 3.52E-07), and CNTD1, 0.34 (*P* = 6.82E-05), 0.03 (*P* = 5.42E-09) and 0.33 (*P* = 9.27E-05), in *Prr19*^*−/−*^, *Cntd1*^*−/*−^ and *Cntd1*^*Q/Q*^ samples, respectively. **** indicate *P* < 0.0001. **i** Yeast two-hybrid interaction assays between indicated proteins. CNTD1Q refers to the protein product of *Cntd1*^*Q*^ allele (see Supplementary Fig. 3). Yeast cultures are shown after 2 or 3 days of growth on dropout plates. For negative control, proteins of interest were tested in transformations where either the Gal4-binding domain (Gal4-BD) or the Gal4-activation domain (Gal4-AD) vectors were empty. Source data are provided as a Source Data file.

Most CNTD1-deficient spermatocytes were eliminated at or before diplotene, closely resembling *Prr19*^*−/−*^, but contrasting HEI10-deficient spermatocytes (Supplementary Fig. 10a, b). Given that all tested phenotypes of CNTD1- and PRR19-deficient spermatocytes were very similar, we speculated that CNTD1 and PRR19 function together. Although the *C. elegans* CNTD1 ortholog, COSA-1^39^, assembles into crossover-specific foci, previous attempts to localise CNTD1 to mouse meiotic chromosomes failed^16^. However, our anti-CNTD1 antibodies detected chromosome-associated foci whose specificity was confirmed by a lack of signal in CNTD1-deficient spermatocytes (Fig. 7a). The temporal and spatial patterns of CNTD1 and PRR19 foci were very similar, and chromosome-associated foci of CNTD1 and PRR19 co-localised in the mid/late pachytene (Fig. 7b, c). CNTD1 also co-localised with interstitial CDK2 foci that mark crossover sites (Supplementary Fig. 11a, b). Furthermore, CNTD1 foci were absent in *Prr19*^−*/−*^ mutant spermatocytes (Fig. 7d, e). Thus, both CNTD1 and PRR19 mark crossover precursors in mice. Given that CNTD1 and PRR19 were interdependent for focus formation, we tested whether the stability of CNTD1 or PRR19 was altered in *Prr19*^−*/−*^, *Cntd1*^−*/−*^ or *Cntd1*^*Q/Q*^ mutant mice. Altered testis cellularity due to apoptosis distorts protein level measurements in testes of adult *Prr19*^−*/*−^, *Cntd1*^−*/*−^ and *Cntd1*^*Q/Q*^ mutants. To avoid this complication, we assessed CNTD1 and PRR19 levels in 13-days-old mice. At this age, most spermatocytes of the first spermatogenic wave have not reached mid/late pachytene yet, hence testis cellularities are similar in all lines. CNTD1 and PRR19 levels were strongly reduced in *Prr19*^−*/−*^, *Cntd1*^−*/−*^ and *Cntd1*^*Q/Q*^ mutant testes (Fig. 7f, g), suggesting mutual dependence between these proteins.

Complementing these results, co-immunoprecipitation (co-IP) experiments suggested that PRR19 and CNTD1 associate with one another in wild-type testis (Fig. 7f, h). CNTD1 also interacted with itself and PRR19 in yeast two-hybrid (Y2H) assays (Fig. 7i; Supplementary Figs. 12, 13). The PRR19–CNTD1 interaction involved the third conserved domain and the surrounding regions of PRR19 and the *C*-terminal portion of CNTD1 (Supplementary Figs. 12, 13). Together, these observations suggest that PRR19 and CNTD1 function as a complex to effect crossing over.

In contrast, neither co-IP nor Y2H assays indicated physical interactions between HEI10 and CNTD1 or PRR19 (Fig. 7h, i), consistent with a hypothesis that the PRR19–CNTD1 complex and HEI10 have separate functions in crossover differentiation.

The cyclin-like CNTD1 was previously speculated to control crossover formation by modulating the activity of CDK2 kinase^16^. Co-localisation of CDK2 and CNTD1 at crossover-precursor sites (Supplementary Fig. 11a, b) is consistent with this hypothesis. Hence, we tested whether CDK2 can interact with PRR19 or CNTD1. While we did not detect CDK2 in immunoprecipitates of PRR19 or CNTD1 (Fig. 7h), CDK2 interacted with CNTD1 in Y2H assays (Fig. 7i). This discrepancy may be reconciled by the considerations that CDK2-CNTD1 complexes exist in vivo, but are transient, low abundance, and/or form only within the insoluble compartment of the SC. Given the paucity and transience of crossover-specific recombination complexes, these considerations seem reasonable. Importantly, the specificity of CNTD1 interactions argues against the possibility that CNTD1 and CDK2 interaction is an artefact of the heterologous Y2H system. We found that CNTD1 did not interact with other major cell-cycle CDKs (Supplementary Fig. 14), suggesting a specific interaction with CDK2. Among the 33-kDa and the 39-kDa isoforms of CDK2, CNTD1 preferentially interacted with the 39-kDa isoform, which has been implicated in meiosis-specific CDK2 functions previously^40–42^. Further, the CDK2-CNTD1 interaction did not require catalytic activity of CDK2 (Supplementary Fig. 14), but required the C-(PSTAIRE) helix of CDK2 (Supplementary Fig. 14), which forms part of the interface between CDK2 and its activator cyclins, cyclin E1 and A2^43,44^. Cyclins contain tandem duplications of the so-called cyclin box fold^45^. Most residues that are conserved between the CDK2-interacting interfaces of cyclin E1 and A2 are contributed by the first cyclin box, an *N*-terminally adjacent helix and the junction of the first and second cyclin boxes^43–45^. The CNTD1–CDK2 interaction requires the first but not the second predicted cyclin box of CNTD1 suggesting potential similarity to CDK2 interactions with cyclin E1/A2. Importantly, the *Cntd1*^*Q*^ mutation, which alters the first cyclin box of CNTD1, completely disrupted CNTD1 functions in vivo (see Supplementary Fig. 4; Fig. 7f, g). The *Cntd1*^*Q*^ mutation also severely diminished the CNTD1–CNTD1 and the CNTD1–CDK2 interactions but had only a minor impact on the PRR19–CNTD1 interaction in Y2H (Fig. 7i). Whereas interpretations of these observations are complicated by the low levels of CNTD1^Q^ in vivo, the effects of the *Cntd1*^*Q*^ mutation are consistent with the inference that the CNTD1–CDK2 interaction is biologically relevant for PRR19–CNTD1 functions.

## Discussion

Recombination intermediates sustain or lose RNF212/MutSγ beyond early pachytene as they commit to repair with or without crossing over formation, respectively. Thus, differentiation of crossovers from non-crossovers manifests by the gradual restriction of RNF212/MutSγ to one or a few crossover-specific recombination sites per chromosome in early-to-mid pachytene. Prior publications showed that crossover differentiation in mouse critically depends on an ubiquitin ligase, HEI10, and a cyclin-like protein, CNTD1.

**Fig. 8:**
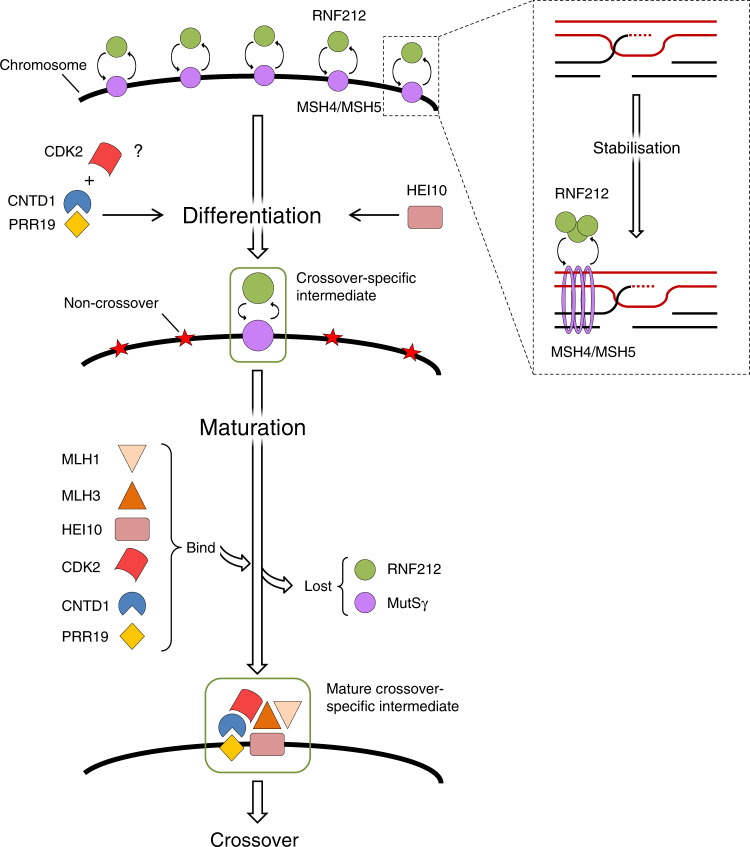
Summary and model of PRR19–CNTD1 functions in crossover formation. Block arrows represent progression in recombination, line arrows represent promotion. Inset shows recombination intermediates, where red and black lines represent the single-DNA strands of homologous DNA duplexes, and the dotted red line marks repair DNA synthesis. Whereas four chromatids are present at this stage, only two are shown for the sake of simplicity. A positive feedback between MutSγ (MSH4/MSH5 sliding clamp complex) and RNF212 stabilises DNA-strand-invasion intermediates that serve as a common precursor of crossovers and non-crossovers at the start of pachytene (see inset). Left, main scheme: The differentiation of crossover- and non-crossover-committed recombination intermediates takes place in the early–mid pachytene, and manifests by the restriction of MutSγ and RNF212 to one or two strand-exchange intermediates on each chromosome. Intermediates that retain MutSγ and RNF212 commit to crossover formation, the rest of the intermediates turn into non-crossovers (red stars) during the early–mid pachytene. Crossover differentiation and the attendant paring down of RNF212/MutSγ-associated recombination complexes are enabled by a competition between strand-invasion intermediates for MutSγ and RNF212 and by the restriction of RNF212/MutSγ accumulation and activity by the PRR19–CNTD1 complex and HEI10. PRR19–CNTD1 may act in partnership with CDK2 to control the stability of RNF212/MutSγ-associated recombination complexes. Crossover differentiation is followed by the maturation of RNF212/MutSγ-associated recombination complexes in mid-late pachytene. This involves the replacement of RNF212/MutSγ with cytologically detectable amounts of MLH1, MLH3, CDK2, PRR19, CNTD1 and HEI10 in crossover-committed recombination complexes. Whereas the MLH1-MLH3 complex is thought to resolve DNA-exchange intermediates into crossovers, the roles of HEI10, CDK2, PRR19 and CNTD1 are not known in crossover maturation. They may enable crossover maturation only by promoting crossover differentiation in early–mid pachytene. Alternatively, as suggested by their localisation, they may have additional roles in the resolution of crossover-committed recombination intermediates into crossovers in mid-late pachytene.

We identified PRR19 as a new crossover differentiation factor in mouse meiosis (for a model of PRR19 function see Fig. 8). Like HEI10 and CNTD1, PRR19 is required for the paring down of RNF212/MutSγ-associated recombination complexes and class I crossover formation. Our data also provide insight into the functional relationships between PRR19, CNTD1 and HEI10. *Prr19*^*−/−*^ and *Cntd1*^*−/−*^ mice have indistinguishable phenotypes. Furthermore, PRR19 and CNTD1 co-localise in crossover-specific recombination complexes on pachytene chromosomes (Fig. 7b, c), form complexes in testis extracts (Fig. 7f, h), and physically interact in Y2H (Fig. 7i; Supplementary Figs. 12, 13). Consistent with them forming a complex, PRR19 and CNTD1 appear to be interdependent for protein stability (Fig. 7f, g). Based on these observations, we propose that PRR19 promotes crossing over as part of a complex with CNTD1. In contrast, although HEI10 also localises to crossover-specific recombination complexes, we did not detect HEI10 in PRR19 or CNTD1 immunoprecipitates (Fig. 7h), nor did HEI10 physically interact with PRR19 or CNTD1 in Y2H assays (Fig. 7i). We also noted marked differences between the meiotic phenotypes of *Prr19*^−*/*−^ and HEI10-deficient mice. Unlike HEI10, PRR19 is not required for bulk ubiquitin accumulation along chromosome axes (Supplementary Fig. 9). This suggests that HEI10 activity does not require PRR19 per se. Whereas chromosome-associated proteasome levels were mildly reduced in *Prr19* mutants, the levels of ubiquitinated chromosomal proteins were not significantly increased, suggesting normal turnover of most ubiquitinated chromosomal proteins in the absence of PRR19. We infer that proteasome activity does not require PRR19 per se. Nonetheless, PRR19 may act as a specificity factor that enables ubiquitination and/or degradation of a critical subset of HEI10 substrates without affecting bulk ubiquitination or proteasome activity. Alternatively, HEI10 and PRR19 might destabilise RNF212/MutSγ at prospective non-crossover sites via independent, but non-redundant mechanisms. Irrespective of these alternatives, the above observations suggest separable functions for PRR19–CNTD1 complex and HEI10 in crossover formation.

Whereas the protein domains of PRR19 have unknown functions, CNTD1 contains a tandem duplication of the conserved cyclin box fold (Supplementary Fig. 13)^39^. Cyclin box-containing proteins function both as complexes with cyclin-dependent kinases and independently in diverse processes including cell-cycle regulation, transcription, proteolytic degradation and DNA damage repair^46^.

Notably, the cyclin-dependent kinase CDK2 co-localises with PRR19 and CNTD1 in crossover-specific recombination complexes, and CNTD1 interacts with CDK2 in the Y2H system (Fig. 7; Supplementary Figs. 1, 11, 13, 14). Although we could not detect stable complexes between CDK2 and PRR19 or CNTD1 in testis extracts (Fig. 7h), steady-state levels of such complexes are expected to be extremely low. Hence, we speculate that PRR19–CNTD1 collaborate with CDK2 in crossover formation. Interestingly, a small deletion in the first cyclin box of CNTD1 (Cntd1^Q^ mutation) abrogated CNTD1–CDK2 but not PRR19–CNTD1 Y2H interactions, and the same deletion caused crossover differentiation failure in vivo (Fig. 7i; Supplementary Figs. 3, 4). Thus, PRR19–CNTD1 function may depend on an interaction between CNTD1 and CDK2 in meiosis. However, both PRR19 and CNTD1^Q^ protein levels are reduced in *Cntd1*^*Q/Q*^ testes, which complicates the interpretation of *Cntd1*^*Q/Q*^ phenotypes (Fig. 7f, g). The *Cntd1*^*Q*^ mutation may disrupt CNTD1 folding, leading to a loss of PRR19–CNTD1 function and stability in vivo. Alternatively, the *Cntd1*^*Q*^ mutation may impair primarily the CNTD1–CDK2 interaction as suggested by our Y2H data. In this scenario, diminished PRR19–CNTD1 levels, complex formation and function would be the consequences of impaired CNTD1–CDK2 interaction in vivo. The latter scenario invokes a model in which the PRR19–CNTD1 complex regulates and/or targets CDK2 activity to implement crossover formation via phosphorylation of as yet unknown targets. Relevant to this possibility, conclusions from domain mapping of CNTD1 interactions are consistent with a putative physical interaction between CDK2 and the PRR19–CNTD1 complex in vivo. Specifically, the first and second cyclin boxes of CNTD1 are differentially important for interaction with CDK2 and PRR19, respectively (Supplementary Fig. 13). Thus, distinct molecular features of CNTD1 mediate interactions with PRR19 and CDK2, which may permit the PRR19–CNTD1 complex to bind and regulate CDK2 in the meiosis.

Persistence of RNF212/MutSγ-associated recombination complexes indicates that PRR19-, CNTD1- and HEI10-deficient meiocytes fail to mature recombination intermediates as non-crossovers, and instead appear to commit them to crossover formation during early–mid pachytene. Curiously, RNF212/MutSγ-rich recombination intermediates mature into class I crossovers in wild-type but not in HEI10-, CNTD1- or PRR19-deficient cells. It follows that PRR19, CNTD1 and HEI10 are not only required for the differentiation of non-crossover and crossover pathways but also for the maturation of RNF212/MutSγ-associated recombination intermediates into crossovers. PRR19, CNTD1 and HEI10 may effect crossover maturation only indirectly, because crossover differentiation and maturation might be mechanistically linked. Specifically, crossover maturation may require that high levels of pro-crossover proteins (e.g., MutLγ) are amassed in crossover-competent recombination intermediates^22^. If factors such as MLH1 and MLH3 are limiting, such accumulation may necessitate that the number of crossover-competent sites is reduced to a few per chromosome in the mid pachytene by crossover differentiation. Alternatively, the crossover differentiation-related functions of PRR19, CNTD1 and HEI10 may be separable from their roles in crossover maturation. Consistent with the latter hypothesis, all three proteins accumulate in mature crossover-specific recombination complexes, which are rich in MutLγ. Further, loss of MutLγ component MLH3 disrupts the concentration of PRR19 (Fig. 1f, g) and HEI10^15^ into crossover-specific recombination complexes. Thus, PRR19, CNTD1 and HEI10 might act both upstream of MLH3 in crossover differentiation and downstream of MLH3 in crossover maturation. PRR19, CNTD1 and HEI10 may enact similar molecular functions in both of these roles. In particular, they may destabilise recombination proteins/complexes to promote the repair of MutLγ-poor and MutLγ-rich recombination intermediates as non-crossovers and crossovers in early–mid and late pachytene, respectively.

PRR19/CNTD1/HEI10-deficiency causes a spermatogenic block which is distinct from the so-called stage IV or mid pachytene arrest that characterises a broad range of recombination-defective mouse strains^32–34^. Recombination defects are thought to cause a spermatogenic block at the onset of mid pachytene in stage IV of the seminiferous epithelial cycle via two genetically separable mechanisms^35,47^. One of these is activated by a deficiency in the formation of the sex body, the transcriptionally silenced chromatin domain that encompasses the unsynapsed non-homologous regions of the X and Y chromosomes. Sex bodies form in wild-type spermatocytes by the silencing of unsynapsed chromosomes^35^, which depends on HORMAD1/HORMAD2-mediated enrichment of ATR activity in unsynapsed chromatin^48–52^. Recombination defects that lead to autosomal asynapsis cause a redistribution of HORMAD1-HORMAD2-ATR from sex chromosomes to asynaptic autosomes^33,35,53,54^. This diminishes the silencing of sex chromosomes^53^, which permits expression of genes that are deleterious for mid pachytene spermatocytes^33,55^.

Recombination defects that impair DSB repair are also thought to arrest spermatocytes in early pachytene via a mechanism that is independent of sex body formation but depends on a MRE11-ATM-CHK2-P53-Tap63-mediated checkpoint mechanism^47,56^. Curiously, abnormal persistence of unrepaired DSBs does not prevent progression beyond early pachytene in PRR19/CNTD1/HEI10-deficient spermatocytes (this study and refs. ^16,17^), which form sex bodies proficiently. While most HEI10-deficient spermatocytes progress to metaphase I^17^, where chiasma-deficiency is thought to arrest spermatocytes (reviewed in^33^), *Prr19*^*−/−*^ and *Cntd1*^*−/−*^ spermatocytes undergo apoptosis before diplotene (Supplementary Fig. 10); in particular, apoptosis peaks in late pachytene *Prr19*^*−/−*^ mutant spermatocytes (Fig. 3d; Supplementary Table 1).

These observations may indicate that, without a sex body-deficiency, unrepaired DSBs do not cause efficient elimination or indefinite arrest of spermatocytes before mid pachytene. Alternatively, the nature and quantity of unresolved recombination intermediates and resultant DNA damage signals may differ in meiosis-defective mouse strains that activate the stage IV checkpoint as opposed to PRR19/CNTD1/HEI10-deficient spermatocytes that do not. Finally, deficiency in PRR19/CNTD1/HEI10 may disrupt the MRE11-ATM-CHK2-P53-Tap63-mediated mechanism that is thought to block progression of DSB repair-defective spermatocytes beyond early pachytene. Regardless, our observations suggest that there is a checkpoint mechanism that triggers apoptosis in late pachytene-stage spermatocytes in response to unrepaired DSBs. Such a checkpoint is also consistent with the timing of apoptosis in diverse meiotic mutant mouse lines that feature delayed DSB repair without severe disruption of SC and sex body formation^3,6,27,57–59^. The genetic requirements for this late pachytene checkpoint are not known, but the observation that most HEI10-deficient spermatocytes survive until metaphase I, despite persistent unrepaired DSBs, suggests that HEI10 is involved.

## Methods

### Animal experiments

Gonads were collected from mice after euthanasia. Most cytological experiments of spermatocytes were carried out on samples collected from adult mice, unless indicated otherwise. *Prr19, Cntd1* and *Mlh1* mutant mice were used and maintained in accordance with the German Animal Welfare legislation (Tierschutzgesetz). The mice were kept in the barrier facility in individually ventilated cages at 22–24 °C and 50–55% air humidity with 14-h light/10-h dark cycle. The feed was a rat–mouse standard diet in the form of pellets. The stocking density in the used cage type IIL was maximum five mice. Hygiene monitoring was carried out according to FELASA guidelines. All procedures pertaining to animal experiments were approved by the Governmental IACUC (Landesdirektion Sachsen) and overseen by the animal ethics committee of the Technische Universität Dresden. The licence numbers concerned with the present experiments with animals are DD24-5131/287/1, TV A 8/2017 and TVV 73/2017. *Spo11*, *Sycp1, Rnf212*, *Mlh3* and *Hei10* mutant mice were maintained and used for experimentation according to the guidelines of the Institutional Animal Care and Use Committees of the University of California, Davis.

### Generation and genotyping of *Prr19*- and *Cntd1*-mutant mice

*Prr19 and* *Cntd1*-mutant lines were generated using CRIPSR/Cas9 genome editing^60^, targeting exon 2 of *Prr19* gene or exon 1 of *Cntd1* gene (see Fig. 2a and Supplementary Figs. 5a, 3a for targeting strategies). The guide RNAs (gRNAs) were designed using the online platform at http://crispr.mit.edu/. A mixture of gRNA(s), Cas9 mRNA and single-stranded DNA oligos (in case of *Cntd1* targeting) was injected into pronucleus/cytoplasm of fertilised oocytes. The oocytes were subsequently transferred into pseudopregnant recipients. Injections and embryo transfer were performed by Transgenic Core Facility of MPI-CBG (Dresden, Germany). For *Prr19* targeting, two gRNAs, GGAGGATCCTGACGGGCCAA and GGATCCATGTGTGATCCTCC (5 ng/μl each), and Cas9-D10A (Cas9 nickase) mRNA (20 ng/μl) were used. Out of 401 injected embryos, 339 were transferred into females and 47 were born. In total, 34 of the 47 born pups had alterations in the targeted genomic locus. Two mice that were heterozygote for predicted frameshift causing alleles (Mut1 and Mut2, Fig. 2a; Supplementary Fig. 5a) were bred with CD1 or C57BL/6JCrl wild-type mice to establish mouse lines. For *Cntd1* targeting, a mixture of gRNA TTCAGGGAGACCCGGATCGT (12.5 ng/µl), Cas9 nuclease mRNA (50 ng/µl) and two single-stranded DNA oligos, AAACCTGACTCCCCACTCTGCATCCACACCTACTATACGCGTCTCCCTGA and CCTGACTCCCCACTCTGCATCCACACCCACTATACGCGTTTCCCTGAAGC (1.25 ng/µl each), was used. Out of 146 injected embryos, 74 were transferred into females and 12 were born. In all, 11 of the 12 born pups had alterations in the targeted genomic locus. Two mice that were heterozygote for predicted frameshift causing allele (*Cntd1*^−*/−*^, Supplementary Fig. 3a) or allele causing splicing alterations (*Cntd1*^*Q/Q*^, Supplementary Fig. 3a) were bred with CD1 wild-type mice to establish mouse lines.

To construct the gRNAs for *Prr19* targeting, the gRNA expression vector DR274 (Addgene, #42250) was used^60^. Primers encoding gRNA sequence were annealed to form double-stranded DNA with overhangs for ligation into DR274 vector that was linearised with Bsal restriction enzyme (NEB). PCR product was amplified from the resulting plasmid with AAATGGTCAGTATTGAGCCTCAG and AAAAGCACCGACTCGGTGCCAC primers and used as a template for in vitro transcription with MEGAscript™ T7 Transcription Kit (Ambion). For *Cntd1* targeting, the template for in vitro transcription was prepared by template-free PCR with two oligos: a universal reverse oligo encoding tracrRNA and an oligo, including gRNA sequence and T7 promoter. gRNAs were purified from in vitro transcription reactions using MEGAclear™ Transcription Clean-Up Kit (Ambion).

To prepare Cas9-D10A (Cas9 nickase) and Cas9 mRNAs for microinjection, we used the restriction enzyme PmeI to linearise plasmids pST1374-Cas9-D10A and MLM3613 (Addgene #42251)^60^. The linearised plasmids were used as templates to synthesise the 5′-capped and 3′-poly(A)-tailed mRNAs using the mMESSAGE mMACHINE T7 Ultra Kit (Thermo Fisher Scientific, AM1345) according to the manufacturer’s instructions. To create the pST1374-Cas9-D10A plasmid, we inserted the Cas9-D10A-NLS gene that is released from hCas9-D10A plasmid (Addgene, #41816) with enzymes XbaI and AgeI, into the pST1374 vector (Addgene, #13426) linearised with NheI and AgeI. The sticky ends generated by XbaI and NheI were compatible and could be re-ligated in the presence of T4 DNA ligase.

### Genotyping animals

Tail biopsies were used to generate genomic DNA by overnight protease K digestion at 55 °C in lysis buffer (200 mM NaCl, 100 mM Tris-HCl pH 8, 5 mM EDTA, 0.1% SDS). Following heat inactivation for 10 min at 95 °C, these genomic preparations were used for PCR.

The *Spo11*^*−/−*^, *Sycp1*^*−/−*^*, Rnf212*^*−/−*^, *Mlh3*^*−/−*^, *Hei10*^*mei4/mei4*^ and *Mlh1*^*Lisk/Lisk*^ mutant lines and genotyping primer sequences were described earlier (for the primer sequences refer to Supplementary Table 4)^3,6,14,17,29,30^.

*Prr19*-targeted F0 mice were genotyped by PCR amplification followed by agarose electrophoresis and DNA sequencing. A combination of two primers, GCACCACAAGGACTGGCTATTGTC and GAGTTGGTACTGCAACTCCTCCAG, was used to genotype *Prr19*^*Mut1*^ and *Prr19*^*Mut2*^ alleles. PCR product sizes were 732 bp for wild-type, 682 bp for *Prr19*^*Mut1*^ allele and 345 bp for *Prr19*^*Mut2*^ allele. *Cntd1*-targeted F0 mice were genotyped by PCR amplification followed by PAGE electrophoresis, FastDigest MluI (Thermo Scientific) restriction digestion (restriction site introduced by single-stranded DNA oligos to monitor homologous recombination efficiency) and DNA sequencing. A combination of two primers, CGAAGCGAGGGCAAACATAT and CATCTGTCAATTCCAGGCCAG, was used to genotype F0 animals and, subsequently, *Cntd1*^*Q*^ allele. PCR product sizes after MluI restriction digestion were 580 bp for wild-type and 286 bp + 295 bp for *Cntd1*^*Q*^ allele. After initial PCR amplification and DNA sequencing of *Cntd1*^*−*^ allele, the combination of three primers was used for genotyping: CGAAGCGAGGGCAAACATAT and CTGCATCCACACCCACGAT to amplify 310 bp wild-type allele; CGAAGCGAGGGCAAACATAT and CTGCATCCACACCCACGAA to amplify 310 bp *Cntd1*^*−*^ allele.

### Generation of antibodies

Antibodies were raised against PRR19 C-terminal fragment (183 amino acids between Thr183 and Tyr366 residues) and CNTD1 *C*-terminal fragment (124 amino acids between Cys126 and Thr249 residues). Coding sequences corresponding to these peptides were cloned into pDEST17 bacterial expression vector. Recombinant 6xHis-tagged proteins were expressed in *E. coli* strain BL21 tRNA and subsequently purified by Ni-Sepharose beads and SDS-PAGE (Amersham, GE Healthcare). Either elution fractions or homogenised SDS-PAGE gel fragments containing the purified proteins were used for immunisation of rabbits and guinea pigs. PRR19 or CNTD1 fragments coupled to NHS-activated Sepharose 4 Fast Flow beads (Amersham, GE Healthcare) were used to affinity purify polyclonal antibodies following standard procedures. Goat anti-RNF212 was raised against full-length mouse RNF212 protein. The specificity of anti-PRR19, anti-CNTD1 and anti-RNF212 antibodies was confirmed by: (1) immunoprecipitations and western blot analysis of protein extracts from testes of wild-type and *Prr19*^*−/−*^, *Cntd1*^*−/−*^ and *Rnf212*^*−/−*^ mice, respectively; (2) immunostaining of spermatocyte nuclear surface spreads from testes of wild-type and mutant mice.

### RNA isolation and RT-PCR

To test *Prr19* or *Cntd1* expression in testis, the total RNA was isolated from adult mouse testis tissue using the RNeasy Mini Kit (Qiagen). 0.5 µg of the total RNA was reverse transcribed using Superscript III (Invitrogen, 18080-044) and oligo dT (20) primers. In no RT controls, the reaction mixture contained water instead of reverse transcriptase (RT). RT-PCR of housekeeping 40S ribosomal protein S9 gene (Rps9) was used as control. PCR products were amplified from resulting cDNAs using following primers: for *Prr19* a pair of primers annealing in exon 1 (GAGGCTTGGGGGTCTCTAC) and exon 2 (CGGTGCTCCCGGCTCAAC) amplifying 332-bp long fragment from wild-type cDNA; for *Cntd1* a pair of primers annealing in exon 1 (ATGAATATGGAAGGACCCTTGAGG) and exon 2 (CTACAGCCTGGTAGCTCACC) amplifying 238-bp long fragment from wild-type cDNA.

### Cytoplasmic and nuclear fractionation for PRR19 detection

Testes of adult mice were detunicated and homogenised in PBS pH 7.5 supplemented with protease inhibitors and phosphatase inhibitors: 1 mM phenylmethylsulfonyl fluoride (PMSF); complete™ EDTA-free Protease Inhibitor Cocktail tablets (Roche, 11873580001); 0.5 mM sodium orthovanadate; phosphatase inhibitor cocktail 1 (Sigma, P2850) and phosphatase inhibitor cocktail 2 (Sigma, P5726) were used at concentrations recommended by the manufacturers. The resulting cell suspensions were spun for 10 min at 1000 *g* at 4 °C, and cells from the pellet were lysed in hypotonic buffer (40 mM Tris-HCl, pH 7.5, 5 mM KCl, 2 mM EDTA) supplemented with protease and phosphatase inhibitors and 10 mM spermidine (Sigma, S2626) to preserve nuclei. The resulting total cell lysate was spun at 1000 *g* at 4 °C for 10 min. Supernatant was collected as cytoplasmic fraction, while the nuclear pellet was further lysed for 1 h at 4 °C in lysis buffer (50 mM Tris-HCl pH 7.5, 150 mM NaCl, 1% Triton X-100, 1 mM MgCl_2_, 1 mM CaCl_2_) supplemented with protease and phosphatase inhibitors and benzonase (Merck Millipore) to digest DNA.

### PRR19 and CNTD1 immunoprecipitation

For the preparation of protein extracts from wild-type and mutant testes, testes were detunicated and homogenised in a lysis buffer (50 mM Tris-HCl pH 7.5, 150 mM NaCl, 0.5% Triton X-100) supplemented with protease and phosphatase inhibitors as described above. Testis homogenates were lysed for 60 min at 4 °C. For immunoprecipitation input samples, lysates were spun at 10,000 *g* for 5 or 10 min. Supernatants were diluted two times with 50 mM Tris-HCl pH 7.5, 150 mM NaCl, and used for immunoprecipitation with 1.5 µg (Fig. 7f) of guinea pig anti-PRR19 or 3 µg (Fig. 7h) of guinea pig anti-PRR19, guinea pig anti-CNTD1 or guinea pig IgG (NBP1-97036, Novus Biologicals) antibodies crosslinked to 1.5 mg of Dynabeads™ Protein A (Invitrogen) using 20 mM dimethyl suberimidate according to the standard protocols. After overnight incubation at 4 °C, beads were washed three times with 50 mM Tris-HCl pH 7.5, 150 mM NaCl, 0.1% Triton X-100. Immunoprecipitated material was eluted from the beads by incubating the beads in 100 µl Laemmli sample buffer for 10 min at 70 °C (Fig. 7h) or by incubating the beads in 40 µl Laemmli sample buffer containing 5% β-mercaptoethanol for 10 min at room temperature followed by brief vortexing (Fig. 7f).

### Total testis protein extract preparation

For the preparation of total protein extracts from testes of adult and juvenile wild-type and mutant mice, testis homogenates were lysed as described above for 30 min at 4 °C. Lysates were supplemented with 1 mM MgCl_2_, 1 mM CaCl_2_ and benzonase (Merck Millipore) to digest DNA and incubated for 30 min at 4 °C. Samples were then mixed with Laemmli sample buffer, boiled for 4 min at 98 °C and spun at 20,000 *g* for 10 min.

### Western blotting and protein detection

The proteins from total protein extracts, nuclear and cytoplasmic fractions, immunoprecipitation input samples and elutions were separated on 4–15% TBX-acrylamide gradient gels (Bio-Rad) and blotted onto the PVDF membrane (Sigma, P2938). Membranes were blocked for 1 h at room temperature using blocking solution: 5% skimmed milk in TBS pH 7.6 with 0.05% Tween 20 (TBS-T) and incubated overnight at 4 °C with the following primary antibodies: rabbit anti-PRR19 (Fig. 7f, this study, 1:1000), guinea pig anti-PRR19 (Figs. 1a, 2c, 7h, this study, 1:3000), mouse anti-CDK2 (sc-6248, Santa Cruz, 1:500), mouse anti-HEI10 (ab118999, Abcam, 1:1000), mouse anti-GAPDH (sc-32233, Santa Cruz, 1:1000) or mouse anti-α-tubulin (T6199, Sigma, 1:5000) diluted in TBS-T; rabbit anti-CNTD1 (this study, 1:3000) diluted in 2.5% skimmed milk in TBS-T; rabbit anti-histone H3 (ab18521, Abcam, 1:200,000) diluted in 2.5% BSA in TBS-T. Afterwards, goat anti-guinea pig, anti-rabbit, anti-rabbit light chain or anti-mouse horseradish peroxidase (HRP)-conjugated secondary antibodies (106-035-003, 111-035-003, 211-032-171 or 115-035-166, Jackson ImmunoResearch), diluted 1:10,000 in blocking solution, were applied for 1 h at room temperature. Detection of secondary antibodies was performed with Immobilon Western Chemiluminescent HRP Substrate (Millipore).

### Protein amount measurement on immunoblots

To compare protein levels of PRR19 and CNTD1 in testis protein extracts from 13-days-old mice, protein amounts were measured on immunoblot images. PRR19 levels were assessed in anti-PRR19 immunoprecipitates to avoid interference from non-specific bands that are detectable on anti-PRR19 immunoblots of total testis extracts. CNTD1 levels were assessed in immunoblots of IP input samples because there are no interfering non-specific bands in anti-CNTD1 immunoblots of total testis extracts. To calculate background-corrected intensities of protein bands from immunoblots (Fig. 7g), we measured background in the vicinity of each protein band in their corresponding lanes. To compare background-corrected intensities of PRR19 and CNTD1 bands between *Prr19*^−*/−*^, *Cntd1*^−*/−*^ or *Cntd1*^*Q/Q*^ and their respective control samples, we took potential differences in IP inputs (for PRR19 measurement) and gel loading (for CNTD1 measurement) into account. To calculate a normalisation factor, we used background-corrected intensities of α-tubulin bands in the immunoblots of input samples. Measurements were done using Adobe Photoshop CS5 software.

### Yeast two-hybrid assays

Pairwise interactions were tested in the Y2HGold yeast strain (Clontech, 630498). Yeast transformation was performed according to earlier publications^27^. First, yeasts were grown in 2xYPDA medium overnight at 30 °C, 200 rpm shaking. Afterwards, yeast cells were diluted to 0.4 optical density (OD, measured at 600 nm) and incubated in 2xYPDA for 5 h at 30 °C, 200 rpm shaking. Cells were harvested, washed with water and resuspended in 2 mL of 100 mM lithium acetate (LiAc). In total, 50 ml of this cell suspension was used for each transformation. Transformation mix included 1 µg of each vector (bait and prey), 60 µL of polyethylene glycol 50% (w/v in water), 9 µl of 1.0 M LiAc, 12.5 µL of boiled single-strand DNA from salmon sperm (AM9680, Ambion), and water up to 90 µl in total. The transformation mix was incubated at 30 °C for 30 min, and then at 42 °C for 30 min for the heat shock. The transformation mix supernatant was removed following centrifugation at 1000 *g* for 10 min, then cell pellets were resuspended in water, and plated first on -Leu -Trp plates to allow selective growth of transformants. After 2–3 days, growth transformants were inoculated into (-Leu, -Trp) medium, and were grown overnight at 30 °C, 200 rpm shaking. 10 µl of cell suspension diluted to both 0.5 and 1.0 OD in (-Leu, -Trp, -His, -Ade) medium were plated on (-Leu, -Trp) and (-Leu, -Trp, -His, -Ade) selective plates for 2–3 days to test for interactions. For media and plates preparation, we followed the manufacturer’s instructions.

### Immunofluorescence on meiocyte nuclear surface spreads

Preparation and immunostaining of nuclear surface spreads of spermatocytes and oocytes were carried out according to earlier described protocols with minor modifications^27,61^.

Testis cell suspensions were prepared in testis isolation medium (TIM): 100 mM NaCl, 45 mM KCl, 1.2 mM MgSO_4_, 0.6 mM KH_2_PO_4_, 0.1% glucose, 6 mM sodium lactate, 1 mM sodium pyruvate, pH 7.3. Decapsulated testes were incubated in 0.2 mg/ml collagenase (Sigma, C0130) solution for 50 min at 32 °C while shaking. Tubules were washed twice with TIM and incubated in 0.14 mg/ml trypsin solution (Worthington, LS003703) supplemented with 4 µg/ml DNAse I (Roche, 10104159001) for 15 min at 32 °C while shaking. Trypsin was inactivated by 1.25 mg/ml trypsin inhibitor (Gibco, 17075029), and cells were extensively resuspended. Cells were then washed twice with TIM (supplemented with 15 µl of 400 µg/ml DNAse I solution) and once with PBS pH 7.4. Cell suspensions were spun down at 1000 *g* for 5 min, and pellet was resuspended in 100 mM sucrose solution. Alternatively, testis cell suspensions were prepared in PBS pH 7.4, mixed with hypotonic extraction buffer (30 mM Tris-HCl pH 8.2, 50 mM sucrose pH 8.2, 17 mM sodium citrate pH 8.2, 5 mM EDTA pH 8.2, 0.5 mM DTT) in 1:1 ratio and incubated for 8 min at room temperature. After diluting the cell suspension five times in PBS pH 7.4, cell suspensions were centrifuged for 5 min at 1000 *g*, and cells were resuspended in 100 mM sucrose solution. Cell suspensions were added to 5–7 times higher volume droplets of filtered (0.2 μm) fixative (1% paraformaldehyde, 0.15% Triton X-100, 1 mM sodium borate pH 9.2) on diagnostic slides, and incubated for 90–180 min at room temperature in wet chambers. Nuclei were then dried for at least 1 h under fume hood.

To prepare nuclear surface-spread oocytes, two ovaries from each mouse were incubated in 20 µL hypotonic extraction buffer (HEB, 30 mM Tris-HCl, 17 mM Trisodium citrate dihydrate, 5 mM EDTA, 100 mM sucrose, 0.5 mM DTT, 0.5 mM PMSF, 1× protease inhibitor Cocktail) for 15 min. After incubation, HEB solution was removed and 16 µL of 100 mM sucrose in 5 mM sodium borate buffer pH 8.5 was added. Ovaries were punctured by two needles to release oocytes. In all, 9 µL of 65 mM sucrose in 5 mM sodium borate buffer pH 8.5 was added to the cell suspension, and incubated for 3 min. After mixing, 1.5 µL of the cell suspension was added in a well containing 20 µL of fixative (1% paraformaldehyde, 50 mM sodium borate buffer pH 9.2, 0.15% Triton X-100) on a glass slide. Cells were fixed for 45 min in humid chambers, then slides were air-dried. Upon completion of drying slides were washed in 0.4% Photo-Flo 200 (Kodak) and distilled water and dried at room temperature.

For immunostaining of nuclear surface spreads, slides were blocked for 1 h at room temperature with one of the following blocking solutions: (1) 1% Normal Goat Serum (NGS), 3% BSA, 0.02% Triton X-100 in TBS pH 7.6; (2) 2.5% BSA, in PBS pH 7.4 with 0.05% Tween 20 (PBS-T); (3) 5% NGS in PSB-T. After blocking, slides were incubated overnight or for 3 h at room temperature with following primary antibodies (see also Supplementary Table 2) diluted in blocking solution: guinea pig anti-PRR19 (this study, 1:1000), rabbit anti-PRR19 (this study, 1:500), rabbit anti-CNTD1 (this study, 1:500), mouse anti-SYCP3 (gift from R. Jessberger, 1:2)^62^, chicken anti-SYCP3 (1:600)^26^, chicken anti-SYCP1 (1:300)^27^, mouse anti-MLH1 (#3515, Cell Signaling, 1:50), rabbit anti-MLH1 (PC56, Calbiochem, 1:50), mouse anti-CDK2 (sc-6248, Santa Cruz, 1:200), rabbit anti-MLH3 (gift from P. Cohen, 1:1000)^6^, mouse anti-γH2AX (05-636, Millipore, 1:6000), rat anti-RPA2 (#2208, NEB, 1:100), mouse anti-RAD51 (MA5-14419, Thermo Fisher, 1:200), rabbit anti-DMC1 (sc-22768, Santa Cruz, 1:200), rabbit anti-MSH4 (ab58666, Abcam, 1:200), guinea pig anti-RNF212 (1:50)^14^, goat anti-RNF212 (this study, 1:100). Slides were then washed three times with PBS-T and incubated for 1 h at room temperature with goat or donkey secondary antibodies conjugated with AlexaFluor (AF) 488, 568 or 405 (A11034, A11036, A11073, A11075, A11031, A11029, A31553, A11039, A11041, A10037, A11055 Molecular Probes/Invitrogen; ab175675, Abcam; A11006, Life Technologies; see also Supplementary Table 3). Secondary antibodies were diluted in blocking solution 1:600 (for AF488 and AF568) or 1:400 (for AF405). Slides were then embedded in SlowFade™ Gold Antifade Mountant with or without DAPI (Invitrogen). Prior to immunostaining with mouse anti-HEI10 (ab118999, Abcam, 1:150), rabbit anti-proteasome 20 S alpha+beta (ab22673, Abcam, 1:200) or mouse anti-mono- and polyubiquitinylated conjugates (BML-PW8810-0100, Enzo Life Sciences, 1:500) antibodies, nuclear surface spreads were treated with 5 µg/ml pepsin solution in 10 mM HCl for 3 min at room temperature. This was followed by three washes with PBS-T, one wash with DNAse I buffer and treatment with DNAse I (Thermo Scientific, EN0525) diluted 1:100 in DNAse I buffer for 15 min at 37 °C. After three washes with PBS-T, slides were blocked and stained as described above. Histone H1t immunostaining was performed sequentially. Slides were incubated for 3 h at room temperature with guinea pig anti-histone H1t (gift from M. A. Handel, 1:500)^28^, guinea pig or rabbit anti-histone H1t (1:20000)^27^ primary antibodies diluted in blocking solution, followed by washes and 1 h incubation with secondary antibodies: donkey anti-guinea pig conjugated with DyLight 405 (706-475-148, Jackson ImmunoResearch, 1:200) or goat anti-rabbit conjugated with AF405 (A31556, Molecular Probes/Invitrogen, 1:400). All incubations were performed in closed wet chambers.

Ubiquitin and proteasome signals were quantified along the chromosome axes in mid pachytene spermatocyte spreads using Fiji software. Signal intensity was measured in a region next to each analysed cell to allow background correction. For proteasome staining, the total signal along all chromosomes was compared between wild-type and PRR19-deficient cells. Ubiquitin hyperaccumulates in pachytene spermatocytes in the sex body, and this ubiquitin accumulation is not thought to be relevant for crossover differentiation. Hence, only autosomes that did not overlap with the sex body were taken into account for ubiquitin signal quantification. The average signal intensity per chromosome axis area was calculated to allow comparison between cells where autosomal axes overlapped or did not overlap with sex bodies. Absolute intensities of ubiquitin and proteasome staining varied between experiments. Hence, background-corrected signal intensities were normalised to the median of wild-type signal intensities within each experiment, to allow comparison and pooling of experiments. Focus numbers of DSB repair and crossover markers were counted manually on matched exposure images of wild-type and mutant nuclear spreads of meiocytes.

### Immunofluorescence on gonad sections

Testis cryosections were prepared according to published protocols^27^. Testes were fixed with 3.6% formaldehyde, 0.1% Triton X-100 in 100 mM sodium phosphate buffer pH 7.4, at room temperature for 40 min. Following fixation and three washes in PBS pH 7.4, testes were placed into 30% sucrose solution overnight at 4 °C and then frozen on dry ice in Tissue-Tek^®^ O.C.T. Compound (Sakura Finetek Europe). In total, 8-µm-thick sections of testes were cut and dried onto slides. The sections were washed in water and PBS pH 7.4, and immediately used for immunofluorescence staining following blocking in 2.5% BSA in PBS pH 7.4, 0.05% Tween 20, 0.05% Triton X-100. Sections were incubated for 3 h at room temperature with rabbit anti-cleaved PARP (9544 S, Cell Signalling, 1:250) and guinea pig H1t^27^ primary antibodies diluted in blocking solution. Following washes, sections were incubated for 1 h at room temperature with secondary antibodies in blocking solution. H1t immunostaining was performed sequentially. To assess oocyte numbers, sections of ovaries of 6–7-week-old females were prepared by published methods^27^ with minor modifications. Ovaries were embedded in paraffin for serial sectioning at 5-µm thickness. Every 6th section from each ovary was collected for further immunostaining and follicle quantification. Deparaffinisation and rehydration of the sections was performed as follows: 2 × 5 min in xylene, 2 × 5 min in 100% ethanol, 5 min each in 95%, 85%, 70%, 50% ethanol, 2 × 5 min in water. Sections were subjected to heat-mediated antigen retrieval in 10 mM Sodium citrate, 0.05% Tween 20, pH 6.0 for 20 min on boiling water bath. Sections were permeabilised in PBS with 0.2% Triton X-100 for 45 min at room temperature and processed for immunofluorescence staining immediately. Ovary sections were incubated with mouse anti-MVH (ab13840, Abcam, 1:500) and rabbit anti-p63 (CM163A, Biocare Medical, 1:300) primary antibodies overnight at 4 °C. Following washes with PBS-T, secondary antibodies were applied for 1 h at room temperature. Thereafter, sections were incubated in 0.1% Sudan Black B (Sigma, 199664) solution in 70% ethanol for 10 min in the dark, followed by extensive washes with PBS-T. All oocytes were counted on every 6th section, and numbers from both ovaries of each female were summed up. Following immunostaining, testis and ovary sections were stained with 5 µg/ml DAPI and embedded in SlowFade™ Gold Antifade Mountant with DAPI (Invitrogen).

### Staging of mouse seminiferous tubule cross sections

To stage the epithelial cycle of mouse seminiferous tubules, we used criteria that were described earlier^27,63^. We identified stages of seminiferous tubules as follows. Stages I–IV: a basal layer featuring large spermatogonia A and intermediate spermatogonia (dark DAPI staining of most chromatin with occasional and small DAPI-bright heterochromatic regions) and a second cell layer consisting of early pachytene cells, which are negative for histone H1t. Stages V–VI: a basal layer containing spermatogonia B (oval shaped nuclei with more and larger round-shaped DAPI-bright heterochromatic regions than in intermediate spermatogonia) and a second cell layer consisting of mid pachytene cells, which express low or intermediate levels of histone H1t. Stages VII–VIII: a basal cell layer consisting of preleptotene spermatocytes, which have smaller and more round nuclei than spermatogonia B, the chromatin is also more DAPI-bright and have bigger and more round heterochromatin aggregates than spermatogonia B. Preleptotene cells form almost continuous basal layer as their number is the double of spermatogonia B numbers. The second cell layer consists of late pachytene cells that have strong H1t signal. Stage I–VIII tubules contain round spermatids luminal to the pachytene spermatocytes and advanced elongated spermatids or sperm in the most luminal cell layer. Stage IX: a basal cell layer contains leptotene cells, which have round nuclei with intense overall DAPI staining and fewer and larger heterochromatin aggregates than preleptotene cells. The second cell layer consists of late pachytene/diplotene cells which are strongly H1t-positive. Elongating spermatids are found in the lumen. Stages X–XII: a basal cell layer contains zygotene cells which have bright overall DAPI staining that is less homogeneous than in leptotene. Zygotene cells also have flatter heterochromatic aggregates at the nuclear periphery than leptotene cells. In stage X–XI, the second cell layer consists of histone H1t-stained diplotene cells followed by elongated spermatids toward the lumen. In stage XII tubules, diplotene cells enter the first and the second meiotic divisions, therefore metaphase cells and secondary spermatocytes are between the basal zygotene cell layer and the luminal elongated spermatid layer.

### Diakinesis/metaphase I chromosome spreading

Chromosome spreads of diakinesis/metaphase I stage spermatocytes were prepared as described in refs. ^5,27^. Testes were decapsulated, and tubules were disrupted in hypotonic buffer (1% trisodium citrate in water). Large clumps were removed and the cell suspension was incubated in hypotonic buffer for 20 min at room temperature. Cell suspension was centrifuged at 200 *g* for 10 min, and supernatant was removed. Afterwards, cells were fixed in a methanol/acetic acid/chloroform (3:1:0.05 ratio) fixative, centrifuged and resuspended in ice-cold methanol/acetic acid solution (3:1 ratio). Fixed cells were dropped onto slides, dried quickly (in humid conditions), and stained with Hoechst 33342.

Oocyte metaphase I spreads were prepared as described in ref. ^57^. Oocytes were collected from antral follicles of 9–16-week-old mice and placed in the M2 medium (Sigma, M7167) supplemented with 2.5 µM milrinone (Sigma, M4659), which maintained oocyte arrest in the germinal vesicle (GV) stage. Oocytes were induced to undergo meiotic maturation by rinsing and culture in M2 medium without milrinone. Only oocytes, in which GV breakdown (GVBD) was observed within 90 min after release, were used for further experiments. After 6 h incubation at 37 °C, zona pellucida was removed by treating oocytes with Tyrode’s solution (Sigma, T1788) and the oocytes were fixed in 1% paraformaldehyde, 0.15% Triton X-100, 1 mM DTT, pH 9.2 on diagnostic slides. The slides were dried, blocked with 2.5% BSA in PBS-T and immunostained with human anti-centromere protein (15-235, Antibodies Inc, 1:1000) followed by goat anti-human secondary antibodies conjugated with AF568 (A21090, Molecular Probes/Invitrogen, 1:300). Following immunostaining, slides were embedded in SlowFade™ Gold Antifade Mountant with DAPI (Invitrogen).

### Protein alignment

Vertebrate orthologs can be found with NCBI-BLASTp searches (blast+ version 2.6.0)^64^ in the NCBI nr protein database or the UniProt reference proteome database applying significant e-value thresholds (<= 1e-3), using human PRR19 as reference sequence (UniProtKB accession: A6NJB7). The vertebrate PRR19 protein family includes sequences from *Mus musculus* (UniProtKB: B2RW88), *Gallus gallus* (NCBIprotein: XP_004937082.2), *Chelonia mydas* (UniProtKB: M7CAQ8), *Xenopus laevis* (UniProtKB: A0A1L8FN18), and *Danio rerio* (UniProtKB: F1QJ91). Sequences were aligned with MAFFT (-linsi v7.427)^65^ and visualised in Jalview^66^. Besides proline-rich segments scattered over the entire protein sequence (detected by fLPS)^67^, four conserved regions were identified (CR1-CR4, Fig. S2). CR1 is arginine-rich, and short secondary structure elements can be predicted for all regions^68^, but no functional motifs were detected. To find orthologs in non-vertebrate species, we searched with a hidden Markov model covering CR2 and CR3 in eukaryotic UniProt reference proteomes (HMMER, version 3.2.1)^69^. Significant hits were detected in the scallop *Mizuhopecten yessoensis* (E-value 3.1e-05, UniProtKB: A0A210QF54), the lancelet *Branchiostoma floridae* (E-value 0.00055, UniProtKB: C3Y2J1) and the brachiopod *Lingula unguis* (*E*-value 0.0067, UniProtKB: A0A1S3HP68). NCBI-BLASTp searches with the invertebrate sequences identified family members in other molluscs, in echinoderms, including *Strongylocentrotus purpuratus* (UniProtKB: W4Z018), in the annelid worm *Capitella teleta* (NCBIprotein: ELT87451.1), and in cnidarian, such as the stony coral *Stylophora pistillata* (NCBIprotein: XP_022782443.1), applying significant E-values. They all share similar domain architecture, with conserved regions CR1-4 and proline-rich segments.

Multiple sequence alignments of mouse CNTD1 protein sequence with either *C. elegans* COSA-1 or mouse cyclin E1 (CCNE1), A2 (CCNA2) and B1 (CCNB1) protein sequences were performed using Clustal Omega tool at https://www.ebi.ac.uk/Tools/msa/clustalo/^70^.

Modelling CNTD1 domain structure (Supplementary Fig. 13b) was based on CNTD1 alignment with cyclin A2 by SWISS-MODEL (https://swissmodel.expasy.org/)^71–73^.

### Statistics and reproducibility

Graphs were prepared using GraphPad Prism 7 and statistical analysis was done using R version 3.3.3. The types of the statistical tests and p-values are indicated in the corresponding figure legends. No mathematical correction was made for multiple comparisons.

In order to compare the frequencies of cell phenotype categories in mutant and wild-type cells, percentages of cells obtained from multiple experimental repetitions were analysed within a generalised linear mixed-effects model framework. The effects of cell categories and genotypes were assumed to be fixed, and the experiment effect was assumed to be random. The significance of the interaction between the cell categories and the genotypes was assessed using a likelihood-ratio test. The likelihood-ratio test is used to compare the goodness of fit of two statistical models based on the analysis of deviance. In this context, neither the test hypotheses nor the test itself are referred to as one- or two-sided. The likelihood-ratio statistic is always positive and has the chi-squared distribution with appropriate number of degrees of freedom. The distribution is one-tailed, and a p-value is always computed as the area of the tail to the right of the observed test statistic. The statistical analysis was implemented in the R package lme4^74,75^. In cases, where the quantification of cell categories was performed only once (Supplementary Fig. 5b, c) or proportions of a category were 0% in experimental repeats, the likelihood-ratio test could not be applied, hence, the distributions were compared using Fisher’s exact test.

All phenotypes were observed in at least two animals of each genotype and, unless stated otherwise, quantifications represent analysis of at least two independent animals. All comparisons were made between datasets obtained from animals that were either littermates or matched by age.

Reproducibility of representative experiments: immunoprecipitations and immunoblots of testis extracts from mutant adult animals (Fig. 2c, d; Supplementary Fig. 3b) as well as immunofluorescence in the nuclear surface-spread meiocytes from mutant adult animals (Figs. 2e, f, 7a) were performed in at least three independent experiments; RT-PCR images (Fig. 2b; Supplementary Fig. 3c) represent data obtained from a single experiment. Y2H images (Fig. 7i; Supplementary Figs. 12a, 13a, 14a, b) represent data observed in at least two independent repetitions of experiments.

### Biological materials availability

Transgenic mouse strains, plasmids and antibodies produced in this study are available from the authors upon request.

### Reporting summary

Further information on research design is available in the Nature Research Reporting Summary linked to this article.

## Supplementary information

Supplementary Information

Reporting Summary

## Data Availability

Image sets underlying quantitative data are available from the corresponding author upon request. All other data supporting the findings of this study are available within the paper, its supplementary files and referenced published datasets. The source data underlying Figs. 1a, b, e, g, j, 2b-d, 3a-c, 4b, d, f, h, 5b, d, f, h, 6a, c-d, h, j and 7c, d, f–h and Supplementary Figs. 1a–c, g, 3b, c, 4g, h, 5b–e, 6a, b, d, 7b, 8b, c, 9b, d and 10b are provided as a Source Data file. Databases used in the study: ENCODE project (data source, BioProject: PRJNA66167)^25^, Gene Expression Omnibus (GEO) database [https://www.ncbi.nlm.nih.gov/geo/query/acc.cgi?acc=GSE119411]^27^, NCBI nr protein database (available at ftp://ftp.ncbi.nlm.nih.gov/blast/db/FASTA/nr.gz), UniProt reference proteome database (available at ftp.uniprot.org). Source data are provided with this paper.

## Source data

Source Data

## Code availability

The R code used for statistical analysis is provided as a Supplementary Methods file.
