# Supplementary material for: Proline-rich protein PRR19 functions with cyclin-like CNTD1 to promote meiotic crossing over in mouse
Source: Nat Commun. 2020 Jun 18;11:3101. doi: 10.1038/s41467-020-16885-3 (PMC7303132; doi:10.1038/s41467-020-16885-3)
Supplement: Supplementary file 1 — Supplementary Information [file 41467_2020_16885_MOESM1_ESM.pdf]

Supplementary Information

Proline-rich protein PRR19 functions with cyclin-like CNTD1 to promote meiotic crossing over in mouse

Bondarieva A. et al.

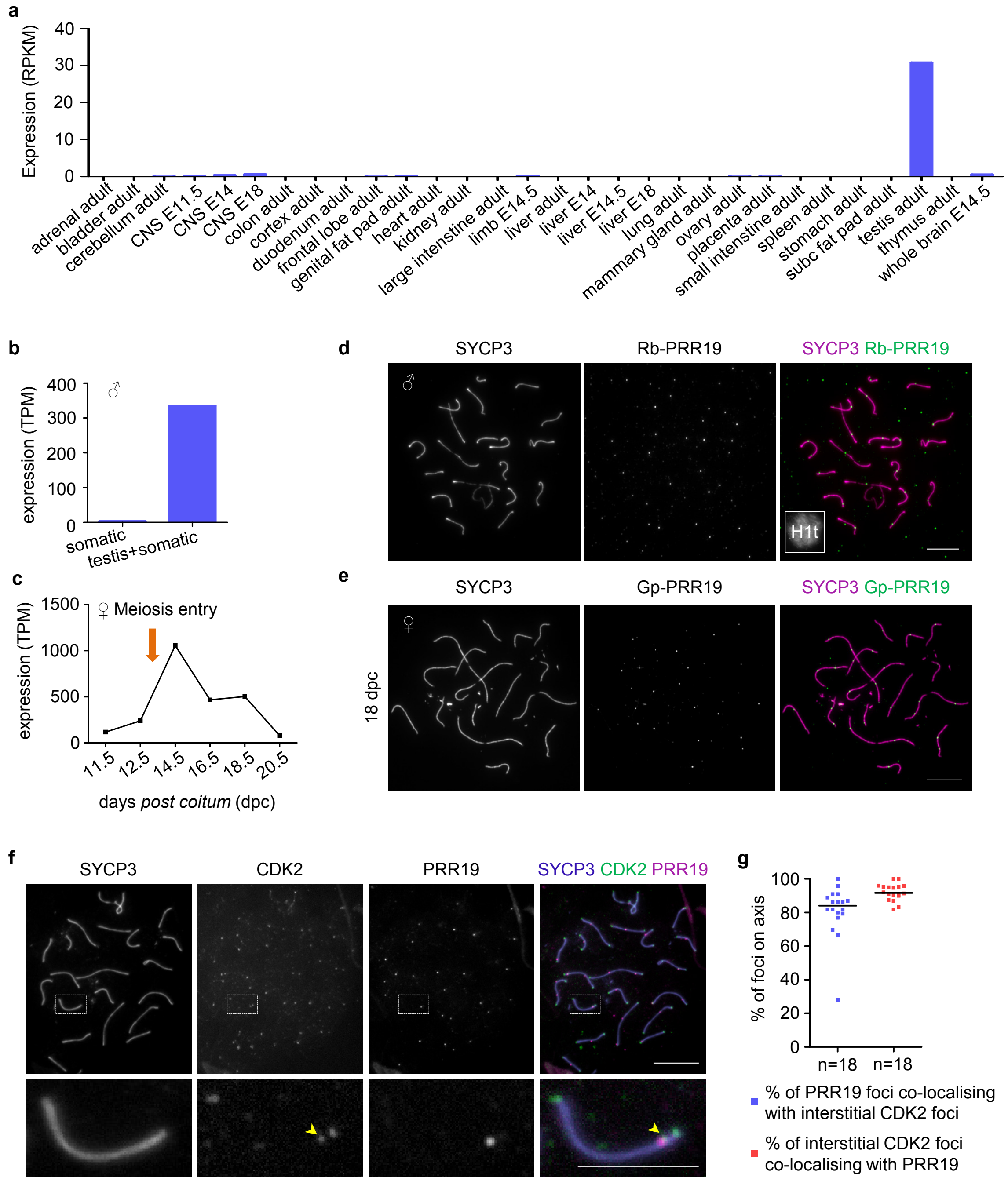

Supplementary Figure 1. PRR19 localises to crossover-specific recombination complexes.
(**a**) *Prr19* transcript levels are shown as reads per kilobase million (RPKM) in RNAs of indicated tissues from the ENCODE project (data source, BioProject: PRJNA66167; https://www.ncbi.nlm.nih.gov/gene/623131/?report=expression) [25]. (**b-c**) *Prr19* transcript levels are shown as transcripts per kilobase million (TPM) in (**b**) mixtures of equal quantities of total RNAs of 17 somatic tissues (somatic) or 17 somatic tissues plus adult testes (testis+somatic), or (**c**) total RNAs of foetal ovaries at the indicated developmental stages (data source, GSE119411, dataseries[27] from the Gene Expression Omnibus database**,** https://www.ncbi.nlm.nih.gov/geo/query/acc.cgi?acc=GSE119411) (**a-c**) Presented data come from a single experiment. (**d-f**) Indicated proteins were detected by immunofluorescence in nuclear spread (**d, f**) mid pachytene spermatocytes, or (**e**) pachytene oocytes from foetuses at 18 days *post coitum* (dpc). Enlarged insets (bottom panel, **f**) show co-localisation of PRR19 and crossover-specific CDK2 interstitial foci (marked by arrowhead). Bars, 10 µm; on enlarged insets (**f**), 5 µm. (**g**) Quantification of co-localisation between PRR19 and interstitial CDK2 foci on the chromosome axis in mid pachytene wild-type spermatocytes, medians (bars) are 84.1% (blue dataset) and 91.7% (red dataset). n=number of analysed cells from a single mouse. Source Data are provided as a Source Data file.

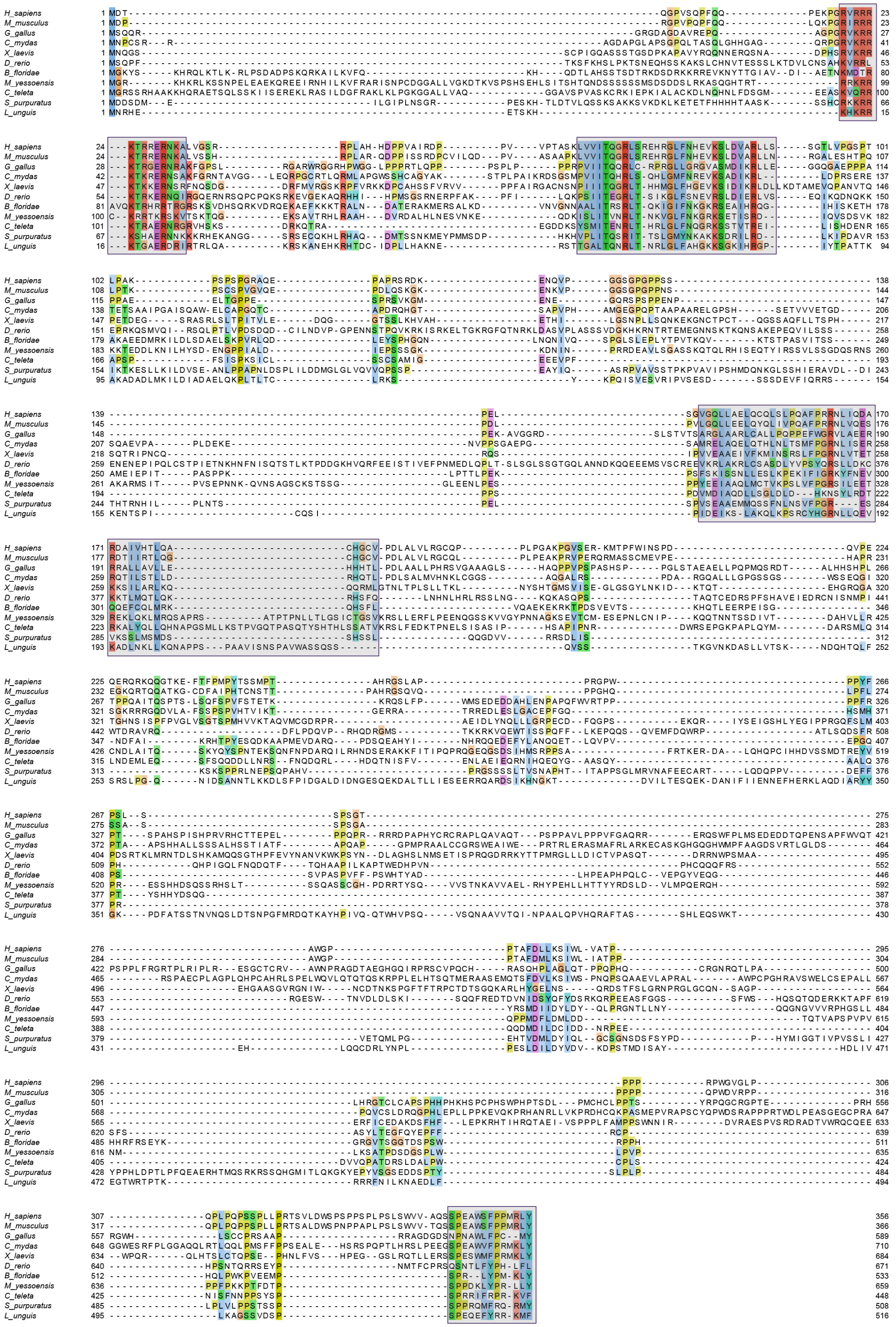

Supplementary Figure 2. Multiple alignment of PRR19 protein sequences from various animal taxa.
Sequences are aligned from human (*H_sapiens*), house mouse (*M_musculus*), chicken (*G_gallus*), green sea turtle (*C_mydas*), african clawed frog (*X_leavis*), zebrafish (*D_rerio*), lancelet (*B_floridae*), scallop (*M_yessoensis*), polychaete annelid worm (*C_teleta*), purple sea urchin (*S_purpuratus*), brachiopod (*L_unguis*). Default ClustalX colour scheme was used. Boxes indicate four conserved motives within PRR19 protein sequence. The conserved regions span R19-A32, L61-S91, V144-V191, S344-Y356 sequences in the human PRR19.

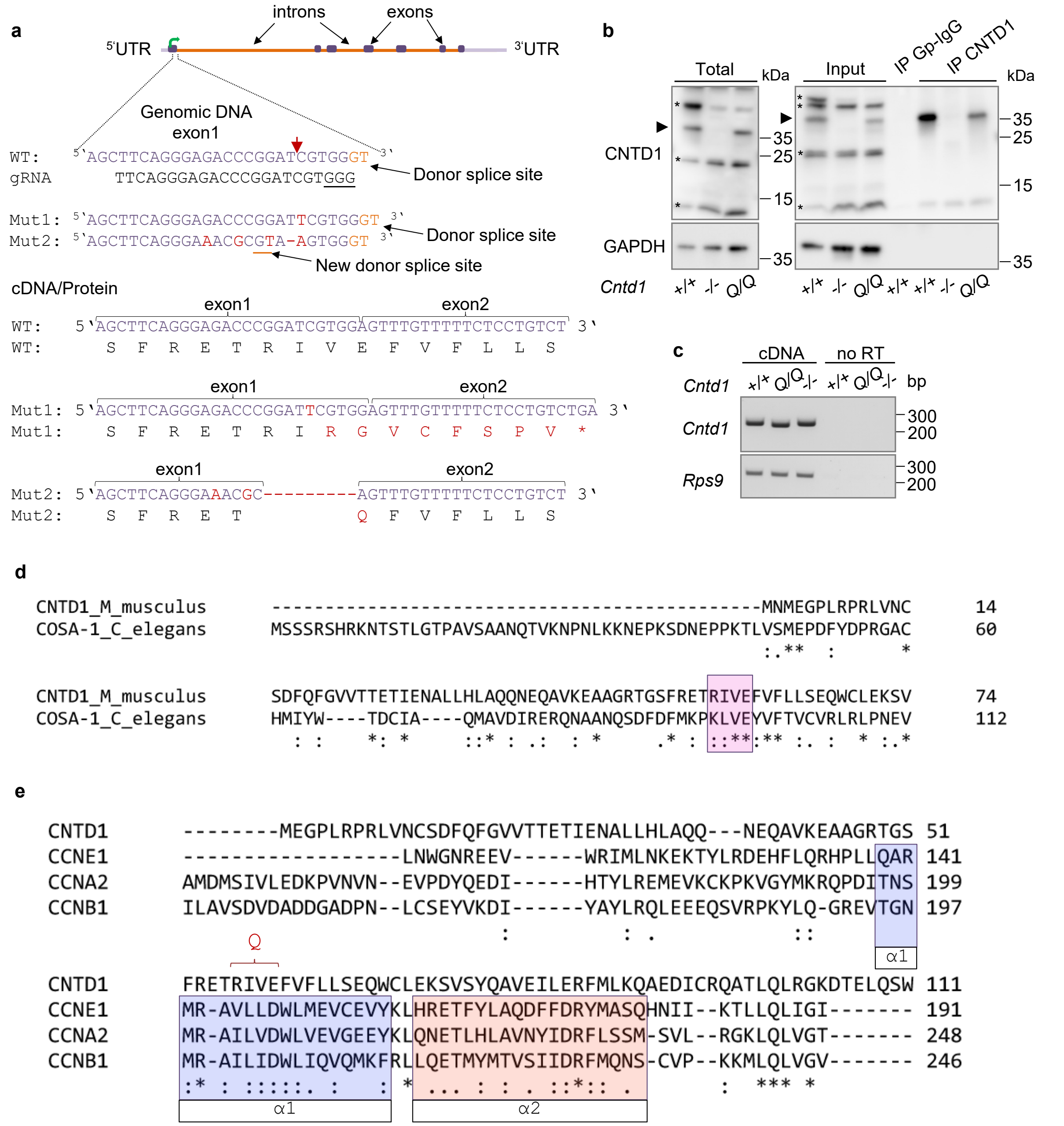

Supplementary Figure 3. Generation of *Cntd1*-mutant lines.

(**a**) Schematics of the *Cntd1* locus show exons, introns, translated regions (dark lilac boxes), untranslated regions (5'- and 3'-UTR, light lilac boxes) and the start of open reading frame (green arrow) in exon 1. Expanded view of exon 1 shows the guide RNA sequence (gRNA, with PAM underlined in black) used for CRISPR/Cas9 editing by Cas9 nuclease, the genomic DNA sequence of the edited exon 1 region (lilac) with the associated donor splice site (orange), the corresponding cDNA sequence of exon1-exon2 junction and protein sequences in wild-type (WT) and *Cntd1* mutant lines (Mut1 and Mut2). DNA break site is marked by red arrow; red text colour marks mutated DNA and protein sequences; new donor splice site in Mut2 is underlined with orange. (**b**) Immunoblot analysis of total protein extracts (Total), or anti-CNTD1 immunoprecipitations from testes of adult wild-type (+/+), Cntd$1^{-/-}$ and *Cntd1*$^{Q/Q}$ mice. Blot pictures show inputs for immunoprecipitations, control immunoprecipitates with guinea pig IgG isotype antibodies (IP Gp-IgG) and immunoprecipitates with guinea pig antibodies against the C-terminal C126-T249 peptide of CNTD1 (IP CNTD1). Upper and lower panels show detection of proteins by rabbit anti-CNTD1 antibodies (raised against the same peptide as guinea pig anti-CNTD1) and anti-GAPDH antibodies (loading control), respectively. Arrowhead marks presumed band of CNTD1, asterisks mark unspecific protein bands in upper panels. Molecular weight marker positions are indicated. (**c**) Agarose gel pictures show RT-PCRs using primers that match (upper panel) either *Cntd1* exon 1 and exon 2 sites outside of the targeted region, or (lower panel), as a control, cDNAs of the 40S ribosomal protein S9 (*Rps9*). Total testis RNAs of adult wild-type (+/+), *Cntd1*$^{Q/Q}$ and Cntd$1^{-/-}$ mice served as templates. Samples without reverse transcriptase (no RT) show RT-PCR specificity. DNA length marker positions are indicated. (**d**) Alignment of N-terminal parts of CNTD1 protein sequence from *M. musculus* and COSA-1 protein sequence from *C. elegans*. Pink box indicates conserved amino acids RIVE that are mutated to Q in *Cntd1*$^{Q/Q}$ mutant. (**e**) Sequence alignment of the first 110 amino acids of mouse CNTD1 with mouse cyclins E1 (CCNE1), A2 (CCNA2) and B1 (CCNB1). Blue and orange boxes indicate the conserved regions corresponding to α1 and α2 helices of cyclin box. Red bracket indicates RIVE sequence of CNTD1 within the predicted α1 helix; this sequence is mutated in *Cntd1*$^{Q/Q}$ mutant. An "*" (asterisk) indicates positions which have a fully conserved residue. A ":" (colon) indicates conservation between groups of strongly similar properties, scoring > 0.5 in the Gonnet PAM 250 matrix. A "." (period) indicates conservation between groups of weakly similar properties, scoring =< 0.5 in the Gonnet PAM 250 matrix. Source data are provided as a Source Data file.

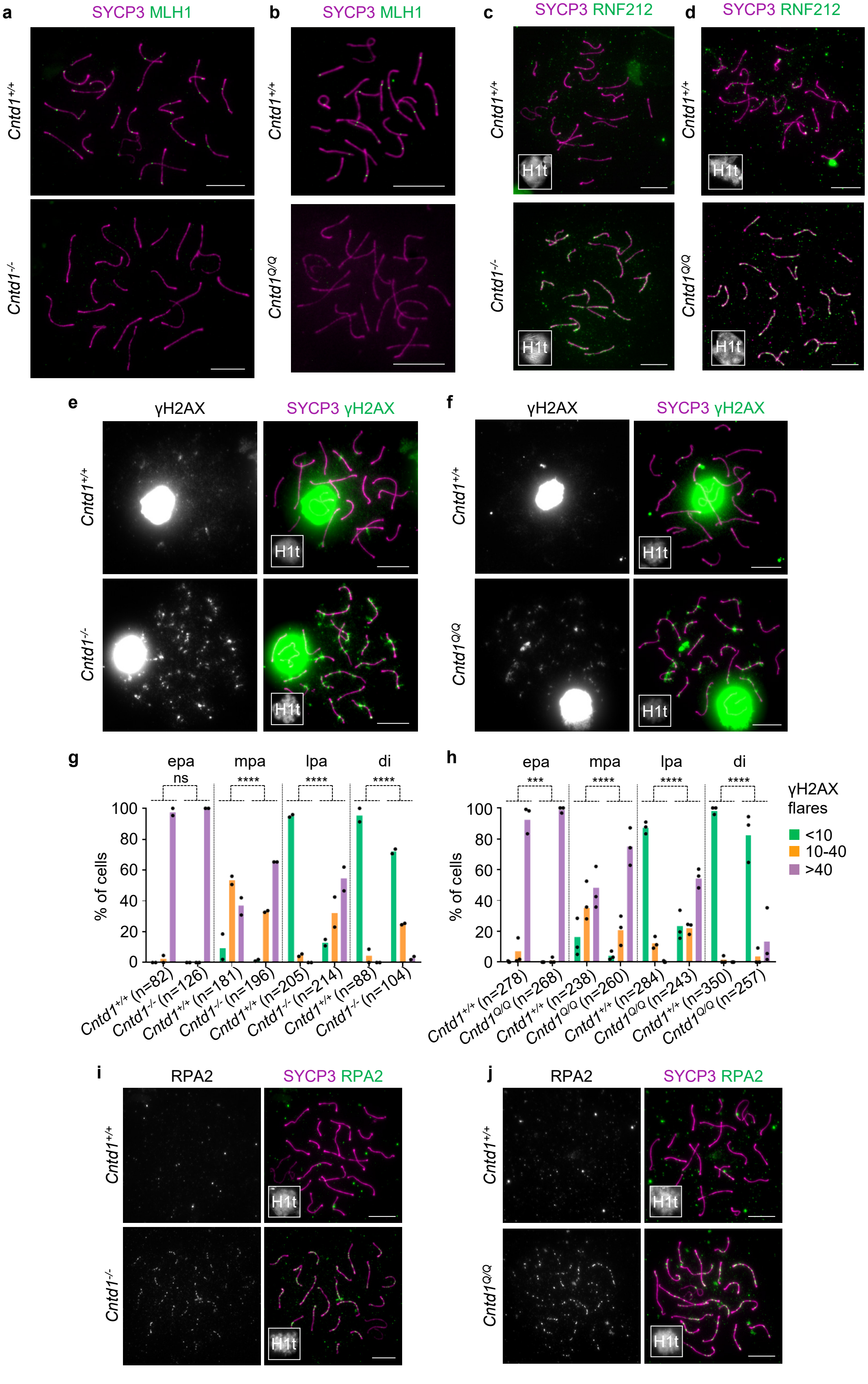

Supplementary Figure 4. *Cntd1$^{-/-}$* and *Cntd$^{Q/Q}$* spermatocytes show similar defects in DSB repair, crossover formation and RNF212 kinetics.

(**a-f, i-j**) Indicated proteins were detected by immunofluorescence in nuclear surface-spread spermatocytes of wild-type, Cntd*1$^{-/-}$* and *Cntd1$^{Q/Q}$* mice; mid or late pachytene cells are shown. Miniaturised H1t signal of the corresponding cell is shown in the bottom left corner of images (**c-f, i-j**). Saturated γH2AX signal (**e-f**) corresponds to the silenced chromatin of sex chromosomes, which is a chromatin compartment where histone γH2AX hyper-accumulates in pachytene and diplotene stages. Bars, 10 μm. (**g-h**) Quantification of γH2AX flare numbers in early (epa), mid (mpa), late pachytene (lpa) and diplotene (di) spermatocytes. Three categories were distinguished, spermatocytes with less than 10 flares, between 10 and 40 flares and more than 40 flares of γH2AX on autosomes. Graph shows datapoints and weighted averages of percentages of spermatocytes in the three categories, n=numbers of analysed cells from two (**g**) or three (**h**) experiments. An analysis of deviance using the likelihood-ratio test based on the chi-squared distribution was used to calculate if the proportions of cells with distinct numbers of γH2AX flares significantly differ between wild type and Cntd*1$^{-/-}$* (epa, P=0.1531, mpa, P=1.53E-08, lpa and di, P=2.20E-16) or *Cntd1$^{Q/Q}$* (epa, P=0.0004531, mpa, P=4.36E-10, lpa, P=2.20E-16, di, P=3.02E-15). ns, *** and **** indicate no significance (P>0.05), 0.001>P>0.0001 and P<0.0001, respectively. Source data are provided as a Source Data file.

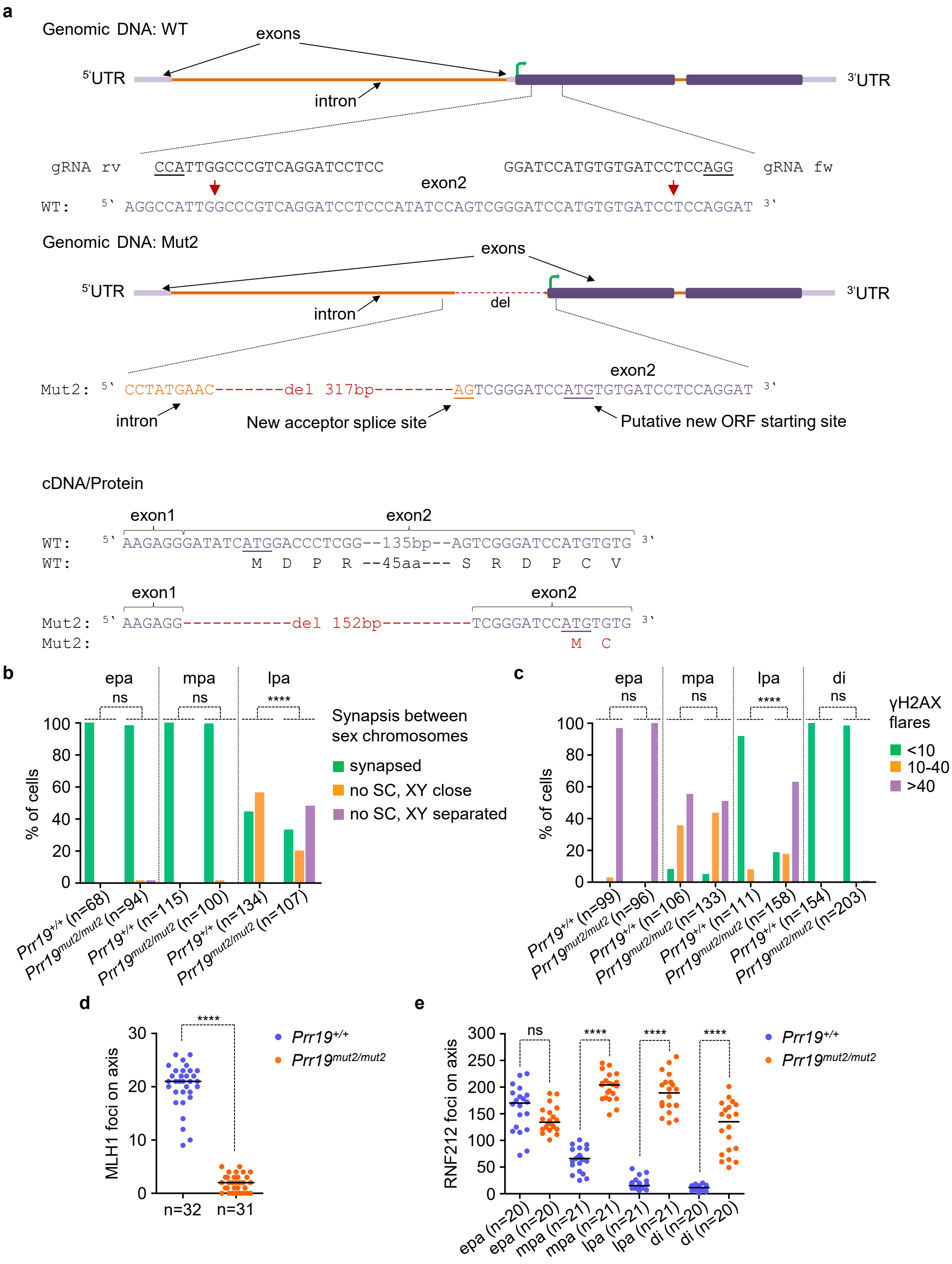

Supplementary Figure 5. *Prr19* Mut2 spermatocytes are defective in DSB repair and crossover differentiation.
(**a**) Schematics of the *Prr19* locus in wild-type or Mut2 mice. Schematics show introns (orange), exonic translated regions (dark lilac boxes), exonic untranslated regions (5'- and 3'-UTR, light lilac boxes), deleted region (red dashes) and the start of open reading frame (ORF, green arrow) in exon 2. Expanded view of exon 2 from wild-type locus shows guide RNA sequences (gRNA rv and gRNA fw with PAMs underlined) and the sites (red arrows) that were cut by Cas9 nickase. Expanded view of the modified genomic region of *Prr19* Mut2 allele shows the intron and exon sequences flanking an 317 bp deletion; a new acceptor splice site that emerge in exon 2 due to the 317 bp deletion is underlined (orange), the first ATG codon that is expected to serve as a new out of frame ORF start site is also underlined (lilac). *Prr19* cDNAs of wild-type and Mut2 mice are shown with the corresponding protein sequences underneath. Start codons of ORFs are underlined in cDNAs. (**b**) Quantification of synapsis between sex chromosomes in wild-type and *Prr19*$^{mut2/mut2}$ spermatocytes. Pachytene spermatocytes were sub-staged to early (epa), mid (mpa) and late pachytene (lpa) based on histone H1t staining. Synapsis marker SYCP1 was detected and fractions of cells with distinct degrees of synapsis and pairing between sex chromosomes were quantified. Graph shows fractions of spermatocytes with synapsed sex chromosomes, unsynapsed but closely paired sex chromosomes (no SC, XY close) or unsynapsed sex chromosomes that separated (no SC, XY separated). (**c**) Quantification of γH2AX flare numbers in early (epa), mid (mpa), late pachytene (lpa) and diplotene (di) spermatocytes. Three categories were distinguished, spermatocytes with less than 10 flares, between 10 and 40 flares and more than 40 flares of γH2AX on autosomes. (**b-c**) Counted cell numbers (n) and percentages of cells from a single experiment are shown. Fisher's exact test, (**b**) epa, P=1, mpa, P=0.4651, lpa, P=2.2E-16, (**c**) epa, P=0.2463, mpa, P=0.382, lpa, P=2.2E-16, di, P=0.508; ns and **** indicate no significance and P<0.0001, respectively. (**d**) Quantification of axis-associated MLH1 foci in mid/late pachytene spermatocytes. (**e**) Quantification of RNF212 axis-associated focus numbers in early (epa), mid (mpa), late pachytene (lpa) and diplotene (di) spermatocytes. (**d-e**) n=numbers of analysed cells from a single experiment and medians (bars) are indicated. Median focus numbers (bars) were compared using Mann-Whitney U test and are as follows in wild-type and *Prr19*$^{mut2/mut2}$ cells: MLH1 foci, 21 and 2 (P=7.91E-12), RNF212 foci, in epa, 170 and 134 (P=0.06783), mpa, 66 and 204 (P=2.89E-08), lpa, 15 and 189 (P=4.26E-08), di, 11.5 and 135 (P=6.22E-08), respectively. ns and **** indicate P>0.05 and P<0.0001, respectively. Source data are provided as a Source Data file.

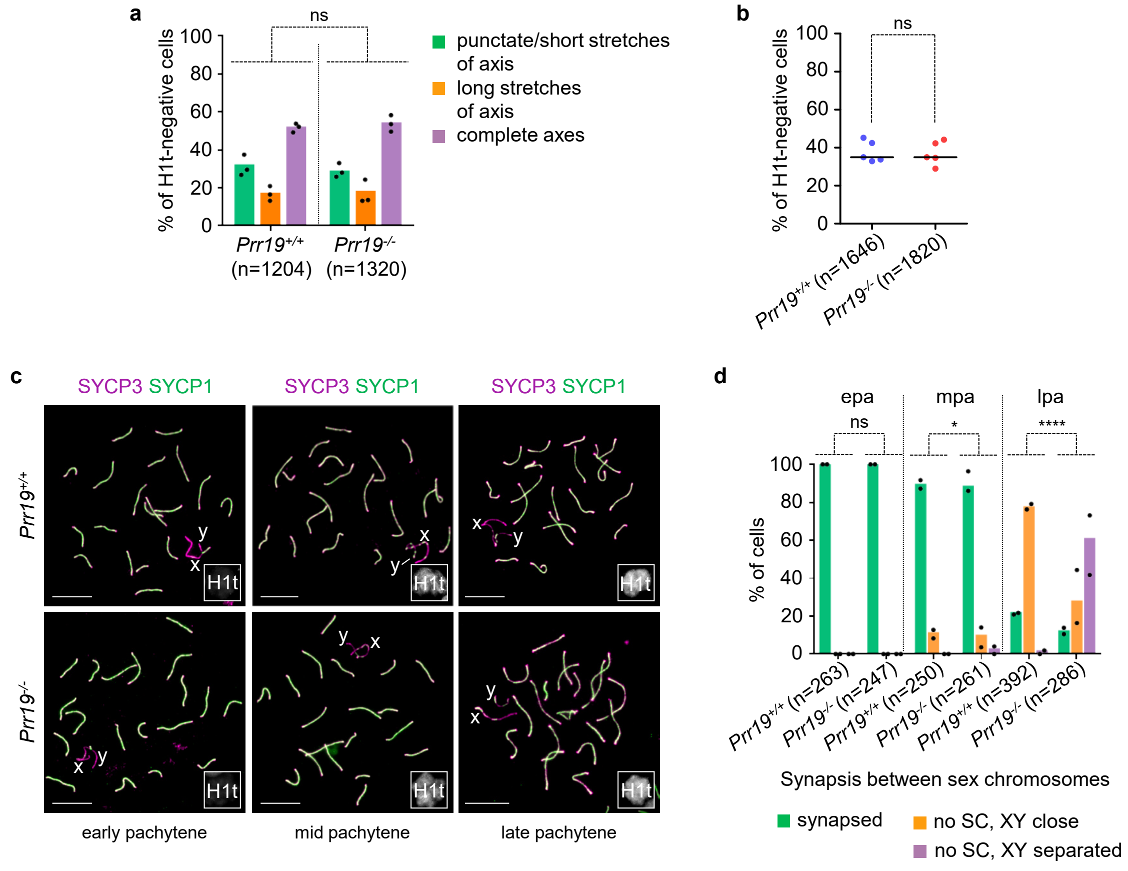

Supplementary Figure 6. Synapsis of autosomes is efficient in PRR19-deficient spermatocytes.
(**a**) Quantification of testicular cell populations with distinct chromosome axis morphologies. SYCP3 (axis marker) and histone H1t were detected in testicular cells from adult mice. Graph shows the proportion of pre-mid pachytene (H1t-negative) spermatocytes that have focal SYCP3 staining or short stretches of axis (punctate/short stretches of axis, corresponds to preleptotene and leptotene, respectively), long stretches of axis (corresponds to early zygotene) or fully formed axes on all chromosomes (complete axes, corresponds to late zygotene and early pachytene). Counted cell numbers (n), datapoints and weighted averages of percentages from three experiments are shown. An analysis of deviance using the likelihood-ratio test based on the chi-squared distribution indicated no significant difference (ns, P=0.2236). (**b**) Graph shows fraction of spermatocytes with fully synapsed autosomes in histone H1t-negative spermatocytes of adult wild-type and PRR19-deficient mice. Cell numbers (n) and medians (bars, 34.89% in both genotypes) from five experiments are shown. An analysis of deviance using the likelihood-ratio test based on the chi-squared distribution calculated no significant difference between wild type and *Prr19*$^{-/-}$ (ns, P=0.304). (**c**) Immunofluorescence of synapsis (SYCP1), axis (SYCP3) and prophase staging (histone H1t) markers in nuclear spread spermatocytes of wild-type and *Prr19*$^{-/-}$ mice. Prophase stages and the X and Y chromosomes are indicated, miniaturised images of histone H1t are shown in insets. Bars, 10 μm. (**d**) Quantification of synapsis between sex chromosomes in wild-type and *Prr19*$^{-/-}$ spermatocytes. Pachytene spermatocytes were sub-staged to early (epa), mid (mpa) and late pachytene (lpa) based on histone H1t staining. Synapsis marker SYCP1 was detected and fractions of cells with distinct degrees of synapsis and pairing between sex chromosomes were quantified. Graph shows fractions of spermatocytes with synapsed sex chromosomes, unsynapsed but closely paired sex chromosomes (no SC, XY close) or unsynapsed sex chromosomes that separated (no SC, XY separated). Counted cell numbers (n), datapoints and weighted averages of percentages from two experiments are shown. An analysis of deviance using the likelihood-ratio test based on the chi-squared distribution calculates no significant difference (ns, P=1) in early pachytene, and significant differences in mid (*,P=0.01603) and late (****, P=2.2E-16) pachytene. Source data are provided as a Source Data file.

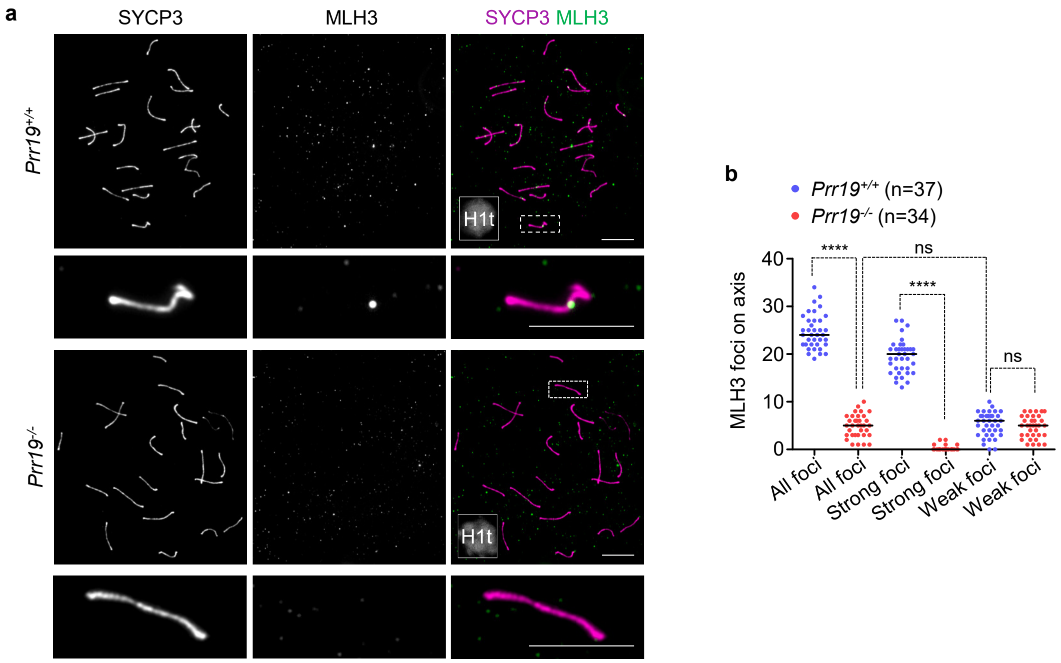

Supplementary Figure 7. PRR19 is required for the formation of class I crossover-specific recombination complexes.
(**a**) Class I crossover marker MLH3 was detected by immunofluorescence in spreads of mid pachytene spermatocytes from wild-type and *Prr19*$^{-/-}$ mice. Enlarged insets (bottom panels) show the presence or absence of a strong crossover-specific MLH3 focus on chromosomes in wild-type or *Prr19*$^{-/-}$ spermatocyte, respectively. Bars, 10 µm. (**b**) Quantification of axis-associated foci of MLH3. Due to the high level of background staining, the strong focal staining of MLH3 was quantified separately from weak foci which were similar to non-axis-associated background foci in intensity. Counts of strong and weak foci are shown separately and pooled (all foci). n=numbers of analysed cells from two experiments. Medians (bars) were compared by Mann-Whitney U test and are as follows in wild type and *Prr19*$^{-/-}$: all foci, 24 and 5 (****, P=4.12E-13), strong foci, 20 and 0 (****, P=6.0E-14), and weak foci, 6 and 5 (ns, P=0.3594), respectively. Mann-Whitney U test calculated no significant difference between numbers of weak foci in wild type and total number of foci in *Prr19*$^{-/-}$ spermatocytes (ns, P=0.5161). Source data are provided as a Source Data file.

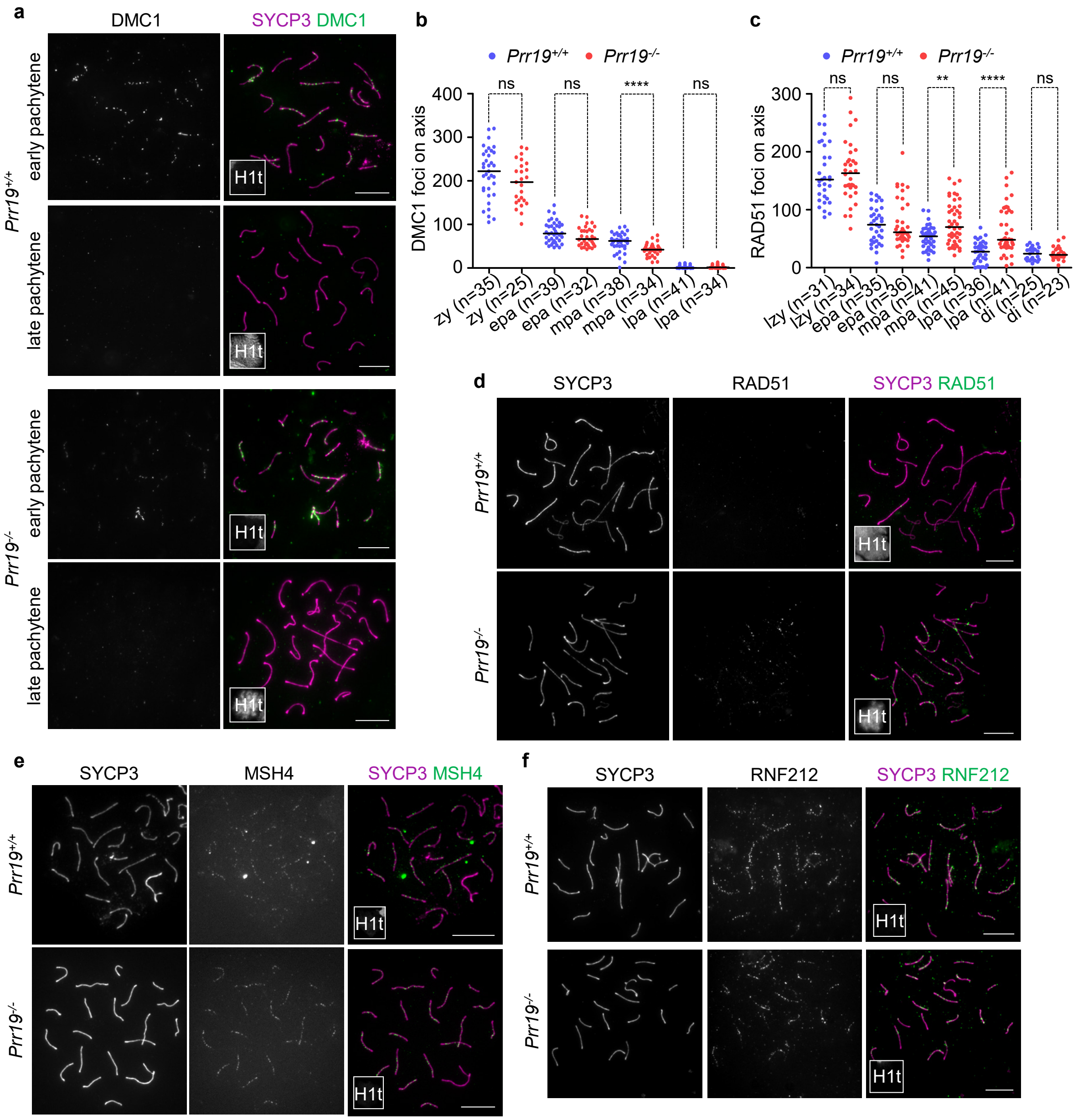

Supplementary Figure 8. DSB repair is delayed in PRR19-deficient cells.
(**a, d, e, f**) Indicated proteins were detected by immunofluorescence in nuclear surface-spread spermatocytes. Miniaturised images of H1t signal are shown in the bottom left corner of overlay images. DMC1 (**a**) staining is shown in early and late pachytene cells; RAD51 (**d**) staining is shown in late pachytene cells. MSH4 (**e**) and RNF212 (**f**) are shown in early pachytene cells; for respective staining in late pachytene cells and quantification of MSH4 and RNF212 focus numbers refer to Fig. 5e-h. Bars, 10 µm. (**b-c**) Quantification of DMC1 (**b**) and RAD51 (**c**) in mid/late zygotene (zy, for DMC1) or late zygotene (lzy, for RAD51), early (epa), mid (mpa), late pachytene (lpa) and diplotene (di) cells. Numbers of analysed cells (n) from two experiments and medians (bars) are indicated. Median focus numbers were compared using Mann-Whitney U test and are as follows in wild type and *Prr19*$^{-/-}$: DMC1 in zy 222 and 197 (P=0.1086), epa 79 and 66.5 (P=0.07598), mpa 62 and 42 (P=6.46E-05), lpa 0 and 1.5 (P=0.2335), RAD51 in lzy 152 and 163 (P=0.6935), epa 74 and 61 (P=0.9267), mpa 54 and 70 (0.002266), lpa 27.5 and 48 (P=7.88E-06), di 24 and 22 (P=0.7101), respectively. ns, ** and **** indicate no significance (P>0.05), 0.01>P>0.001 and P<0.0001, respectively. Source data are provided as a Source Data file.

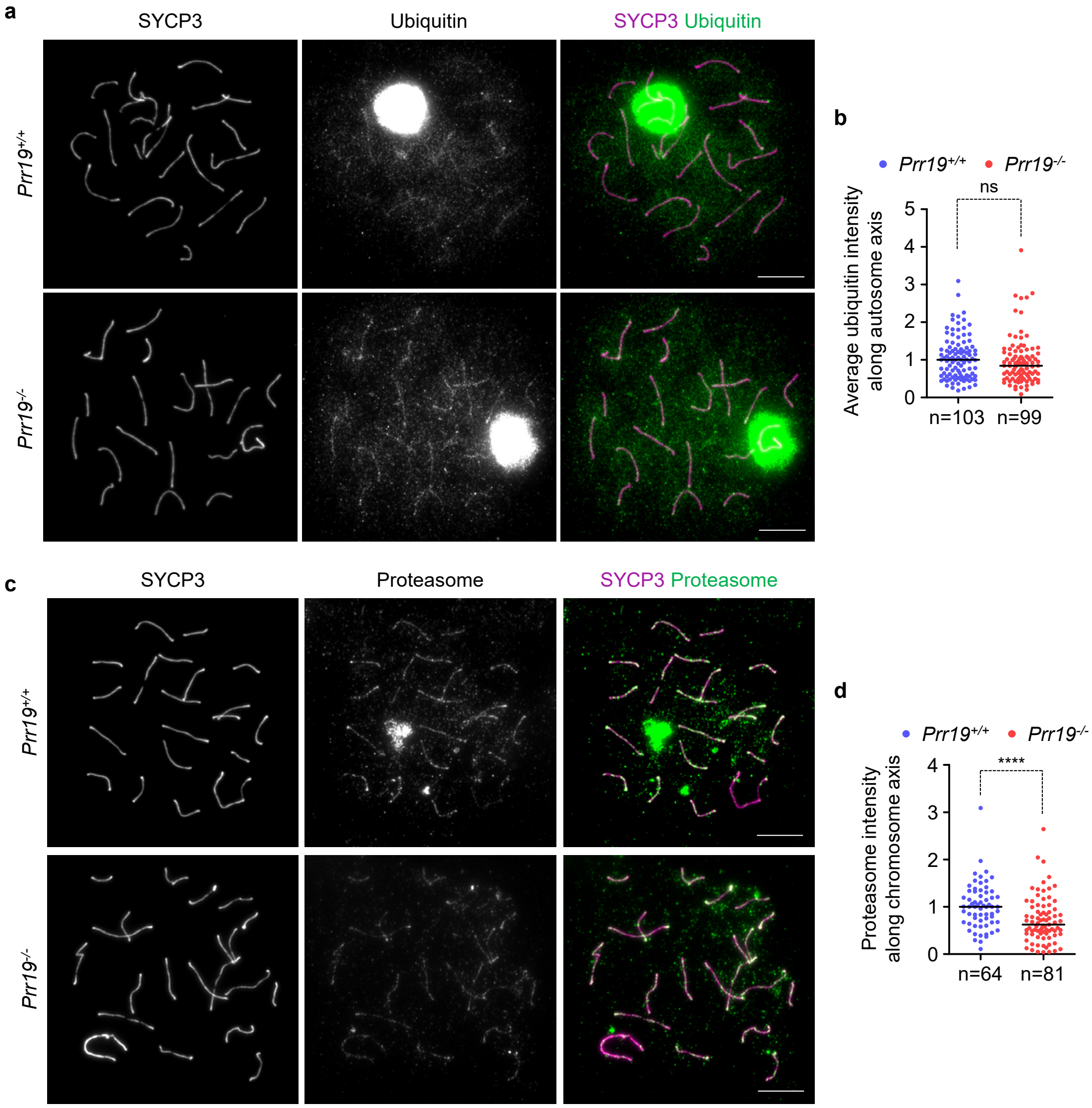

Supplementary Figure 9. PRR19 is not required for ubiquitin and proteasome recruitment to the chromosome axis.
(**a, c**) Mono- and polyubiquitynated conjugates (Ubiquitin, **a**), or proteasome 20S α- and β-subunits (Proteasome, **c**) were detected by immunofluorescence in nuclear surface-spread pachytene spermatocytes. Saturated ubiquitin signal corresponds to the silenced chromatin of sex chromosomes (sex body), where ubiquitin localises in pachytene spermatocytes, as reported before. Bars, 10 µm. (**b, d**) Ubiquitin (**b**) and proteasome (**d**) signal intensities were quantified along chromosome axes. (**b**) Only autosomes outside sex body were taken into account for ubiquitin signal quantification; average intensity per chromosome axis area was calculated. (**d**) The total signal along all chromosomes was used for proteasome intensity quantification. Graph shows pooled data from five and four experiments for ubiquitin and proteasome measurements, respectively. As absolute intensities of ubiquitin and proteasome varied between experiments, the values were normalised to the median of wild-type value within each experiment and normalised values were pooled together. Numbers of analysed cells (n) and medians (bars) are indicated. Mann-Whitney U test indicates no significant difference (ns, P=0.1311) between medians of normalised ubiquitin levels in wild-type and *Prr19*$^{-/-}$ samples (1 and 0.84, respectively), and indicates significant difference (****, P=8.08E-05) between medians of normalised proteasome levels in wild-type and *Prr19*$^{-/-}$ samples (1 and 0.62, respectively). Source data are provided as a Source Data file.

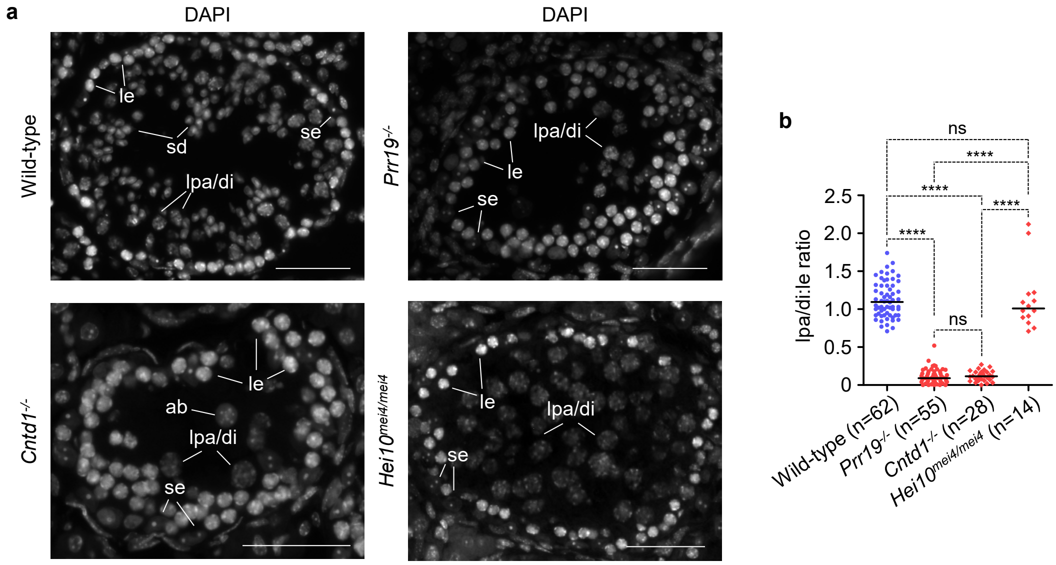

Supplementary Figure 10. Meiotic arrest in *Prr19*$^{-/-}$ and *Cntd1*$^{-/-}$ testes is distinct from meiotic arrest in HEI10-deficient mice.
(**a**) DAPI staining of DNA in testis sections from mice of indicated genotypes. Stage IX seminiferous tubules are shown. Sertoli cells (se), leptotene (le) and late pachytene/diplotene (lpa/di) spermatocytes, elongating spermatids (sd) and cells with abnormal nucleus morphology (ab) are marked. Bars, 50 μm. (**b**) The ratios of late pachytene-diplotene to leptotene (lpa/di:le) spermatocyte numbers were quantified in stage IX seminiferous tubules in testis sections from adult wild-type mice and indicated mutants. Numbers of analysed tubules (n) and medians (bars) are indicated. Analysis from two *Cntd1*$^{-/-}$ and one *Hei10*$^{mei4/mei4}$ animals are shown. Quantifications of wild-type and *Prr19*$^{-/-}$ tubules are from Fig. 3e. Median ratios were as follows: 1.09 in wild type, 0.09 in *Prr19*$^{-/-}$, 0.11 in *Cntd1*$^{-/-}$, and 1.01 in *Hei10*$^{mei4/mei4}$. Mann-Whitney U test, no significant differences (ns) between *Prr19*$^{-/-}$ and *Cntd1*$^{-/-}$ (P=0.3703), and between wild type and *Hei10*$^{mei4/mei4}$ (P=0.338), significant differences (****) between wild type and *Cntd1*$^{-/-}$ (P=3.864E-14), between *Prr19*$^{-/-}$ and *Hei10*$^{mei4/mei4}$ (P=9.15E-09) and between *Cntd1*$^{-/-}$ and *Hei10*$^{mei4/mei4}$ (P=1.7E-07). Source data are provided as a Source Data file.

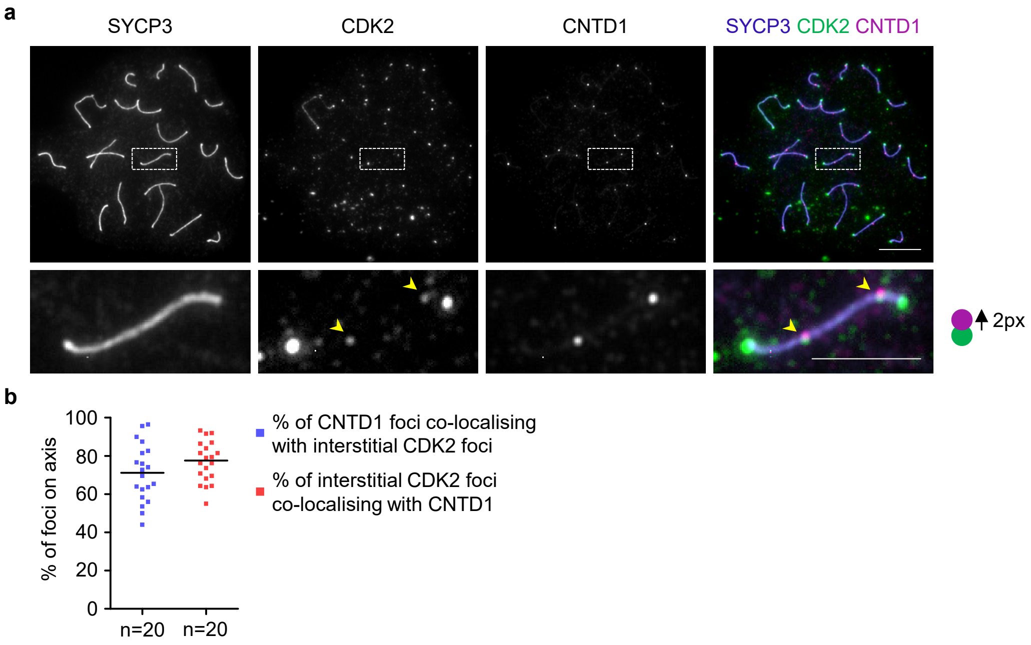

Supplementary Figure 11. CNTD1 co-localises with crossover-specific CDK2 foci.
(**a**) Indicated proteins were detected by immunofluorescence in nuclear spread mid pachytene spermatocytes. Enlarged insets (bottom panel) show co-localisation of CNTD1 and crossover-specific CDK2 interstitial foci (marked by arrowheads). CNTD1 signal is shifted by two pixels up for better visualisation of co-localisation. Bar, 10 µm; in enlarged inset, 5 µm. (**b**) Quantification of co-localisation between CNTD1 and CDK2 foci on autosomal chromosome axes in mid pachytene wild-type spermatocytes, medians (bars) are 71.2% (blue dataset) and 77.6% (red dataset). Numbers of cells (n) from a single mouse are indicated. Source data are provided as a Source Data file.

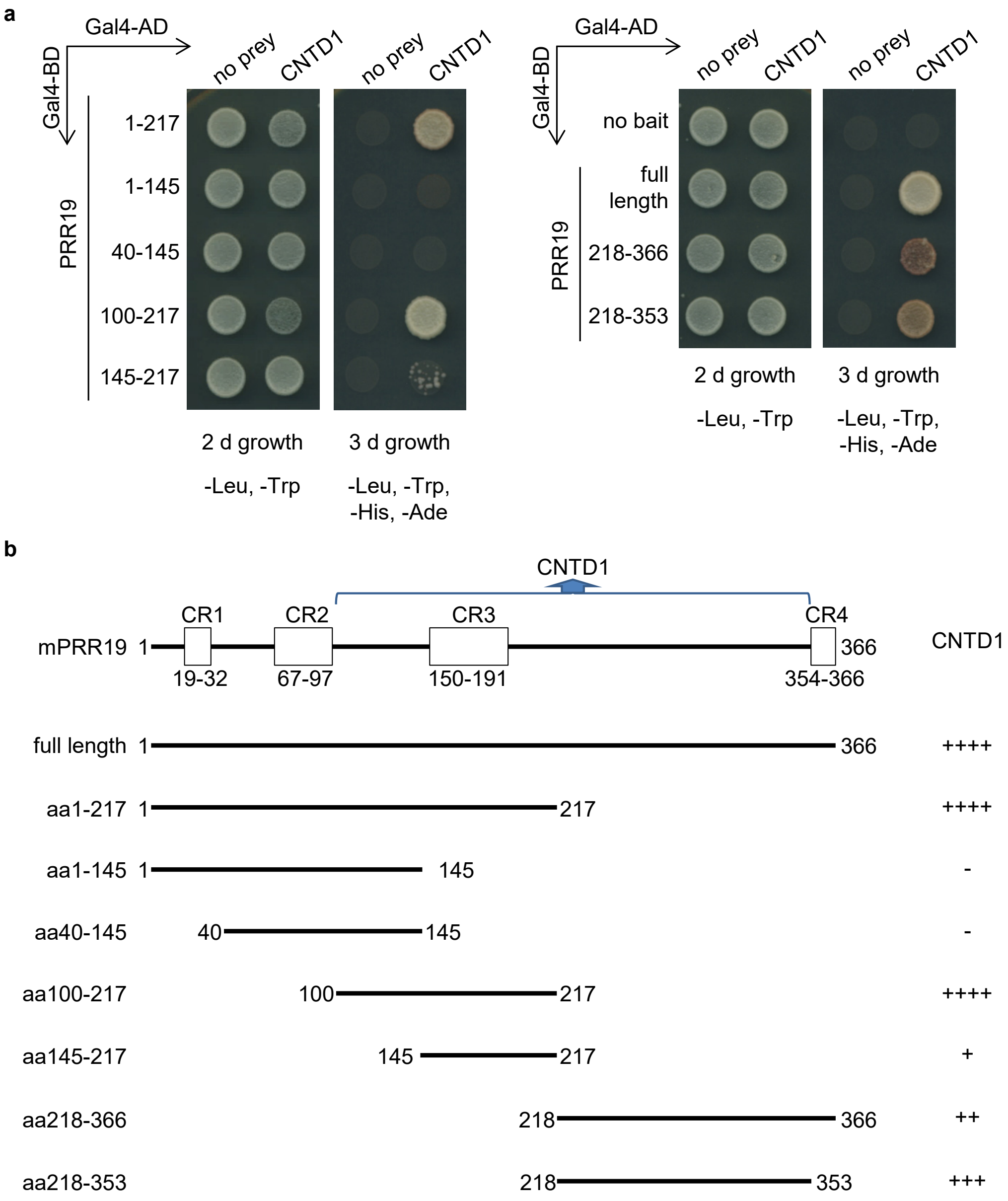

Supplementary Figure 12. Y2H interactions of PRR19 fragments with CNTD1.
(**a**) Y2H assays testing interactions between CNTD1 and fragments of PRR19 (amino acid positions of fragment ends are indicated). Yeast cell suspensions were plated onto dropout plates and imaged after 2 or 3 days of growth. As a negative control, interactions were tested between proteins of interest and either Gal4-binding (Gal4-BD) or Gal4-activation (Gal4-AD) domains that were not fused to bait (no bait) or prey (no prey) proteins, respectively. The two panels represent images of distinct sections of the same dropout plate. (**b**) Schematics of PRR19 domain structure and a summary of CNTD1 interactions with PRR19 fragments. Boxes mark the position of the four conserved regions (CR1-4) that are also indicated in Fig. S2. Numbers represent amino acid positions. The region that appears important for interaction with CNTD1 is indicated. Lines below the schematics of PRR19 structure represent PRR19 fragments that were tested in Y2H for interaction with CNTD1. Strength of Y2H interactions is graded from no interaction (-) to very strong interaction (++++).

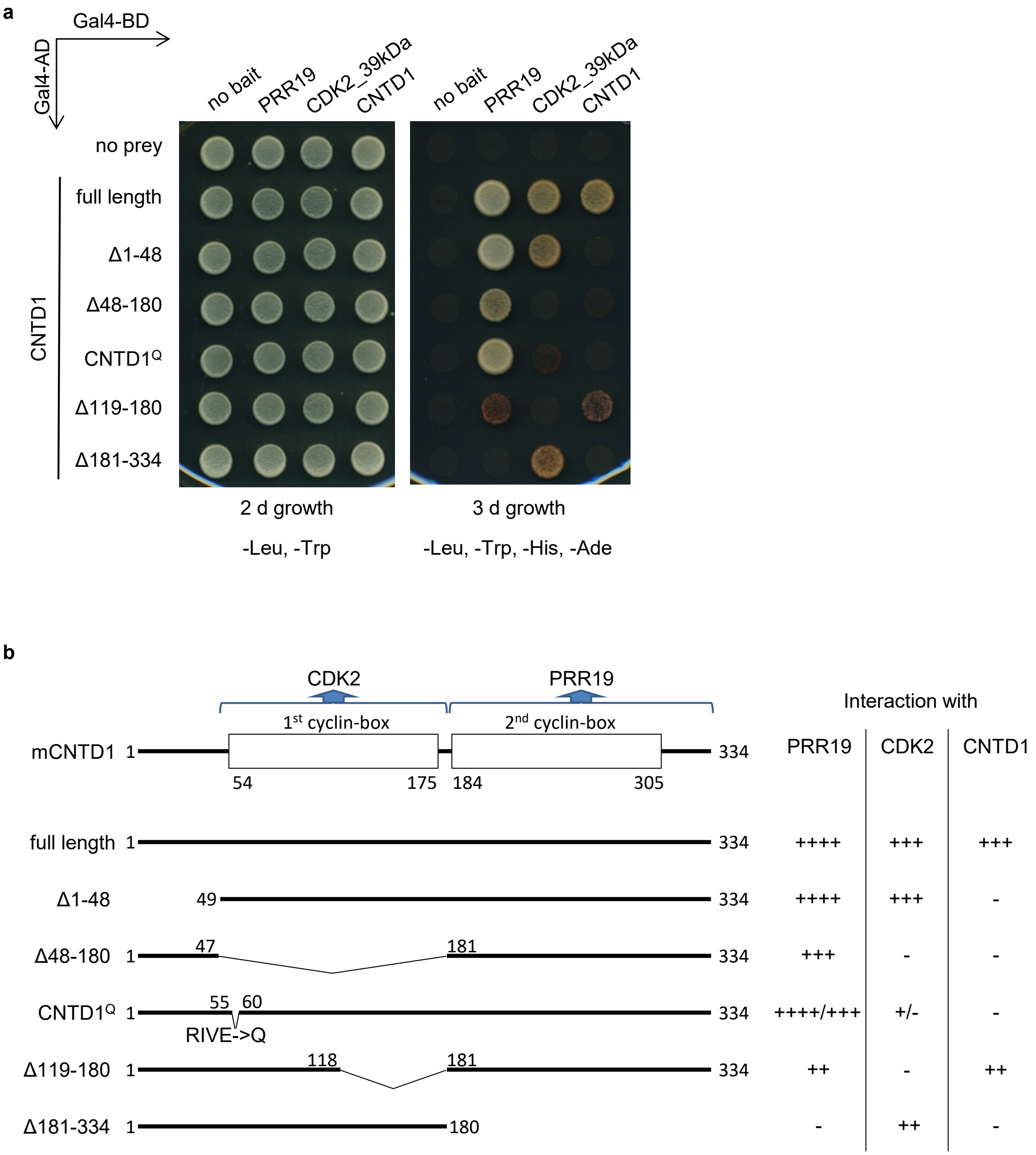

Supplementary Figure 13. Y2H interactions of CNTD1 fragments with PRR19, CDK2 and full length CNTD1.
(**a**) Y2H assays testing interactions between indicated proteins and CNTD1 fragments (amino acid positions of deleted fragment ends are indicated). Yeast cell suspensions were plated onto dropout plates and imaged after 2 or 3 days of growth. As a negative control, interactions were tested between proteins of interest and either Gal4-binding (Gal4-BD) or Gal4-activation (Gal4-AD) domains that were not fused to bait (no bait) or prey (no prey) proteins, respectively. (**b**) Schematics of CNTD1 domain structure based on CNTD1 alignment with cyclin A2 by SWISS-MODEL (https://swissmodel.expasy.org/)[71-73]. Boxes mark the position of the predicted first and second cyclin boxes. Numbers represent amino acid positions. The CNTD1 regions that appear important for interaction with PRR19 and CDK2 are indicated. Lines below the schematics of CNTD1 structure represent CNTD1 fragments that were tested in Y2H for interaction with PRR19, CDK2 and full length CNTD1 as shown in **a** and Fig. 7i. Strength of Y2H interactions is graded from no interaction (-) to very strong interaction (++++), (+/-) indicates a very weak interaction that was detected in CDK2 39kDa bait-CNTD1^{Q} prey but not CDK2 39kDa prey-CNTD1^{Q} bait combinations. Note that the deleted amino acids were replaced by the GGSGSG linker sequence in the Δ48-180 and Δ119-180 CNTD1 variants.

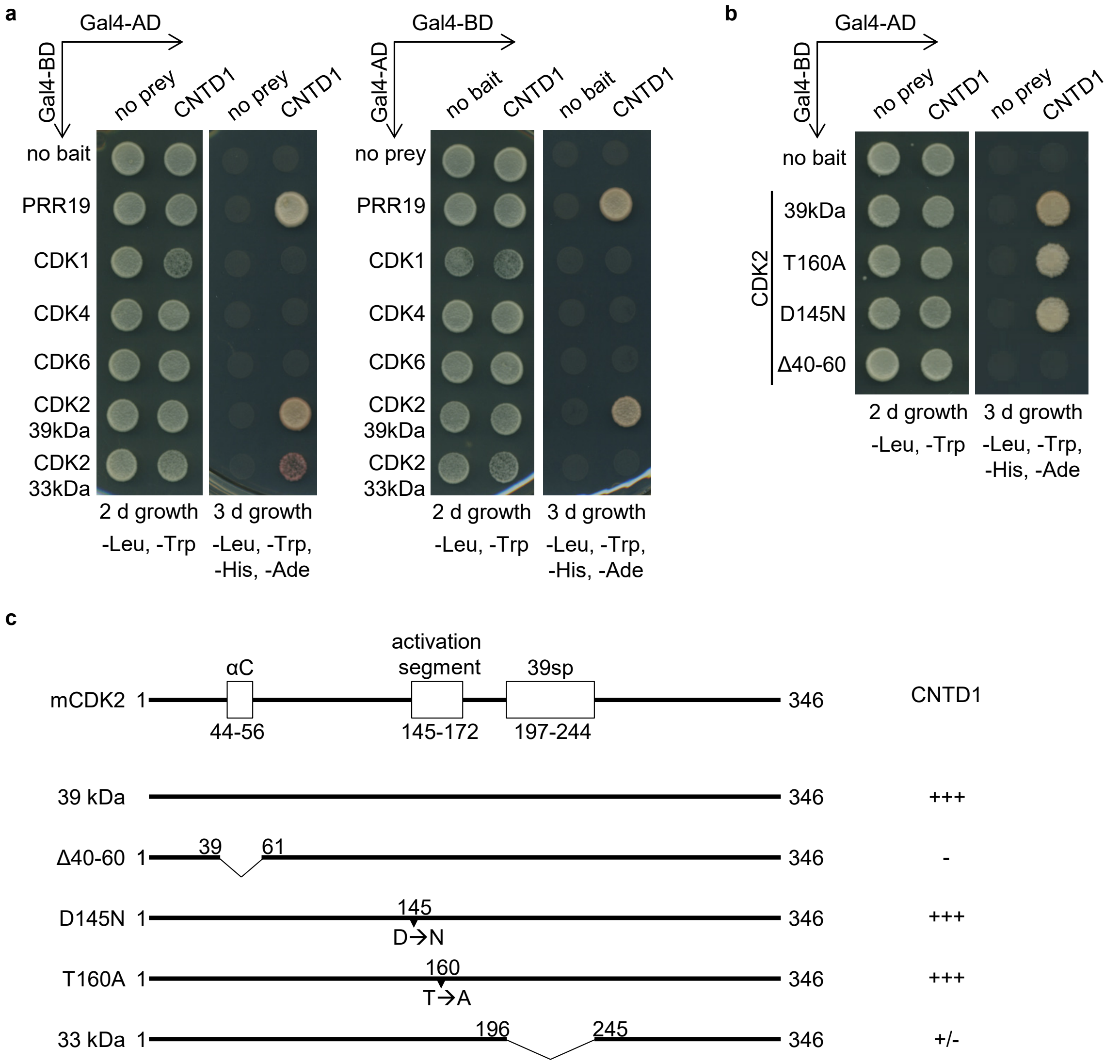

Supplementary Figure 14. Y2H interactions between CNTD1 and full length cell-cycle regulating CDKs and altered versions of CDK2.
(**a** and **b**) Y2H assays testing interactions between CNTD1 and wild type versions of various CDKs (a) or modified versions of CDK2 (b). T160A and D145N represent CDK2 versions where threonine was changed to alanine and aspartate was changed to asparagine in the 160th and 145th amino acid positions, respectively. Δ40-60 represents a CDK2 version where amino acids were deleted between the 39th and 61st positions. Yeast cell suspensions were plated onto dropout plates and imaged after 2 or 3 days of growth. As a negative control, interactions were tested between proteins of interest and either Gal4-binding (Gal4-BD) or Gal4-activation (Gal4-AD) domains that were not fused to bait (no bait) or prey (no prey) proteins, respectively. (**c**) Schematics of CDK2 domain structure. Boxes represent the PSTAIRE alpha helix (αC), the activation segment (act) and the region that is specific to the 39 kDa form of CDK2 (39sp). Numbers represent amino acid positions. Lines below the schematics of CDK2 structures represent CDK2 variants that were tested in Y2H for interaction with full length CNTD1 as shown in **a** and **b**. Strength of Y2H interactions is graded from no interaction (-) to strong interaction (+++), (+/-) indicates a weak interaction that was detected in CDK2 33kDa bait-CNTD1 prey but not CDK2 33kDa prey-CNTD1 bait combinations.

Genotype	Epithelial cycle stage	Total number of tubules	Number of pachytene/diplotene spermatocytes per tubule				Number of cleaved-PARP-positive pachytene/diplotene spermatocytes per tubule			
			>20	10-20	1-9	0	>20	10-20	1-9	0
Prr19$^{+/+}$	I-IV	75	75	0	0	0	0	0	0	75
	V-VI	35	35	0	0	0	0	0	1	34
	VII-VIII	47	47	0	0	0	0	0	1	46
	IX	29	29	0	0	0	0	0	2	27
	X-XII	65	65	0	0	0	0	0	1	64
Prr19$^{-/-}$	I-IV	37	37	0	0	0	0	0	0	37
	V-VI	26	23	3	0	0	2	7	10	7
	VII-VIII	67	10	29	27	1	5	21	39	2
	IX	38	0	7	25	6	0	5	27	6
	X-XII	45	0	1	41	3	0	0	36	9

Supplementary Table 1. Quantification of total or cleaved-PARP-positive cell numbers in the second luminal layer of spermatocytes in seminiferous tubules of adult *Prr19*$^{+/+}$ and *Prr19*$^{-/-}$ mice.

The second column indicates the epithelial cycle stage of seminiferous tubules in which spermatocytes were quantified. Column 3 shows total numbers of inspected seminiferous tubules at the indicated epithelial cycle stages in testes of two mice of each genotype. Columns 4-7 show the numbers of seminiferous tubules that had more than 20, 10-20, 1-9 or no pachytene or diplotene spermatocytes in the second luminal layer. Columns 8-11 refer to the same analysed seminiferous tubules and show the numbers of seminiferous tubules that had more than 20, 10-20, 1-9 or no cleaved-PARP-positive pachytene or diplotene cells in the second luminal layer of spermatocytes. Seminiferous tubules were staged based on immunofluorescence staining of histone H1t and chromatin morphology of spermatogenic cells as detected by DAPI staining in cryosections of testes (see Methods). Cleaved-PARP was detected by immunofluorescence.

Antibody	Source	WB	IF	IP
Gp anti-PRR19	This study, A. Tóth	1:3000	1:1000	1.5/3 µg
Rb anti-PRR19	This study, A. Tóth	1:1000	1:500	
Rb anti-CNTD1	This study, A. Tóth	1:3000	1:500	
Gp anti-CNTD1	This study, A. Tóth			3 µg
Rb anti-histone H3	Abcam, ab18521	1:200000		
Mm anti-GAPDH monoclonal (6C5)	Santa Cruz, sc-32233	1:1000		
Mm anti-α-tubulin monoclonal (DM1A)	Sigma, T6199	1:500		
Gp IgG isotype control	NBP1-97036, Novus Biologicals			3 µg
Mm anti-CDK2 monoclonal (D-12)	Santa Cruz, sc-6248	1:500	1:200	
Mm anti-CCNB1IP1 (HEI10) monoclonal (4H3)	Abcam, ab118999	1:1000	1:150	
Mm anti-SYCP3 monoclonal	Gift from R. Jessberger[82]		1:2	
Ch anti-SYCP3	A. Tóth[33]		1:600	
Rb anti-H1t	A. Tóth[34]		1:20000	
Gp anti-H1t	A. Tóth[34]		1:20000	
Gp anti-H1t	Gift from M. A. Handel[36]		1:500	
Mm anti-MLH1 monoclonal (4C9C7)	Cell Signaling, #3515		1:50	
Rb anti-MLH1	Calbiochem, PC56		1:50	
Rb anti-cleaved PARP	Cell Signaling, #9544S		1:250	
Ch anti-SYCP1	A. Tóth[34]		1:300	
Rb anti-MLH3	Gift from P. Cohen[12]		1:1000	
Mm anti-γH2AX monoclonal (JBW301)	Millipore, #05-636		1:6000	
Rat anti-RPA32/RPA2 monoclonal (4E4)	NEB, #2208		1:100	
Rb anti-DMC1	Santa Cruz, sc-22768		1:200	
Mm anti-RAD51 monoclonal (51RAD01 (3C10))	Thermo Fisher, MA5-14419		1:200	
Rb anti-MSH4	Abcam, ab58666		1:200	
Gp anti-RNF212	N. Hunter[20]		1:50	
Goat anti-RNF212	This study, N.Hunter		1:100	
Mm anti-mono- and polyubiquitinylated conjugates monoclonal (FK2)	Enzo Life Sciences, BML-PW8810-0100		1:500	
Rb anti-proteasome 20S alpha+beta	Abcam, ab22673		1:200	
Rb anti-DDX4/MVH	Abcam, ab13840		1:500	
Mm anti-p63 monoclonal (4A4)	Biocare Medical, CM163A		1:300	

Supplementary Table 2. List of primary antibodies.

Primary antibodies (the first column), their source (the second column) and dilution used for immunoblotting (WB, the third column), immunofluorescence staining (IF, the fourth column) or immunoprecipitation (IP, the fifth column). Gp, guinea pig; Rb, rabbit; Mm, mouse; Ch, chicken.

Antibody	Source	WB	IF
HRP goat anti-mouse	Jackson Immunoresearch, 115-035-166	1:10000	
HRP goat anti-rabbit	Jackson Immunoresearch, 111-035-144	1:10000	
HRP goat anti-guinea pig	Jackson Immunoresearch 106-035-003	1:10000	
HRP mouse monoclonal anti-rabbit IgG, light chain specific	Jackson Immunoresearch 211-032-171	1:10000	
Alexa Fluor® 405 goat anti-mouse	Invitrogen, A31553		1:400
Alexa Fluor® 405 goat anti-rabbit	Invitrogen, A31556		1:400
Alexa Fluor® 405 goat anti-chicken	Abcam, ab175675		1:400
Alexa Fluor® 488 goat anti-guinea pig	Invitrogen, A11073		1:600
Alexa Fluor® 488 goat anti-mouse	Invitrogen, A11029		1:600
Alexa Fluor® 488 goat anti-rabbit	Invitrogen, A11034		1:600
Alexa Fluor® 488 goat anti-rat	Life Technologies, 11006		1:600
Alexa Fluor® 488 goat anti-chicken	Invitrogen, A11039		1:600
Alexa Fluor® 488 donley anti-goat	Invitrogen, A11055		1:600
Alexa Fluor® 568 goat anti-chicken	Invitrogen, A11041		1:600
Alexa Fluor® 568 goat anti-rabbit	Invitrogen, A11036		1:600
Alexa Fluor® 568 goat anti-guinea pig	Invitrogen, A11075		1:600
Alexa Fluor® 568 goat anti-mouse	Invitrogen, A11031		1:600
Alexa Fluor® 568 donkey anti-mouse	Invitrogen, A10037		1:600
DyLight™ 405 donkey anti-guinea pig	Jackson Immunoresearch, 706-475-148		1:200

Supplementary Table 3. List of secondary antibodies.

Secondary antibodies (the first column), their source (the second column) and dilution used for immunoblotting (WB, the third column) or immunofluorescence staining (IF, the fourth column).

Name	Sequence (5'-to-3')	Purpose
**Primers used to produce gRNAs**		
Prr19_14nrv_top	TAGGAGGATCCTGACGGGCCAA	cloning gRNA Prr19_14nrv for CRISPR/Cas9nickase (D10A) targeting of Prr19
Prr19_14nrv_bot	AAACTTGGCCCGTCAGGATCCT	
Prr19_14nfw_top	TAGGATCCATGTGTGATCCTCC	cloning gRNA Prr19_14nfw for CRISPR/Cas9nickase (D10A) targeting of Prr19
Prr19_14nfw_bot	AAACGGAGGATCACACATGGAT	
DR274_F	AAATGGTCAGTATTGAGCCTCAG	to amplify template for IVT to produce gRNA
DR274_R	AAAAGCACCGACTCGGTGCCAC	
Cntd1_gRNA#1	GAAATTAATACGACTCACTATAGGTTCAGGGAGACCCGG ATCGTGTTTTAGAGCTAGAAATAGC	to produce template for *Cntd1*_gRNA #1 without cloning
gRNA_CRISPR_rv	AAAAGCACCGACTCGGTGCCACTTTTTCAAGTTGATAAC GGACTAGCCTTATTTTAACTTGCTATTTCTAGCTCTAAAA C	universal primer to produce template for gRNA without cloning
cntd1#1_MluI_A	AAACCTGACTCCCCACTCTGCATCCACACCTACTATACG CGTCTCCCTGA	single-stranded DNA oligo used as a template for homology-driven recombination
cntd1#1_MluI_A2	CCTGACTCCCCACTCTGCATCCACACCCACTATACGCGT TTCCCTGAAGC	single-stranded DNA oligo used as a template for homology-driven recombination
**Primers used to genotype mice**		
Prr19gen_fw	GCACCACAAGGACTGGCTATTGTC	genotyping of F0 animals, *Prr19*$^{+}$, *Prr19*^{Mut1} and *Prr19*^{Mut2} alleles
Prr19gen_rv	GAGTTGGTACTGCAACTCCTCCAG	
Cntd1gen_fw	CGAAGCGAGGGCAAACATAT	genotyping of F0 animals, *Cntd1*$^{+}$ and *Cntd1*^{Q} alleles
Cntd1gen_rv	CATCTGTCAATTCCAGGCCAG	
Cntd1rv_WT	CTGCATCCACACCCACGAT	genotyping of *Cntd1*$^{+}$ and *Cntd1*$^{-}$ alleles (in combination with Cntd1gen_fw)
Cntd1rv_mut	CTGCATCCACACCCACGAA	
Rnf212 exon forward	CGCTGGAATGAACGCAGGCGC	genotyping of *Rnf212*$^{+}$ allele
Rnf212 exon reverse	CAGGGGAGTGAAGCCACGGTC	
pH530	TCCATGGGCTTAAACCAGTGC	genotyping of *Rnf212*$^{-}$ allele
VM3	GCGCATGCTCCAGACTGCCTTG	
Mlh3	CGGTTTCCCACCTTCTCTACATCGTCC	genotyping of *Mlh3*$^{+}$ *and Mlh3*$^{-}$ alleles
Bkos	TTGGGTAACGCCAGGGTTTTCCCAGTC	
Ckos	TCAGAGAAGGAAGCCAGTGTCTGCCAC	
mHei10atg	ATGTCTTTGTGTGAAGACATGCTGCT	genotyping of *Hei10*$^{+}$ *and Hei10*^{mei4} alleles
mei4GenotypeRevWT	CCCAGCCCCTAGGCACTCAC	
mei4GenotypeRevMT	CCCAGCCCCTAGGCACTCAA	
SP16R	ATGTTAGTCGGCACAGCAGTAG	genotyping of *Spo11*$^{+}$ *and Spo11*$^{-}$ alleles
SP15F	TGAGATACATGGAGGAAGATGG	
PRSF2	CTGAGCCCAGAAAGCGAAGGAA	

Sycp1 exon for	TCAGAGCCAATGAGCAGACTGTATTC	genotyping of *Sycp1*$^{+}$ allele
Sycp1 exon rev	CTGAAGTTCCTGAATGGCTTTTCGC	
SYCP1 Neo1 Forward	GACCTGTCCGGTGCCCTGAATG	genotyping of *Sycp1*$^{-}$ allele
SYCP1 Neo9 Reverse	GATGGCTGGCAACTAGAAGG	
MLH1-a	AGGAGCTGATGCTGAGGC	genotyping *Mlh1*$^{+}$ and *Mlh1*^{Lisk} alleles
MLH1-U	TTTCATCTTGTCACCCGATG	
MLH1-T5	GATCTCGACGGTATCGATAAGC	
**Primers used for RT-PCR**		
Prr19_exon1_fw	GAGGCTTGGGGGTCTCTAC	RT-PCR of *Mm Prr19*
Prr19_exon2_rv	CGGTGCTCCCGGCTCAAC	
Cntd1_exon1_fw	ATGAATATGGAAGGACCCTTGAGG	RT-PCR of *Mm Cntd1*
Cntd1_exon2_rv	CTACAGCCTGGTAGCTCACC	
Rps9_fw	GGCCAAATCTATTCACCATGC	RT-PCR of *Mm Rsp9*
Rps9_rv	TAATCCTCTTCCTCATCATCAC	
**Primers used for cloning**		
Prr19 2.Fw	CACCTTACAGGGCTGCCATGGCT	to clone PRR19 fragment Thr183-Tyr366 for antigen preparation
Prr19 2.Rv	TCAGTACAGTCTCATTGGGGG	
Cntd1fr2_LICfw	ATCACCACCACCACAGTGGCTGTCTGACCCTCCTCGACC	to clone CNTD1 fragment Cys126-Thr249 for antigen preparation
Cntd1_LICrv	TGAGGAGAAGGCGCGTTATCACGTGTTAGAGGAAGCGG	
Prr19_ORFstart	ATGGACCCTCGGGGAC	to amplify Prr19 from cDNA from mouse testis
Prr19_ORFend	TCAGTACAGTCTCATTGG	
Prr19_1-366_BamHI_fw	GAATTCCCGGGGATCATGGACCCTCGGGGACCA	to clone Prr19 full length and Prr19 fragments into vectors for yeast-two hybrid
Prr19_1-366_NdeI_rv	GTAGCAGCACCCATATCAGTACAGTCTCATTGGG	
Prr19_1-217_NdeI_rv	GTAGCAGCACCCATATCACCTCTGTCTCTCAGGGAC	
Prr19_1-145_EcoRI_fw	TACCGCGGCCGAATTCATGGACCCTCGGGGACCA	
Prr19_1-145_BamHI_rv	GCAGGTCGACGGATCCTCATGGAGAATTTGGTGGGCCT	
Prr19_40-145_EcoRI_fw	TACCGCGGCCGAATTCATGTTGGCCCGTCAGGATCC	
Prr19_100-217_EcoRI_fw	TACCGCGGCCGAATTCATGGCCTTGGAATCACATACCCC	
Prr19_100-217_BamHI_rv	GCAGGTCGACGGATCCTCACCTCTGTCTCTCAGGGACC	
Prr19_145-217_EcoRI_fw	TACCGCGGCCGAATTCATGCCAGATCTTCCTGTGTTGGG	
Prr19_218-366_BamHI_fw	GAATTCCCGGGGATCATGGCATCTTCTTGTATGGAAG	
Prr19_218-353_EcoRI_fw	TACCGCGGCCGAATTCATGGCATCTTCTTGTATGGAAGTTC	
Prr19_218-353_BamHI_rv	GCAGGTCGACGGATCCTCAGCTCTGAGTTACTACCCAGG	
Cntd1_1-334_EcoRI_fw	TACCGCGGCCGAATTCATGAATATGGAAGGACCCTT	to clone Cntd1 full length, fragments and

Cntd1_1-334_BamHI_rv	GCAGGTCGACGGATCCTCACGTGTTAGAGGAAGCG	mutants into vectors for yeast-two hybrid
Cntd1_del1-48_EcoRI_fw	TACCGCGGCCGAATTCATGACGGGCAGCTTCAGGGAG	
Cntd1_48-180del_fr1_rv	ACCTGATCCAGAGCCACCTCCCGCAGCCTCCTTCA	
Cntd1_119-180del_fr2_fw	GGTGGCTCTGGATCAGGTCTGCCCACTCCTCTGGCAT	
Cntd1_119-180del_fr1_rv	ACCTGATCCAGAGCCACCAAGCTGCTCTTTCATAGC	
Cntd1_1-180aa_BamHI_rv	GCAGGTCGACGGATCCTCAATTTATTTGGAAGTTCAGGGAC	
Cntd1_mutQ_rv	AACTGCGTTTCCCTGAAGCTGCCCGTC	
Cntd1_mutQ_fw	CAGGGAAACGCAGTTTGTTTTTCTCCTGTCTGAACA	
Hei10_EcoRI_fw	TACCGCGGCCGAATTCATGTCTTTGTGTGAAGACATG	to clone Hei10 full length into vectors for yeast two-hybrid
Hei10_BamHI_rv	GCAGGTCGACGGATCCCTAAATTCTTTTTGCTTTAAAGG	
cdk2_fw_EcoRI	TACCGCGGCCGAATTCATGGAGAACTTCCAAAAGGTG	to clone Cdk2 full length and mutants into vectors for yeast-two hybrid
cdk2_rv_BamHI	GCAGGTCGACGGATCCTCAGAGCCGAAGGTGGG	
mCDK2short_fr1_rv	TGCGGGTCACCATTTCAGCAAAGATGCAGCCCA	
mCDK2short_fr2_fw	TGCTGAAATGGTGACCCGCAGGGCCCTATTCC	
mCDK2_del40-60aa_fr1_rv	CGATATTAGGAGTGTCGAGCCGGATCTTCTT	
mCDK2_del40-60aa_fr2_fw	GCTCGACACTCCTAATATCGTCAAGCTGCTGG	
Cdk2-D145N-fr1-rv	CTCTTGCTAGTCCAAAGTTTGCCAGCTTGATGG	
Cdk2-D145N-fr2-fw	AAACTTTGGACTAGCAAGAGCCTTTGGAGTCCC	
Cdk2-T160A-fr1-rv	GGGTCACCACCTCATGAGCGTAAGTTCGGACAG	
Cdk2-T160A-fr2-fw	CGCTCATGAGGTGGTGACCCTGTGGTACCG	
Cdk1_fw_EcoRI	TACCGCGGCCGAATTCATGGAAGACTATATCAAAATAGAGAAAATTG	to clone Cdk1 into vectors for yeast-two hybrid
Cdk1_rv_BamHI	GCAGGTCGACGGATCCCTACATCTTCTTAATCTGATTGTCCAAG	
Cdk4-FL_FW_EcoRI	TACCGCGGCCGAATTCATGGCTGCCACTCGATATGAACC	to clone Cdk4 into vectors for yeast-two hybrid
Cdk4-FL_RV_BamHI	GCAGGTCGACGGATCCTCACTCTGCGTCGCTTTCC	
Cdk6-FL_FW_EcoRI	TACCGCGGCCGAATTCATGGAGAAGGACAGCCTGAGTCG	to clone 6ast-two hybrid
Cdk6-FL_RV_BamHI	GCAGGTCGACGGATCCTCAGGCTGTGTTCAGCTCCG	

Supplementary Table 4. List of primers.

The table lists primers used in the study: primer names (the first column), 5'-to-3' primer sequences (the second column) and the purpose to which primers were used (the third column).

Supplementary Methods

R code for statistical analysis.

Mann-Whitney U test (used in Fig. 1g, j, 3a, e, 4d, f, h, 5d, f, h, 6c-d, h, 7d and Supplementary Fig. 5d-e, 7b, 8b-c, 9b, d, 10b):

```
library(lmerTest)
library(xlsx)

dat <- read.xlsx("your file.xlsx",1)
wilcox.test(dat[[1]], dat[[2]], paired = FALSE)
```

Analysis of deviance using the likelihood-ratio test based on the chi-squared distribution (used in Fig. 3b, 5b and Supplementary Fig. 4g-h, 6a-b, d):

```
## Download the R-package which can be used to fit models with random effects:
library(lmerTest)
library(xlsx)
## Download the data
dat <- read.xlsx("your file name.xlsx",1)
##below is example table format
my.data.example <- data.frame(expand.grid(
  Level = c("a","b" ,"c"),
  Experiment=c("1","2"),
  Type = c("wt","ko"))  )

my.data.example$Y1 <-c(155, 79,11,143,52,65,16,206,55,222,19,91)
my.data.example

## Look at the data:
dat
## The analysis of the data in the column Y1 is now explained in detail:
## In order to test the interaction between Type and Level,
## two models are fit. In the first model, the interaction is present:
model.1.1 <- glmer(Y1 ~ Type + Level + Type*Level + (1|Experiment),family =
poisson, data=dat)
## To see the summary of the model fit:
summary(model.1.1)
## In the second model, the interaction term is not included
model.1.2 <- glmer(Y1 ~ Type + Level + (1|Experiment),family = poisson, data=dat)
## To see the summary of the model fit:
summary(model.1.2)
## The two models are now compared using the anova() command:
anova(model.1.1,model.1.2)
## This command compares the model fit using the likelihood-ratio test.
## Because the p-value of the likelihood ratio test is less than 5%, we
## conclude that the data provide evidence that the interaction is significant.
## Therefore, we have evidence rgar the distibutions of Level are different
## between the two types (wt and ko).
```

One sample t-test (used in Fig. 6j, 7g):

```
library(lmerTest)
library(xlsx)

dat <- read.xlsx("your file.xlsx",1)
t.test(dat[[1]], alternative = "g", mu=20)
```

Fisher's exact test (used in Fig. 6a and Supplementary Fig. 5b-c):

```
dat <- dat <- read.xlsx("your file.xlsx",1)
dat

##below is example table format

my.data.example <- data.frame(expand.grid(
  Level = c("a","b", "c"),
  Experiment=c("1"),
  Type = c("wt","ko"))  )

my.data.example$count <-c(59, 75, 14, 35, 21, 51)

my.data.example

##

F1 <- xtabs(my.data.example$count ~ my.data.example$Type + my.data.example$Level)
fisher.test(F1)
```
