# Supplementary material for: Proline-rich protein PRR19 functions with cyclin-like CNTD1 to promote meiotic crossing over in mouse
Source: Nat Commun. 2020 Jun 18;11:3101. doi: 10.1038/s41467-020-16885-3 (PMC7303132; doi:10.1038/s41467-020-16885-3)
Supplement: Supplementary file 2 — Reporting Summary [file 41467_2020_16885_MOESM2_ESM.pdf]

natureresearch

Corresponding author(s): Attila Toth

Last updated by author(s): 14.05.2020

Reporting Summary

Nature Research wishes to improve the reproducibility of the work that we publish. This form provides structure for consistency and transparency in reporting. For further information on Nature Research policies, see Authors & Referees and the Editorial Policy Checklist.

Statistics

For all statistical analyses, confirm that the following items are present in the figure legend, table legend, main text, or Methods section.

n/a	Confirmed	
☐	☒	The exact sample size (*n*) for each experimental group/condition, given as a discrete number and unit of measurement
☐	☒	A statement on whether measurements were taken from distinct samples or whether the same sample was measured repeatedly
☐	☒	The statistical test(s) used AND whether they are one- or two-sided<br>*Only common tests should be described solely by name; describe more complex techniques in the Methods section.*
☒	☐	A description of all covariates tested
☐	☒	A description of any assumptions or corrections, such as tests of normality and adjustment for multiple comparisons
☐	☒	A full description of the statistical parameters including central tendency (e.g. means) or other basic estimates (e.g. regression coefficient) AND variation (e.g. standard deviation) or associated estimates of uncertainty (e.g. confidence intervals)
☐	☒	For null hypothesis testing, the test statistic (e.g. *F*, *t*, *r*) with confidence intervals, effect sizes, degrees of freedom and *P* value noted<br>*Give P values as exact values whenever suitable.*
☒	☐	For Bayesian analysis, information on the choice of priors and Markov chain Monte Carlo settings
☒	☐	For hierarchical and complex designs, identification of the appropriate level for tests and full reporting of outcomes
☒	☐	Estimates of effect sizes (e.g. Cohen's *d*, Pearson's *r*), indicating how they were calculated

Our web collection on statistics for biologists contains articles on many of the points above.

Software and code

Policy information about availability of computer code

Data collection	Image data collection was performed using AxioVision version 4.8, Zen Pro or Volocity software. PRR19 protein family collection was performed with NCBI-BLASTp searches (blast+ version 2.6.0) in the NCBI nr protein database and the UniProt reference proteome database. Prr19 transcriptome data was retrieved from NCBI-Gene Expression Omnibus and BioProject databases.
Data analysis	GraphPad Prism 7, R version 3.3.3 (code is provided in a Supplementary Methods file), Geneious 5.6.3, Fiji, Adobe Photoshop CS5, Clustal Omega tool, SWISS-MODEL, MAFFT (-linsi v7.427), Jalview version 2, HMMER version 3.2.1, Jpred 3

For manuscripts utilizing custom algorithms or software that are central to the research but not yet described in published literature, software must be made available to editors/reviewers. We strongly encourage code deposition in a community repository (e.g. GitHub). See the Nature Research guidelines for submitting code & software for further information.

Data

Policy information about availability of data

All manuscripts must include a data availability statement. This statement should provide the following information, where applicable:

- Accession codes, unique identifiers, or web links for publicly available datasets
- A list of figures that have associated raw data
- A description of any restrictions on data availability

Image sets underlying quantitative data are available from the corresponding author upon request. All other data supporting the findings of this study are available within the paper, its supplementary files and referenced published datasets. The source data underlying Figs. 1a-b, e, g, j, 2b-d, 3a-c, 4b, d, f, h, 5b, d, f, h, 6a, c-d, h, j and 7c-d, f-h and Supplementary Figs. 1a-c, g, 3b-c, 4g-h, 5b-e, 6a-b, d, 7b, 8b-c, 9b, d and 10b are provided as a Source Data file.
Databases used in the study: ENCODE project (data source, BioProject: PRJNA66167; https://www.ncbi.nlm.nih.gov/gene/623131/?report=expression), Gene Expression Omnibus (GEO) database (https://www.ncbi.nlm.nih.gov/geo/query/acc.cgi?acc=GSE119411), NCBI nr protein database (available at ftp://ftp.ncbi.nlm.nih.gov/blast/db/FASTA/nr.gz), UniProt reference proteome database (available at ftp.uniprot.org).

Field-specific reporting

Please select the one below that is the best fit for your research. If you are not sure, read the appropriate sections before making your selection.

☒ Life sciences ☐ Behavioural & social sciences ☐ Ecological, evolutionary & environmental sciences

For a reference copy of the document with all sections, see nature.com/documents/nr-reporting-summary-flat.pdf

Life sciences study design

All studies must disclose on these points even when the disclosure is negative.

Sample size	No formal sample-size calculations were performed. Nevertheless, sample sizes were chosen based on our past experiences and publications in the field to allow the detection of medium sized effects (equivalent to Cohen´s d 0.4-0.5) with confidence. Given that the examined mutant mice had very severe phenotypes in recombination, meiocyte apoptosis and fertility, the chosen sample sizes were appropriate and justified. For examples see the following papers: Wojtasz et. al. Meiotic DNA double-strand breaks and chromosome asynapsis in mice are monitored by distinct HORMAD2-independent and -dependent mechanisms. Genes Dev 26, 958-973 (2012), Stanzione, M. et al. Meiotic DNA break formation requires the unsynapsed chromosome axis-binding protein IHO1 (CCDC36) in mice. Nat Cell Biol 18, 1208–1220 (2016); Qiao, H. et al. Antagonistic roles of ubiquitin ligase HEI10 and SUMO ligase RNF212 regulate meiotic recombination. Nat Genet 46, 194–199 (2014); Zhang, J. et al. The BRCA2-MEILB2-BRME1 complex governs meiotic recombination and impairs the mitotic BRCA2-RAD51 function in cancer cells. Nat Commun 11, 2055 (2020); Holloway, J.K. et al. Mammalian CNTD1 is critical for meiotic crossover maturation and deselection of excess precrossover sites. J Cell Biol 205 (5), 633–641 (2014).
Data exclusions	No data were excluded from the analyses.
Replication	All phenotypes were observed in at least two animals of each genotype and, unless stated otherwise, quantifications represent analysis of at least two independent animals. All comparisons were made between datasets obtained from animals that were either littermates or matched by age.<br>All yeast-two hybrid assay results were reproduced in at least two independent repetitions of experiments.<br>Replications attempts were successful and produced consistent results both in experiment that involved animals and experiments that did not.
Randomization	Specific randomization methods are not relevant to the study; randomization was achieved by breeding animals and analysing randomly selected meiocytes. Pairwise yeast-two hybrid (Y2H) experiments presented in the manuscript do not require randomization. The same yeast cultures were transformed with the various Y2H vectors, and the transformants were cultured on the same dropout plates or the same period of time under identical conditions. Thus, comparisons of distinct transformants was tightly controlled.
Blinding	Blinding is not relevant to the study as, due to the drastic differences in phenotypes of wild type and mutant animals, even blinded investigator would be able to distinguish between the control and mutant samples. Yeast-two hybrid experiments are well controlled and have an unambiguous visual readout that is not influenced by the experimenter´s knowledge about sample identity. Images representing raw results are also presented in the figures of the manuscript, allowing direct evaluation by readers. Hence, blinding is not necessary.<br>All comparisons in yeast-two hybrid experiments were made between yeast transformed in the same experiment and grown on the same plate, so that the samples can be compared side-by-side without the need for blinding the investigator.

Reporting for specific materials, systems and methods

We require information from authors about some types of materials, experimental systems and methods used in many studies. Here, indicate whether each material, system or method listed is relevant to your study. If you are not sure if a list item applies to your research, read the appropriate section before selecting a response.

Materials & experimental systems

n/a	Involved in the study	
☐	☒	Antibodies
☒	☐	Eukaryotic cell lines
☒	☐	Palaeontology
☐	☒	Animals and other organisms
☒	☐	Human research participants
☒	☐	Clinical data

Methods

n/a	Involved in the study	
☒	☐	ChIP-seq
☒	☐	Flow cytometry
☒	☐	MRI-based neuroimaging

Antibodies

Antibodies used	All the antibodies used in the study are described in Methods sections 'Generation of antibodies', 'Western blotting and protein detection', 'Immunofluorescence on meiocyte nuclear surface spreads', 'Immunofluorescence on gonad sections' and Supplementary Tables 2-3.

Validation

The specificity of guinea pig and rabbit anti-PRR19, rabbit anti-CNTD1 and goat anti-RNF212 antibodies was confirmed by: (1) immunoprecipitations and western blot analysis of protein extracts from testes of wild-type and Prr19-/-, Cntd1-/- and Rnf212-/- mice, respectively; (2) immunostaining of spermatocyte nuclear surface spreads from testes of wild-type and mutant mice. The other commecially available and earlier published antibodies have been already validated in the published papers. Additionally, commecially available and earlier published antibodies used for immunofluorescence were validated by us in immunostaining by comparing their staining patterns with the known localisation patterns of the respective proteins in meiocytes or on the gonads sections. This type of validation was applied to antibodies as follows: Mm anti-CDK2 (Santa Cruz, sc-6248), Mm anti-HEI10 (Abcam, ab118999), Mm anti-MLH1 (Cell Signaling, #3515), Rb anti-MLH1 (Calbiochem, PC56), Rb anti-cleaved PARP (Cell Signaling, #9544S), Mm anti-γH2AX (Millipore, #05-636), Rat anti-RPA32/RPA2 (NEB, #2208), Rb anti-DMC1 (Santa Cruz, sc-22768), Mm anti-RAD51 (Thermo Fisher, MA5-14419), Rb anti-MSH4 (Abcam, ab58666), Mm anti-mono- and polyubiquitinylated conjugates (Enzo Life Sciences, BML-PW8810-0100), Rb anti-proteasome 20S alpha+beta (Abcam, ab22673), Rb anti-DDX4/MVH (Abcam, ab13840), Mm anti-p63 (Biocare Medical, CM163A), Mm anti-SYCP3 (R.Jessberger), Ch anti-SYCP3 (A.Toth), Rb and Gp anti-H1t (A.Toth), Gp anti-H1t (M.Handel), Ch anti-SYCP1 (A.Toth), Gp anti-RNF212 (N.Hunter), Rb anti-MLH3 (P.Cohen). Antibodies used in western blot were validated by us in immunoblot analysis of testis extracts by comparing the electrophoretic mobility of detected protein bands with the known/published electrophoretic mobility of respective proteins: Rb anti-histone H3 (Abcam, ab18521), Mm anti-GAPDH (Santa Cruz, sc-32233), Mm anti-α-tubulin (Sigma, T6199), Mm anti-CDK2 (Santa Cruz, sc-6248), Mm anti-HEI10 (Abcam, ab118999).

Animals and other organisms

Policy information about studies involving animals; ARRIVE guidelines recommended for reporting animal research

Laboratory animals

Mouse strains used in the study: Spo11-/-, Sycp1-/-, Rnf212-/-, Mlh3-/-, Hei10mei4/mei4, Mlh1Lisk/Lisk, Prr19-/-, Cntd1-/- and Cntd1Q/Q. Adult male and female CD1 and C57BL/6JCrl mice were used for breeding Prr19 and Cntd1 mutant lines. Gonads were collected from mice after euthanasia. Cytological experiments were carried out on samples collected from adult male and foetal (18 days post coitum), newborn or adult female mice and female foetuses. Protein extracts were collected either from testes of adult mice or 13 days old male pups. Typically, adult mice were used between 60-150 days of age except for ovary sections where 6-7 weeks old females were used.

Wild animals

The study did not involve wild animals.

Field-collected samples

The study did not involve samples collected from the field.

Ethics oversight

Prr19, Cntd1, Mlh1 mutant mice were used and maintained in accordance with the German Animal Welfare legislation ("Tierschutzgesetz"). All procedures pertaining to animal experiments were approved by the Governmental IACUC ("Landesdirektion Sachsen") and overseen by the animal ethics committee of the Technische Universität Dresden. The licence numbers concerned with the present experiments with mice are DD24-5131/287/1, TV A 8/2017 and TVV 73/2017. Spo11, Sycp1, Rnf212, Mlh3 and Hei10 mutant mice were maintained and used for experimentation according to the guidelines of the Institutional Animal Care and Use Committees of the University of California, Davis.

Note that full information on the approval of the study protocol must also be provided in the manuscript.
